# Systematic processing of ribosomal RNA gene amplicon sequencing data

**DOI:** 10.1093/gigascience/giz146

**Published:** 2019-12-09

**Authors:** Julien Tremblay, Etienne Yergeau

**Affiliations:** 1 Energy Mining and Environment, National Research Council Canada, Montreal, QC H4P-2R2, Canada; 2 Centre INRS-Institut Armand-Frappier, Institut national de la recherche scientifique, 531ad Boul. des Prairies, Laval, QC H7V-1B7, Canada

**Keywords:** rRNA gene amplicons, bioinformatics, metagenomics, High Performance Computing

## Abstract

**Background:**

With the advent of high-throughput sequencing, microbiology is becoming increasingly data-intensive. Because of its low cost, robust databases, and established bioinformatic workflows, sequencing of 16S/18S/ITS ribosomal RNA (rRNA) gene amplicons, which provides a marker of choice for phylogenetic studies, has become ubiquitous. Many established end-to-end bioinformatic pipelines are available to perform short amplicon sequence data analysis. These pipelines suit a general audience, but few options exist for more specialized users who are experienced in code scripting, Linux-based systems, and high-performance computing (HPC) environments. For such an audience, existing pipelines can be limiting to fully leverage modern HPC capabilities and perform tweaking and optimization operations. Moreover, a wealth of stand-alone software packages that perform specific targeted bioinformatic tasks are increasingly accessible, and finding a way to easily integrate these applications in a pipeline is critical to the evolution of bioinformatic methodologies.

**Results:**

Here we describe AmpliconTagger, a short rRNA marker gene amplicon pipeline coded in a Python framework that enables fine tuning and integration of virtually any potential rRNA gene amplicon bioinformatic procedure. It is designed to work within an HPC environment, supporting a complex network of job dependencies with a smart-restart mechanism in case of job failure or parameter modifications. As proof of concept, we present end results obtained with AmpliconTagger using 16S, 18S, ITS rRNA short gene amplicons and Pacific Biosciences long-read amplicon data types as input.

**Conclusions:**

Using a selection of published algorithms for generating operational taxonomic units and amplicon sequence variants and for computing downstream taxonomic summaries and diversity metrics, we demonstrate the performance and versatility of our pipeline for systematic analyses of amplicon sequence data.

## Background

High-throughput sequencing of amplicons of fragments of the 16S, 18S, and ITS ribosomal RNA (rRNA) marker genes has grown into a cornerstone of microbial ecology research activities. Amplicon sequencing is now massively widespread and has been used in large research initiatives such as the NIH-funded Human Microbiome Project [1–3] and Earth Microbiome Project [4].

Despite this, it is still objectively difficult to adequately analyse data [5,6]. Initiatives to provide GUI-based applications have been reported [7–9]. These types of interfaces, by their fundamental nature, are not prone to systematic analysis in a production context involving the processing of high data loads of multiple projects simultaneously.

Efforts to integrate bioinformatic pipelines as a standard tool to establish microbiome profiles in food safety and energy settings are increasingly being reported [10,11] and 16S rRNA marker gene studies are increasingly being reported to be relevant to complement traditional methods in a clinical context [12–16].

The bioinformatic landscape for processing short marker gene amplicon sequencing data contains a wide array of solutions and is dominated by a few open source popular pipelines such as QIIME [17] and Mothur [18]. In order to execute, these pipelines usually require users to use streamlined or pre-defined steps with limited ability for advanced customization or possibility to use specific unsupported third-party software packages. For instance, DADA2 [19], a package for amplicon sequence variant (ASV) generation, is not available in Mothur but is implemented in QIIME2 [20] with only a subset of input parameters accessible to the user compared to much more from the DADA2 original R package. These efforts were and are continuing to be immensely important in democratizing rRNA amplicon data processing, making it possible for uninitiated Linux users to be able to perform their own data analysis. As such, these pipelines are arguably targeting investigators unfamiliar with computer coding and command line execution. This kind of enclosed setting, however, can become a limiting factor for the types of users who are both proficient at code scripting and comfortable in a Linux/command line environment. Moreover, bioinformatic methods are constantly evolving so production bioinformatic pipelines need to be adapted and modified on a regular basis to properly integrate newly published bioinformatic packages.

Bioinformatic pipelines are intrinsically complex, with up to hundreds of steps depending on the input sequencing data type, with some of these steps needing large compute resources to properly execute. It is critical to introduce robust and flexible ways of systematically processing metagenomic sequencing data types (i.e., mainly amplicons and shotgun) in order to increase their adoption in the aforementioned settings. The GenPipes workflow management system, including an implementation of a QIIME-based 16S rRNA amplicon pipeline, was recently published [21]. Here, as a proof of concept, we leveraged GenPipes's capabilities to build AmpliconTagger, a versatile bioinformatic pipeline managing job generation, submission, dependency, and smart restart that can process any type of gene amplicon sequencing data (16S, 18S, ITS rRNA genes and other custom marker or functional genes) of various sequencing configurations integrating multiple bioinformatic packages. We validated our pipeline with 3 QIIME2 workflows (VSEARCH, Deblur, and DADA2) using 2 mock communities datasets for which we know the exact community composition. We then present microbiome profiling results from published short (MiSeq) and long (Pacific Biosciences [PacBio]) amplicon sequencing datasets using 2 operational taxonomic units (OTUs) and 2 ASV algorithms. We also present detailed information on our methodology so that it can be promptly used, adapted, and improved by others.

## Data Description

To document various aspects of AmpliconTagger, we processed 7 published and publicly available datasets of rRNA amplicon sequencing data of various sequencing configuration, targeting various marker genes and regions (Table 1). We aimed to include datasets from a variety of ecosystems: indoor, human gut and oral cavity, soil and water. We also included a novel dataset consisting of a commercial mock community.

**Table 1:** Details of investigated datasets

Study	Targeted gene and region	Average read length of paired assembled fragments^1^ (mean ± standard deviation)	Sequencing configuration	No. of reads	No. of base pairs (Gb)	No. of samples	File size of sequencing data (gzip compressed)
Even mock community (this study)	16S Bacteria/archaea; V4	250.3 ± 0.7 bp	Illumina 2 × 250 bp	1,987,408	0.50	4	375 MB
Staggered mock community [22]	16S Bacteria/archaea; V4	250.2 ± 0.7 bp	Illumina 2 × 250 bp	289,434	0.072	3	30 MB
Indoor microbiome [23]	16S Bacteria; V3–V4 region	No assembled fragments, single-end reads of 151 bp	Illumina 1 × 150 bp	111,093,697	16.8	1.625	6.9 GB
Lake Michigan [24]	18S Eukaryotes; 1181F–1624R	250.5 ± 3.1 bp	Illumina 2 × 150 bp	19,359,618	4.86	89	2.3 GB
Antibiotic-associated diarrhea [25]	16S Bacteria/archaea; V4	250.4 ± 0.8 bp	Illumina 2 × 250 bp	22,003,478	3.3	276	2.9 GB
Plant microbiome transplant [26]	Fungi ITS; ITS1	249.0 ± 7.9 bp	Illumina 2 × 250 bp	30,775,636	7.72	94	4.1 GB
PacBio mock community [27]	16S Bacterial; full length	No assembled fragments, single-end reads of 1,472.7 ± 215.5 bp	PacBio single-end sequencing	86,353	0.13	8	25 MB
Oral microbiota [28]	16S Bacterial; full length	No assembled fragments, single-end reads of 1,2470.2 ± 225.8 bp	PacBio single-end sequencing	689,430	1.01	40	140 MB

^1^These are the reads that are sent for OTU/ASV generation after having been paired-end assembled (for paired-end sequencing) and controlled for quality as described in Methods.

## Analyses

### Experimental design

Each of the datasets described in Table 1 was processed into the AmpliconTagger pipeline, which contains from 91 to 94 jobs depending on the OTU/ASV generation algorithm used and on sequencing configuration (paired vs single-end sequencing data). All datasets were processed following a common core of quality-filtering procedures, but submitted to 2 different methods of OTU generation (Fig. S1) (VSEARCH [29] and DNAclust [30]) and 2 ASV methods (Deblur [31] and DADA2). In addition, short amplicon mock community datasets were also entirely processed in QIIME2 using a VSEARCH, Deblur, and DADA2 workflow to compare our pipeline against a third-party reference method. We also present and discuss community profiling results of long PacBio amplicons using a mock community and a published oral microbiome sequencing dataset (Table 1). More in-depth analyses of common microbial ecology metrics were assessed for each project for each OTU/ASV generation method and are available in Additional File 1 (Fig. S2 to S7). Although we do present some high-level analysis of ASV vs OTU end results, the present study primarily aims at demonstrating the modularity and methodology implemented in AmpliconTagger and less at performing an exhaustive comparison of the OTU/ASV-generating packages that we used. The complete data processing description of AmpliconTagger is described in the Methods section, and the complete set of commands of each job for each data analysis run is available in Additional Files 2 (AmpliconTagger command traces) and 3 (QIIME2 command traces for the 16S V4 region mock community). An exhaustive user guide is available in Additional File 4.

### Validation with mock communities and comparison with third-party reference pipeline

**Figure 1:**
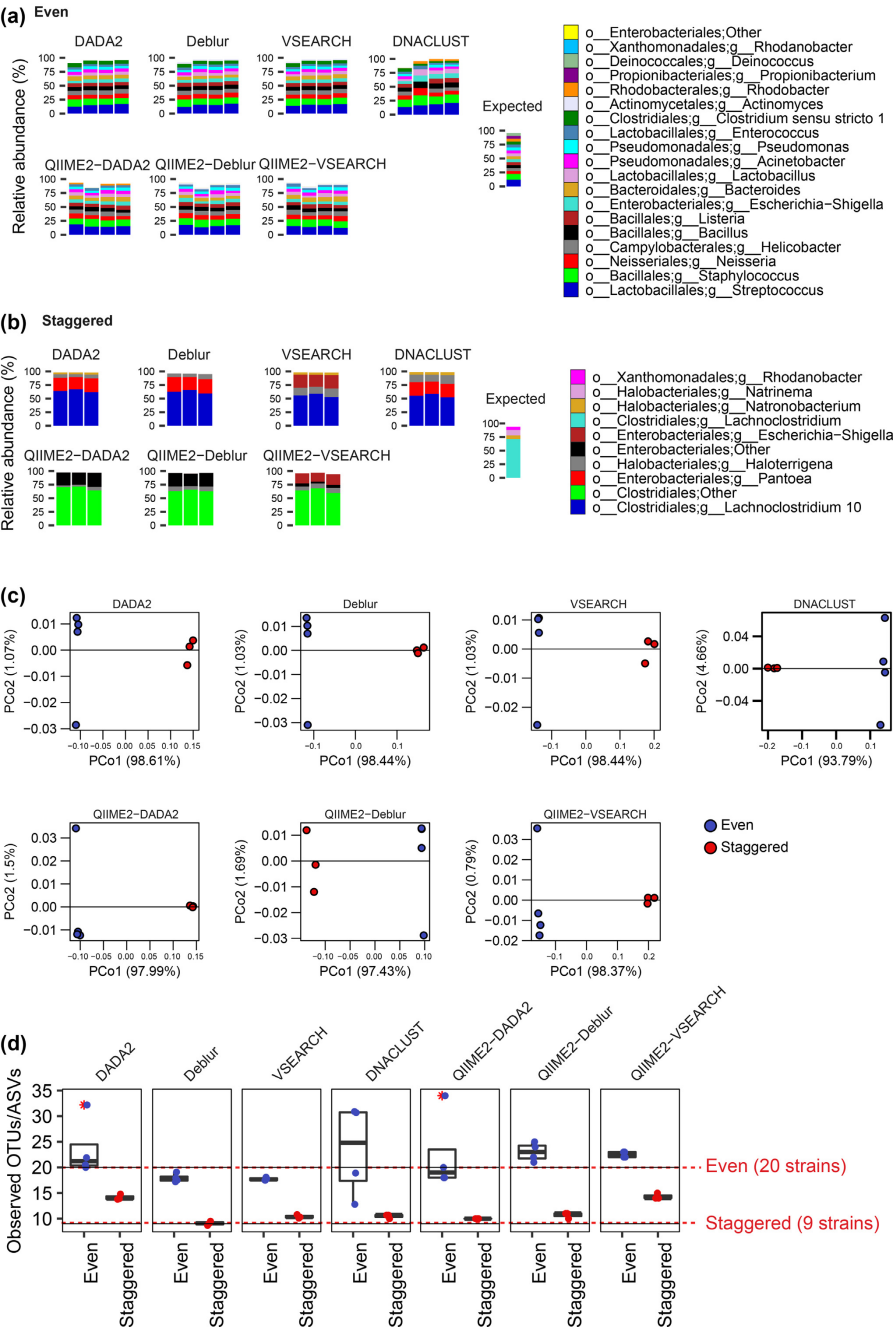
Comparison between Deblur, DADA2, DNACLUST, and VSEARCH as implemented in AmpliconTagger and QIIME2-VSEARCH, QIIME2-DADA2, and QIIME2-Deblur for the taxonomic profiles of (a) even and (b) staggered mock community and (c) β-diversity (weighted UniFrac) and (d) α-diversity of mock community samples (16S V4 region; 2 × 250 bp) where each point represents the Observed OTUs or ASVs indexes of a given sample. Results labelled with the QIIME2- prefix were entirely processed with QIIME2 using either VSEARCH, Deblur, or DADA2 as OTU or ASV generation method. PCo: principal coordinate.

To validate AmpliconTagger, we processed 2 defined mock communities, 1 with even concentrations of 20 bacterial strains (Table S1) and 1 with staggered concentrations of 9 genomes (Table S2). We obtained community profiling results of each mock sample using AmpliconTagger (VSEARCH, DNACLUST, Deblur, and DADA2) and compared them with the end results of the same sequencing libraries, but entirely processed with QIIME2 using VSEARCH, Deblur, and DADA2 workflows (DNACLUST is not implemented in QIIME2). Taxonomic profiles are highly similar across all tested methods (Fig. 1a and b), and minor differences are probably caused by the 2 different Silva R128 training sets used by AmpliconTagger (100% identity sequences) vs QIIME2 (database, clustered at 99% identity). Notably, QIIME2 workflows identified 2 major taxonomic lineages as being assigned to *Clostridiales*; Other and *Enterobacteriales*; Others while AmpliconTagger classified them instead as *Lachnoclostridium* and *Pantoea* (Fig. 1b). Regardless of the methods used, all samples clustered similarly in β-diversity ordinations (Fig. 1c) and show relatively similar α-diversity (Observed OTUs/ASVs index) values (Fig. 1d). We computed the Mantel *r* statistic to assess for correlation between weighted UniFrac and Bray-Curtis distance matrices among all 7 tested methods (Table S3), which generally shows high correlation (>0.8) for most comparisons except for the ones where the QIIME2-DADA2 data were involved.

### Performance

**Figure 2:**
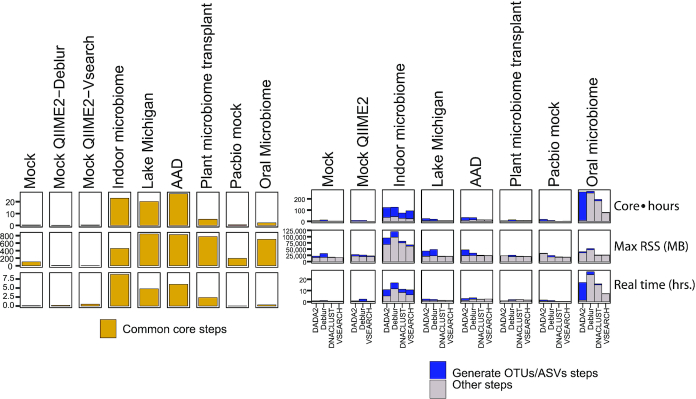
Resource consumption for investigated datasets and each OTU/ASV generation method. There are no common core steps for QIIME2-DADA2 workflow because raw reads were submitted to DADA2 directly.

We compiled compute resources consumed for each of the datasets considered for this study (Fig. 2). The common core of AmpliconTagger consists of steps 1–6 as detailed below in the Methods section. The indoor microbiome, Lake Michigan, and antibiotic-associated diarrhea (AAD) studies consumed similar amounts of core hours (22.7, 20.2, and 26.6 core hours, respectively), while the mock community, oral microbiome, and rhizosphere microbiome transplant studies took significantly less resources, with respective values of 0.53, 2.2, and 5.2 core hours. OTU/ASV generation jobs consumed the most resources for the oral microbiome (PacBio long reads) project followed by the indoor microbiome data, which again is the dataset containing both the highest number of (short) reads and base pairs. For the oral microbiome data, which had a low data input, but long read lengths, DADA2 took the longest time to generate ASVs, with downstream steps completing quickly because of the low number of ASVs generated compared to other OTU or ASV generation methods (Table 2). Generally, OTU/ASV generation and their downstream steps consumed more resources than the common core steps.

**Table 2:** Number of reads and OTUs/ASVs throughout AmpliconTagger's execution

Project	OTU/ASV generation method	Total reads	Contaminant reads	PhiX reads	Non-contaminant and non-PhiX reads	Non-contaminant and non-PhiX reads 1	Non-contaminant and non-PhiX reads 2	Reads 1 QC passed	Assembled reads	Assembled reads QC passed	Clustered or dereplicated sequences	No. of clusters or dereplicated sequences
Mock community (V4 16S; paired-end)	Deblur	2,602,808	8	27	2,276,776	1,138,388	1,138,388	-	1,123,408	1,032,461	597,547	67
	DNACLUST										918,257	67
	VSEARCH										973,750	34
	DADA2							928,310 R1 + 928,310 R2	-	-	885,235	96
	QIIME2-Deblur		-	-	-	-	-	-	966,899	966,829	599,522	46
	QIIME2-VSEARCH		-	-	-	-	-	-	966,899	966,829	916,980	37
	QIIME2-DADA2		-	-	-	-	-	-	-	-	980,184	124
Indoor microbiome (V4 16S; single end)	Deblur	111,093,697	48,996	0	111,044,701	-	-	108,008,427	-	-	73,010,229	27,391
	DNACLUST										95,120,374	18,340
	VSEARCH										100,289,243	14,341
	DADA2										103,031,599	32,647
Lake Michigan (1181F-1624R 18S; paired-end)	Deblur	19,359,618	275,694	4,521	18,803,930	9,401,965	9,401,965	-	8,052,948	3,356,475	2,201,736	662
	DNACLUST										2,629,227	564
	VSEARCH										2,672,395	483
	DADA2							2,522,201 R1 + 2,522,201 R2	-	-	1,880,759	854
AAD (V4 16S; paired-end)	Deblur	22,003,478	151	80	22,001,808	11,000,904	11,000,904	-	10,860,416	9,209,510	5,657,445	1,560
	DNACLUST										7,791,719	1,053
	VSEARCH										8,100,048	827
	DADA2							7,435,547 R1 + 7,435,547 R2	-	-	6,583,440	1,791
Plant microbiome transplant (ITS1 ITS; paired-end)	Deblur	30,775,636	174,471	5,770,656	24,816,770	12,408,385	12,408,385	-	9,850,519	7,479,355	1,901,625	780
	DNACLUST										6,124,824	1,172
	VSEARCH										7,166,333	1,056
	DADA2							3,215,102 R1 + 3,215,102 R2	-	-	2,954,161	1,130
Mock community (full-length 16S; single end)	Deblur	93,905	-	-	93,905	86,353	-	74,485	-	-	6,543	47
	DNACLUST										59,348	1,026
	VSEARCH										60,499	415
	DADA2										50,876	49
Oral microbiome (full-length 16S; single end)	Deblur	627,138	-	-	627,138	562,986	-	562,896	-	-	219,423	199,780
	DNACLUST										520,992	77,305
	VSEARCH										523,444	69,478
	DADA2										262,414	6,535

### Microbial ecology metrics obtained for each dataset

Globally, ecological patterns were similar for the 4 tested methods for all projects (Table 3 and Additional File 1), except for the 2 16S PacBio data types (oral microbiome and PacBio mock data). The numbers of ASVs and OTUs obtained for a given project were fairly similar for short amplicon data types, except for the indoor microbiome data, which yielded 27,391 Deblur ASVs, 32,647 DADA2 ASVs, 18,340 DNACLUST OTUs, and 14,341 VSEARCH OTUs (Table 2). In contrast, the number of ASVs and OTUs obtained with the oral microbiome data with the same 4 methods was more variable with 199,780, 6,535, 77,305, and 69,578, respectively. The total number of reads included in these OTUs/ASVs was 219,423, 6,535, 77,305, and 523,444, respectively. In general the amounts of generated ASVs/OTUs were similar, but ASVs represented fewer total reads.

**Table 3:** Mantel *r* statistics comparing distance matrices of each ASV/OTU generation method for each project

Beta-diversity metric	Deblur vs DNACLUST	Deblur vs VSEARCH	Deblur vs DADA2	DNACLUST vs VSEARCH	DNACLUST vs DADA2	VSEARCH vs DADA2
Weighted UniFrac						
Mock community (V4 16S; paired-end)	0.984	0.999	0.999	0.983	0.986	0.999
Indoor microbiome (V4 16S; single-end)	0.940	0.900	0.905	0.934	0.955	0.956
Lake Michigan (1181F-1624R 18S; paired-end)	0.952	0.956	0.949	0.986	0.977	0.978
AAD (V4 16S; paired-end)	0.943	0.943	0.942	0.968	0.970	0.958
Plant microbiome transplant (ITS1 ITS; paired-end)	0.403	0.508	0.542	0.403	0.465	0.617
Mock community (full-length 16S; single-end)	0.359	0.225	0.461	0.988	−0.078	−0.080
Oral microbiome (full-length 16S; single-end)	−0.097	−0.100	−0.093	0.987	0.958	0.953
Bray-Curtis						
Mock community (V4 16S; paired-end)	0.864	0.580	0.999	0.724	0.865	0.580
Indoor microbiome (V4 16S; single-end)	0.991	0.967	0.996	0.980	0.993	0.972
Lake Michigan (1181F-1624R 18S; paired-end)	0.994	0.996	0.993	0.997	0.993	0.991
AAD (V4 16S; paired-end)	0.960	0.941	0.993	0.980	0.967	0.949
Plant microbiome transplant (ITS1 ITS; paired-end)	0.821	0.812	0.792	0.979	0.935	0.914
Mock community (full length 16S; single-end)	−0.331	−0.241	−0.119	0.901	0.023	0.194
Oral microbiome (full length 16S; single-end)	0.046	0.040	−0.026	0.933	0.415	0.436

Each *r* statistic had a *P*-value < 0.001.

To highlight differences amoung the 4 tested OTU/ASV generation methods, we compared their taxonomic summaries and α- and β- diversities. These results are included in Figures S2–S7, and for clarity and practicality purposes, we narrowed sample selection for each project to a subset of experimental variables. We also computed the Mantel *r* statistic to assess for correlation between weighted UniFrac and Bray-Curtis distance matrices among all 4 tested methods (Table 3). Overall, and consistent with results reported in Figures S2-S7, these results show that weighted UniFrac and Bray-Curtis distances/dissimilarities obtained with all 4 tested methods are quite similar, with *r* statistic values >0.9 for the indoor microbiome, AAD, and Lake Michigan data types. The ITS data (rhizosphere transplant) showed low *r* statistics in the range of 0.4–0.6 between the weighted UniFrac matrices. However, Mantel tests between Bray-Curtis dissimilarity matrices of all 4 OTU/ASV ITS data types gave *r* statistics >0.9 for all comparisons. For the mock community, weighted UniFrac distances of all 4 methods were similar with *r* statistics values >0.9. However, Bray-Curtis dissimilarity matrices were more divergent, with *r* statistics values of 0.580 (Deblur vs VSEARCH), 0.580 (VSEARCH vs DADA2), and 0.724 (DNACLUST vs VSEARCH). Overall, this indicates that the type of distance metric used had a considerable effect on the microbial population structure assessment.

### Validation of procedures with long PacBio reads datatype

Using PacBio full-length amplicon sequencing for microbial community profiling is increasingly making inroads to complement population characterization based on short amplicon sequencing, but bioinformatics procedures are objectively recent and not as mainstream as they are with short-length amplicon sequencing data. We therefore validated our long 16S reads processing methodology with a PacBio mock community library [32] (Table S1). Taxonomic profiles of all 4 tested methods (VSEARCH, DNACLUST, DADA2, and Deblur) are generally similar with some minor differences in low-abundance taxa (Fig. S6a). However, more important discrepancies are actually observed between expected taxa and what was obtained with the actual sequencing libraries (Fig. S6b). The α-diversity metrics were computed, and observed ASVs ranked near the expected number of 23 ASVs whereas observed OTUs were higher than the expected value: 60.6 ± 5.9 for DNACLUST and 172.4 ± 16.2 for VSEARCH. Mantel *r* statistics between DNACLUST and VSEARCH were relatively high (0.851 weighted UniFrac and 0.844 for Bray-Curtis) but weak for the comparisons involving ASV data. For the PacBio oral microbiome data, Mantel *r* statistics computed between the weighted UniFrac distance matrices of all 4 data types (Table 3) showed strong values >0.9 for DNACLUST vs VSEARCH, DNACLUST vs DADA2, and VSEARCH vs DADA2, indicating relatively strong similarity between all ASV and OTU distance matrices when phylogenetic distance is factored into the distance computation. Bray-Curtis matrices, however, showed lower correlations comparing DNACLUST vs DADA2 (0.415) and VSEARCH vs DADA2 (0.436), while DNACLUST vs VSEARCH (0.928) distances were highly correlated. Comparisons involving Deblur data returned low or negative values.

## Discussion

### Backbone for rRNA sequence processing methodology

The primary objective of this report was to showcase AmpliconTagger, a highly modular HPC-oriented pipeline geared for performing bioinformatic analyses of rRNA amplicon data. To facilitate implementation and testing, we shared a Docker repository of a CentOS-7 image with a fully working implementation of AmpliconTagger, which includes data of 20 sequencing libraries from the AAD study, all the databases, training sets, and fully detailed commands of each job for each step of our pipeline. Our workflow also relies on a set of perl scripts and libraries labeled nrc_tools for which the complete code is also available in a code repository. We wish to emphasize that we do not present our methodology as a gold standard but rather as a blueprint of an end-to-end open source working modular pipeline that is able to incorporate virtually any in-house scripts or third-party packages (see Additional File 4 for more details). We also recognize that some steps in our workflow could be improved and even be replaced by other potentially more efficient software or methods, which is highly facilitated by the open modular nature of the pipeline.

### Resources and time consumption

With bioinformatics entering many spheres of research and service fields, there is a need for flexible, scalable, and robust methods to systematically analyse high-throughput nucleic acid sequencing data of all types. Here we present in detail our gene amplicon production workflow with performance metrics and actual results from published rRNA marker gene datasets. We executed our workflow using 2 OTU generation methods, VSEARCH and DNACLUST; and 2 ASV generation methods, Deblur and DADA2. We included VSEARCH because it represents the open source version of the popular USEARCH closed source software. As such, VSEARCH is increasingly being used by the research community as a suitable replacement for USEARCH. Our group started to use DNACLUST as a suitable open source alternative to replace USEARCH some years ago, and since then, we have processed many rRNA marker gene sequencing datasets using this package, which is the reason for its inclusion in this study. The goal of the present study was not necessarily to benchmark these 4 OTU/ASV generation methods but rather to show the modular nature of the pipeline and offer useful metrics of what microbiome profiling results and resource consumption can be expected from using these software packages in the context of our whole workflow methodology.

While short amplicon sequence data processing is not that demanding from a computational resource point of view, a complete working pipeline requires many steps to get from the raw data to key end results. Here we show that quality control (QC) and fastq preprocessing steps of sequencing data prior to OTU/ASV generation consumed the most core hours for datasets that had an appreciable number of reads (indoor microbiome, Lake Michigan, and AAD). The rhizosphere transplant project had a comparable number of reads, but possibly because of short ITS read length (once paired-end assembled), jobs relying on Qscore metrics such as quality filtering and quality score profile compilation quickly completed (Fig. 2). For downstream steps, which correspond to the OTU/ASV generation step to the pipeline completion, the indoor microbiome (single-end 150-bp reads) and oral microbiome (single-end ∼1,470-bp reads) consumed the most resources, the former because of the high raw data load (16.8 Gb) and the latter because of its long read lengths. In terms of memory consumption, the indoor microbiome project, again because of its high amount of raw base pairs, consumed significantly more RAM than the other projects.

### Validation with mock communities

Comparisons of mock community profiling results obtained by our pipeline with the ones obtained with a QIIME2-VSEARCH, -Deblur, or -DADA2 workflow were highly concordant (Fig. 1). All results deriving from either AmpliconTagger or QIIME2 were essentially similar and consistent with the expected taxonomy. The staggered community (Fig. 1b) gave almost identical community structure patterns, but slight differences in taxonomic assignments were observed for the most abundant taxon, which was supposed to be a *Lachnoclostridium* (Fig. 1b—expected panel) but ended up being classified as a *Lachnocolstridium 10* by AmpliconTagger (DNACLUST, VSEARCH, DADA2, and Deblur) and an undefined genus belonging to the *Clostridiales* order for the QIIME2-based workflows. A similar situation is observed with QIIME2 assigning a major taxon to an undefined genus belonging to the *Enterobacteriales* order, while AmpliconTagger assigned it to the *Escherichia-Shigella* genus (Fig. 2b). Even though the 2 training sets used to classify OTUs/ASVs are built from the Silva 128 release, the QIIME2 training set was trained on a 99% identity clustered database and the AmpliconTagger training set was trained on a 100% identity (i.e., unclustered) fasta database, which probably explains the differences observed in taxonomy, which, all things considered, are quite minimal.

### Robustness of ecological patterns between OTU- and ASV-based results

One of the key advantages of the pipeline introduced here is that it is modular and can be customized to fit the research needs of the user. For instance, we have shown here that both ASV and OTU generation methods can be used interchangeably, making the AmpliconTagger pipeline agnostic to the current heated debate about ASV vs OTU. ASV generation is a method that has recently gained traction [33] and is increasingly being adopted as an alternative to OTU-based analyses. Here we processed 7 types of datasets and compared end results of OTU clustering procedures done with DNACLUST and VSEARCH and 2 ASV-based methods (Deblur and DADA2). In most cases in which short reads are being analysed, results given by Deblur and DADA2 are essentially identical to what is obtained by VSEARCH and DNACLUST. One notable exception is for the ITS data type, where the weighted UniFrac Mantel correlations among the 4 methods are significantly lower than what is observed for short 16S and 18S amplicon data types. This discrepancy might be due to the fact that ITS amplicon sequence lengths are quite variable as shown in Fig. S8, with significant amounts of reads dispersed from 200 to 350 bp. During the clustering process, many of the shorter reads are “absorbed” by the larger reads. For ASVs, all these reads of various lengths eventually form a distinct ASV and in consequence many ASVs do not reach the cut-off of ≥25 reads per ASV. This holds true for all projects, where the number of ASV reads is often lower than the number of OTU reads (Table 2), but is probably exacerbated in the ITS data where many of the amplicon sequences are of different lengths (Fig. S8). That said, one would still have expected the weighted UniFrac mantel correlation to be higher between DNACLUST and VSEARCH OTUs—as observed in other data types—which was not the case. On the other hand, Bray-Curtis dissimilarity matrices were highly concordant between Deblur, DADA2, VSEARCH, and DNACLUST for the ITS data type. This implies that global alignments, inherent to generating the Unifrac distance matrix, may be impractical because of the high variability in sequence and length of ITS amplicons, as previously suggested [34].

### PacBio long reads

PacBio taxonomic profiles of the mock community generated by AmpliconTagger were consistent with what was expected (Fig. S6a). While taxonomic profiles obtained with ASVs for the PacBio mock community are generally consistent with what is obtained with OTUs (Fig. S6a), there seems to be a low but consistent proportion of OTUs that are assigned closely related species in addition to the expected targets. For instance, the mock community contained 2 species belonging to the *Staphylococcus* genus (Fig. S6a): *Staphylococcus aureus* and *Staphylococcus epidermidis*. These 2 species have been identified in the OTU and ASV data, but the OTUs were assigned more closely related species such as *Staphylococcus saccharolyticus*, an uncultured *Staphylococcus*, and undefined *Staphylococcus* species (i.e., Other). Regardless of the nature of the data (ASVs or OTUs), taxonomic assignment of PacBio CCS was accurate for the majority of the species of the mock community (Fig. S6a—*Actinomyces, Bacteroides, Clostridium, Deinococcus, Enterococcus, Helicobacter, Listeria, Neisseria, Propionibacterium, Pseudomonas*, and *Streptococcus* panels) but seemed to be more challenging for *Escherichia coli, Bacillus cereus*, and *S. epidermidis*. For instance, we expected to identify the *B. cereus* species but instead observed the *Bacillus anthracis*(VSEARCH, Deblur, and DADA2) and an undefined bacillus (Other) (DNACLUST).

The taxonomic profiles of the more complex oral microbiome samples were overall highly similar to up to the species level with the majority of ASVs/OTUs pointing toward *Streptococcus pseudopneumoniae* and *Streptococcus salivarius* related species. Some differences were also observed in less abundant species: Deblur was enriched in *Streptococcus vestibularis* while DADA2 contained *Streptococcus mitis* and *Streptococcus sanguinis*.

Our results highlight the challenge of properly assigning species taxa to long 16S reads using general databases, in our case the Silva DB. This suggests that in some instances, PacBio CCS reads contain at least enough errors to cause misclassification at the species level when processed as ASVs and that clustering reads (OTU methods) in the objective of correcting these errors also results in misclassification.

Deblur ASVs in the mock community gave poor Mantel *r* correlation with other methods because although it held a total of 6,535 reads, 4 samples did not meet the cut-off of 1,000 reads required in the rarefaction procedure to normalize ASV tables, hence resulting in the rejection of 4 samples and lowering Mantel *r*-correlations involving Deblur. To the best of our knowledge, Deblur was not optimized for the processing of long reads, but we wished to investigate whether, along with DADA2, the ASV paradigm could be applied to this type of data. In that regard the recent implementation of DADA2 geared for PacBio Circular Consensus Sequence (CCS) reads [35] effectively managed to integrate more reads into ASVs in comparison with Deblur. However, Mantel correlations of both ASV methods (Deblur and DADA2) with OTUs (VSEARCH and DNACLUST) were fairly low for long 16S reads for the mock community. For the oral microbiome samples, OTU-based (VSEARCH and DNACLUST) communities were highly similar to DADA2’s ASVs but different from Deblur's ASVs (Table 3; weighted UniFrac). However, when comparing Bray-Curtis dissimilarity indexes, not factoring in phylogeny, microbial communities generated with OTUs were arguably different from the ones obtained by ASV methods (Table 3).

Compared to short reads, long PacBio amplicon data showed lower Mantel correlations among the 4 tested methods (Table 3). PacBio CCS reads have an accuracy of 99.999% according to the manufacturer's specifications. However, with long amplicons of around 1,400 bp, even such a high percentage of accuracy will eventually translate into actual errors, inevitably reflecting a high proportion of low-abundance sequences in OTU/ASV tables. This problematics is partially compensated for with clustering methods because reads having ≥97% identity will be merged in the same OTU. With the ASV paradigm, reads showing differences in a single base will form distinct ASVs, resulting in a more scattered abundance table, which inevitably affects indexes based on abundance such as the Bray-Curtis dissimilarity index.

Overall, the choice of distance/dissimilarity metric (weighted UniFrac or Bray-Curtis) had a remarkable impact on our most “distinctive” datasets: (i) highly diverse amplicon lengths for the ITS datasets, (ii) long sequences for the PacBio data, and (iii) simple bacterial population for the mock community data.

### OTUs and ASVs

OTU generation methods can be divided into 2 broad categories referred to as close-reference and *de novo* methods. Comparison between the 2 paradigms has been the subject of recent debates [36, 37], and we have focused our attention here on *de novo* methods for OTU generation (DNACLUST and VSEARCH). More recently, the adoption of ASVs as an alternative to OTUs has gained traction in the microbial ecology community, mainly to avoid the arbitrary dissimilarity clustering threshold inherent to *de novo* OTU generation methods. One reported advantage of ASV is the higher resolution that they provide compared to OTUs—because no signal is lost during the sequence clustering process. ASVs have been reportedly used to distinguish between bacteria at the species level, but such a practice of identifying short amplicons at the species and strain level is controversial because there is no uniformly accepted definition of bacterial species [38,39] or strain [38]. Moreover, even with PacBio long amplicon rRNA data (approximately 1,400 bp reads) we often cannot confidently assign taxonomy at the species level (personal observations and Fig. S6a). Therefore, inferring classification of short amplicons of a few hundred bases' data to up to the species level should be considered with extreme caution. Besides, our data show that, except for ITS and long PacBio (for the reasons explained above) 16S amplicons, ecological patterns (taxonomy, β- and α-diversity) were very similar between ASVs and OTUs in the 16S- and 18S-based studies we analysed, which is consistent with recent studies comparing ASVs and OTUs [ 40,41]. Another argument in favor of adopting ASVs is that they should allow different studies to be compared without the need to recompute OTUs [33]. However, re-usability across studies assumes that DNA of these different studies have been extracted using the same method [42,43] and DNA amplified using the same primer sequences. If sequencing libraries to be analysed meet these criteria, in practice, it is probably more opportune to pool all libraries together and re-initiate ASV generation to make sure that samples have been processed the same way (e.g., exact same parameters) in upstream steps of ASV/OTU generation. Given the constant improvement of compute hardware and efficiency of OTU clustering methods, re-generating OTUs or ASVs as new datasets for a given project become available can be considered a viable option: our results (Fig.   1—right panels) show that VSEARCH completed in ∼4.5 hours compared to 5.2 hours for Deblur for the indoor microbiome project data, but that VSEARCH significantly outperformed Deblur for the other datasets. Another aspect that we observed from our ASV tables is the “staircase” pattern typically observed in lower-abundance ASV, which is illustrated using the ASV table from the AAD project with 6 samples as an example (Table S4). From this table, we see that because ASVs are discerned at a single-base resolution, multiple ASVs pointing to the same taxonomic groups are generated. Probably because the sequencing errors are “corrected” or “absorbed” by large clusters during the clustering process, this staircase pattern is absent in OTU tables. There are situations where ASVs can be useful to achieve correlation between an amplicon sequence and its associated genome. In such a situation, an alignment of 100% identity between an ASV and a reference sequenced genome may be necessary to make such a correlation; because of its inherent nature, an OTU representative sequence would probably rarely achieve a perfect alignment against its associated reference genome. Regardless of the ASV or OTU generation method used, the use of short amplicon sequencing should mainly aim at offering a broad snapshot of the microbial communities at stake in a biological system. In all cases, the AmpliconTagger pipeline can be customized and accommodate any OTU or ASV generation method, being agnostic to the current debate.

In conclusion, microbial ecology is more than ever relying on high-throughput sequencing technologies. Bioinformatic pipelines used for analysing these data loads are increasing in complexity and there is a need for increased flexibility in the systematic analysis of short rRNA amplicon data. End-to-end pipelines do exist, but these solutions are not necessarily conducive to an easy integration of third-party packages or in-house software. Moreover, these pipelines, as they are provided, are mostly geared toward interactive or single-batch job processing and can generate inessential intermediate files, which can be constraining in a production context. AmpliconTagger is intended to provide a backbone for automated short amplicon data processing with an easy way for literate Python coders to add or remove jobs and steps and thus customize the pipeline to their specific needs or preferences.

## Potential Implications

High-throughput nucleic acid sequencing is entering public life and is becoming increasingly democratized. However, the bioinformatics analysis dimension that comes with nucleic acid sequencing projects is still often underestimated or poorly considered in the overall planning of sequencing data processing. Bioinformatic pipelines are complex with many different fine-tuned steps, and there is a need for flexibility for parametrization and customization. The objective of the present study was to provide an example of a fully functional automated pipeline to process a variety of rRNA amplicon sequencing data types. Short amplicon data size is inherently small compared to other high-throughput sequencing fields such as shotgun metagenomics or large eukaryote genome sequencing. This small data type was chosen specifically to illustrate the proof of concept of creating a highly customized marker gene pipeline offering bioinformaticians who operate them a suitable alternative to existing widespread solutions such as QIIME and Mothur. AmpliconTagger is integrated into the GenPipes workflow management system [21], and as such, it is easily customizable to adapt for specific needs and is practical for the integration of external bioinformatic packages. It allows the leveraging of compute job schedulers that are part of modern HPC environments and options tweaking and optimization. For instance, a clinical laboratory performing the monitoring of microbial communities could effectively add a step that computes source tracking [44]—to predict the source of microbial communities in a set of samples—for each pipeline run. A laboratory with research interests in non-conventional marker genes (e.g., *cpn60* and *rpoB*) or functional genes such as *phoD* and *pmoA* could also build their own reference database and training sets and promptly integrate them into the workflow.

## Methods

### Structure of the AmpliconTagger workflow

This is the main structure of the AmpliconTagger workflow. Specific parameters mentioned in this section reflect the ones that were used in this study but can be customized in accordance with the user's needs as described in the user guide (Additional File 4).

1. Reads are first scanned for contaminants (e.g., Illumina, 454, or PacBio adapter sequences) and PhiX reads using a Decontamination Using Kmers approach (bbduk, part of the bbmap software [45]). Usually, a small proportion of reads are contaminants and accordingly, 0–25% are PhiX reads.
2. Removal of unpaired reads. From Step 1, paired-end reads may be disrupted. This means that 1 of the read pairs might be lost owing to the screening in Step 1. All of these unpaired reads are discarded. This is usually a fairly small proportion of all reads. This step is not performed if reads are single ended (MiSeq single ended, PacBio, IonTorrent, or 454).
3. If reads are of single-end configuration (i.e., 454, IonTorrent, or PacBio data types), they are trimmed to a fixed length that is variable depending on the quality of sequencing run and amplicon length. If reads are paired end (Illumina), trimming can be optional and should be performed in such a way that enough bases are left on the 3′ end of each read pair to allow assembly using forward (read 1) and reverse (read 2) common overlapping parts during merging of read pairs in the next step. Nevertheless, in the case of paired-end reads, it is arguably preferable to trim the 3′ portion of reads that shows low quality before reads performing the overlapping paired assembly.
4. If paired-end reads: reads are assembled (overlapping paired assembly) using FLASH software (FLASH, RRID:SCR_005531) [46].
5. Primer sequences may or may not be removed from the assembled/single-end reads. Primer sequences should be removed when possible/applicable because the primer annealing regions of amplified DNA may be overrepresented in sequencing errors (personal observations). In the case of long PacBio reads, it has been observed that CCS reads can be generated in both forward and reverse orientation. In that case, reverse reads should be correctly oriented using primer sequence information.
6. The trimmed assembled/single-end reads from steps 4 and 5 are filtered for quality. All reads having an average quality score <27–33 or >0 or 1 N (undefined base) and 5 nucleotides below quality 15 are discarded. The remaining reads will be referred to as filtered reads from now on. Filtering parameters are customizable and should be adapted to the quality profile of each dataset.
7. Filtered reads are then clustered with our in-house clustering workflow. Briefly, reads are clustered at 100% identity and then clustered/denoised at 99% identity (VSEARCH, DNACLUST [29,30]) (DNACLUST, RRID:SCR_001771). Clusters having abundances <25 are discarded. The remaining clusters are then scanned for chimeras with VSEARCH's version of UCHIME denovo and UCHIME reference [29,47] (UCHIME, RRID:SCR_008057) and clustered at 97% (DNACLUST or VSEARCH) to form the final clusters/OTUs. In the case of Deblur and DADA2, ASVs having abundance <25 are discarded. For PacBio long amplicons, filtered reads are clustered at 97% identity and clusters having <2 reads (i.e., customizable parameter) are discarded. The remaining reads are scanned for chimeras using UCHIME reference. If Deblur or DADA2 are used, filtered reads are used as input for Deblur or DADA2 [31]. In the case of DADA2, input paired-end reads are provided as forward and reverse reads separately (not paired-end assembled). The resulting Deblur ASVs are then scanned for chimeras with VSEARCH's version of UCHIME denovo and UCHIME reference [29,47]. DADA2’s ASVs are filtered for chimeras using the package's internal algorithm (removeBimeraDenovo) followed by VSEARCH's UCHIME reference.
8. OTUs/ASVs are then assigned a taxonomic lineage with the RDP classifier [48] using an in-house training set containing the complete Silva release 128 database [49] supplemented with eukaryotic sequences from the Silva databases and a customized set of mitochondria, plastid, and bacterial 16S sequences. The ITS2 database consists of the UNITE ITS database (ITS1-ITS2) region. The 18S training set was built with the Silva eukaryote release 128 database. The RDP classifier gives a score (0–1) to each taxonomic depth of each OTU. Each taxonomic depth having a score ≥0.5 is kept to reconstruct the final lineage. OTUs/ASVs are also blasted against the most recent NCBI nt database for complementary information.
9. Using taxonomic lineages obtained from Step 8 combined with cluster abundance from Step 7, a raw OTU/ASV table is generated. From that raw OTU/ASV table, an OTU/ASV table containing both bacterial and archeal organisms is generated. From this latter OTU/ASV table, a normalized (edgeR, RRID:SCR_012802 [50,51]) and consensus-rarefied (as described in more detail below) OTU/ASV table is generated. If data consist of ITS amplicons, the same procedures are applied, but the raw OTU/ASV table is filtered to keep fungal organisms only. If the data are derived from 18S amplicons, the OTU/ASV table is filtered to keep eukaryotic organisms only. From this point on, the rarefied consensus OTU/ASV table is used for downstream analyses.
10. A summary of read counts throughout the different steps of the pipeline is generated. This is useful to get a global outlook on the sequencing run: how many reads were sequenced, how many reads were filtered out after QC, how many OTUs/ASVs were generated, etc.
11. From these classified OTUs/ASVs, a multiple sequence alignment is then obtained by aligning OTU/ASV sequences on a Greengenes core reference alignment [52] using the PyNAST aligner [17]. If data type is ITS or 18S, OTU/ASV sequences are aligned against a Unite or Silva eukaryote core alignment, respectively. For short amplicon data, alignments are filtered to keep only the hypervariable region of the alignment. For long PacBio reads, the whole alignment is kept.
12. A phylogenetic tree is then built from that alignment (from Step 11) with FastTree (FastTree, RRID:SCR_015501) [53]. The α (observed species) and β (weighted and unweighted UniFrac and Bray-Curtis distances) diversity metrics and taxonomic summaries are then computed using the QIIME 1 software suite (QIIME, RRID:SCR_008249) [17, 54]. Along with the OTU/ASV tables, these last tables represent end results from the pipeline and can then be used to generate various types of plots and statistics computation.

### Read clustering and ASV methodology

Our OTU generation procedure was implemented on the basis of a procedure previously described (Lundberg et al. 2012 [55]) and uses either DNACLUST or VSEARCH for the read clustering step. Briefly, quality controlled reads/sequences are dereplicated at 100% identity. The dereplication step is necessary to lower data load for the clustering software because only 1 representative of many thousands of identical sequences is kept for clustering. Counts of each unique sequence representative are kept in sequence headers after the dereplication process. For instance for the AAD study, the fastq file holding quality controlled paired-end assembled reads holds 9,209,510 sequences. Once dereplicated, these sequences are actually regrouped into 831,570 sequences, which represent a 11.1-fold data reduction. These dereplicated sequences are then clustered at 99% identity (DNACLUST or VSEARCH). Clusters having an abundance of <25 reads (e.g., customizable parameter) are then discarded and the remaining clusters are then scanned for chimeras with UCHIME denovo and UCHIME reference [47] and clustered again at 97% identity (DNACLUST or VSEARCH) to form the final clusters.

### RDP classifier training sets

The RDP classifier is a Bayesian classifier whose purpose is to classify sequences against a training set. Existing training sets are based on 99% identity clustered versions of either Greengenes or Silva databases. The RDP database (not to be confused with the RDP classifier software) was also built in a similar manner. To improve resolution of classification, we built our own custom training sets using the whole Silva SSU (release 128) database. We had to semi-automatically and manually alter the classification of certain taxa in order to make the lineages unique and non-conflicting. At the time of writing, we are using a training set based on the Silva 128 release. We also built our own training sets for 18S and ITS sequences. The taxonomic classification system for eukaryotic organisms is far more complex than that for the simpler bacterial kingdom. As such, additionally to the common kingdom, phylum, class, order, family, and genus fields found in prokaryotic taxonomy, eukaryotic taxonomy includes ranks such as subphylum, subdivision, subclass, superorder, suborder, and subfamily, which makes the task of generating consistent values for each rank challenging. Importantly, in order to obtain more resolution from taxonomic classifications, our training sets were generated using the entirety of the Silva and Unite databases and not the clustered or “OTU” versions (i.e., clustered at various identity thresholds ranging from 95 to 99%) of these databases. Perl code used to generate training sets and training sets themselves are available in the following repository: https://github.com/jtremblay/RDP-training-sets.

### Normalizing OTU/ASV tables with a multi-rarefaction procedure

Normalization of ASVs or OTUs is a controversial topic [50,56]. Until a durable solution gets accepted by the microbial ecology research community, we favor a multi-rarefaction approach as a means to generate a normalized OTU/ASV table. Briefly, the raw OTU table is first filtered for targeted microorganisms—if 16S primers were used, only OTUs/ASVs matching to Bacteria at the kingdom level will be kept for downstream steps. This filtered OTU/ASV table is then rarefied 500 times and the mean of each OTU/ASV of each sample is then computed so that a consensus rarefied table is obtained. Proceeding this way avoids the bias introduced by performing a single random rarefaction, which inevitably leaves out low-abundance micro-organisms. This consensus rarefied table is then used for downstream analyses (e.g., α-, β-diversity, taxonomic summaries).

### Smart restart mechanism using a workflow management system

Bioinformatics pipelines are intrinsically complex with many steps that need to be executed in a specific order. To improve productivity, pipelines should be executed on a compute cluster using a compute job scheduler (e.g., Torque, SLURM) supporting job dependencies. This way, the jobs of a complex pipeline can be submitted all at once to the job scheduler so that each job can be available for execution only when its depending job has successfully completed. For example, in a typical rRNA gene amplicon pipeline, the OTU/ASV generation job(s) can enter the waiting queue only when the QC job it depends on have all been successfully completed. Only then, the OTU/ASV generation job will enter the queue for execution. Many pipeline modules (software that generates scripts of job submissions) have been written and published [57]. A good pipeline framework should generate jobs, manage their dependencies, and have a smart restart mechanism in case of job failure. In the context of a complex pipeline with hundreds to thousands of jobs, a smart restart mechanism is indispensable to gain productivity and save time determining which job failed. The GenPipes core modules fill these requirements and is the reason for their adoption [21]. For instance if the execution of AmpliconTagger gets interrupted because of a job failure, it should be straightforward to identify exactly which job failed to properly execute. With a smart restart mechanism implementation, the pipeline framework should find, upon re-execution, which job actually failed to successfully complete and effectively rewrite it for re-submission. Bioinformatics pipeline frameworks are also critical in that they allow sequencing data to be systematically analysed in reproducible ways and that each step or job generated is parameterizable. For instance when analysing quality controlled read data results, one can realize that the quality-filtering parameters were too stringent given the quality score profiles of the input sequencing data. By slightly decreasing the quality-filtering parameters and re-running the pipeline framework, all the downstream jobs affected by this modified parameter will be re-generated and re-submitted to the job scheduler. Proceeding with a pipeline framework also leaves traces of parameters used in all jobs should the data and analyses be revisited in the future.

### Sequencing library preparation for mock community DNA

Mock communities purified DNA was purchased from BEI resources (Manassas, VA, USA) as HM-782D (even spike-in of total mock community). 16S rRNA gene amplicon libraries were prepared as described [58].

## Availability of Source Code and Requirements

Project Name: AmpliconTagger

Project Home Page: http://jtremblay.github.io/amplicontagger.html

Operating System: CentOS 7

Programming Languages: Python, Perl, R

Other requirements: pynast/1.2.2; perl/5.26.0; rdp_classifier/2.5; fasttree/2.1.10; FLASH/1.2.11; qiime/1.9.1; duk/1.051; DNACLUST/3; fastx/0.0.13.2; python/2.7.5; python/3.6.5; java/jdk1.8.0_144; blast/2.6.0+; Deblur/1.0.4; VSEARCH/2.7.1; R/3.6.0, DADA2/1.12.1

License: GNU GPL

The AmpliconTagger pipeline wrapper code and Python, Perl, and R scripts that are being called by AmpliconTagger are available here:

https://bitbucket.org/jtremblay514/nrc_pipeline_public/src/1.1/

https://bitbucket.org/jtremblay514/nrc_tools_public/src/1.1/

External software packages module install scripts are available here:

https://bitbucket.org/jtremblay514/nrc_resources_public/src/1.1/

A Docker image built on the CentOS 7 operational system that contains all necessary modules for full pipeline functionality is available for testing/evaluation purposes and running small datasets (https://cloud.docker.com/u/julio514/repository/docker/julio514/centos). Scripts used to generate RDP training sets are available here: https://github.com/jtremblay/RDP-training-sets, and the training set files are available on the Docker image. The PipelineViewer web page is located here: http://jtremblay.github.io/PipelineViewer/amplicontagger.html and its source code is available here: https://github.com/jtremblay/PipelineViewer.

## Availability of Supporting Data and Materials

Sequencing for the indoor microbiome project is available through the ENA portal under accession number ERP005806. 16S rRNA amplicon sequence data for the AAD study are available in the NCBI's SRA portal under accession number SRP120170. PacBio full-length 16S rRNA amplicons for the oral microbiome project are available under SRR56217[29–69]. ITS amplicons from the plant root microbiome transplant study are available under PRJNA301462. 18S rRNA gene amplicon data from the Chicago Lake Michigan study are available under PRJNA294919/SRP063479. The even mock community reads are available under PRJNA510326. The staggered mock community is available under SRR2082918–20 and the PacBio mock libraries under SRR559331[4–7], SRR55933[19]-[20], and SRR559333[2]-[3]. All raw data and intermediate files used and generated for this study are available in GigaDB [59]. All commands used to process all of the 6 datasets are also available in GigaDB [59].

## Additional Files

Additional File 1

Figure S1. Study design.

Figure S2. Comparison between Deblur, DADA2, DNAclust and VSEARCH for a) taxonomic profiles, b) beta diversity including Mantel test results between the three tested methods and c) alpha diversity of selected samples from the indoor microbiome project (16S V3-V4 region; 1x150 bp).

Figure S3. Comparison between Deblur, DADA2, DNAclust and VSEARCH for a) taxonomic profiles, b) beta diversity including Mantel test results between the three tested methods and c) alpha diversity of selected samples from the the antibiotic-associated diarrhea (AAD) project (16S V4 region; 2x250 bp).

Figure S4. Comparison between Deblur, DADA2, DNAclust and VSEARCH for a) taxonomic profiles, b) beta diversity including Mantel test results between the three tested methods and c) alpha diversity of selected samples from the rhizosphere transplant project (ITS1-ITS2 region; 2x250 bp).

Figure S5. Comparison between Deblur, DADA2, DNAclust and VSEARCH for a) taxonomic profiles, b) beta diversity including Mantel test results between the three tested methods and c) alpha diversity of selected samples from the Chicago nearshore water profiling project (18S region amplified by the 1181F-1624R primers; 1x151 bp).

Figure S6. Comparison between Deblur, DADA2, DNAclust and VSEARCH for a) taxonomic profiles at the species level, b) beta diversity including Mantel test results between the three tested methods and c) alpha diversity of selected samples from the PacBio mock community (16S, V1-V9 region; PacBio 1x~1470 bp CCS reads).

Figure S7. Comparison between Deblur, DADA2, DNAclust and VSEARCH for a) taxonomic profiles at the species level, b) beta diversity including Mantel test results between the three tested methods and c) alpha diversity of selected samples from the oral microbiome project (16S, V1-V9 region; PacBio 1x~1473 bp CCS reads).

Figure S8. Read length frequency distribution for ITS, 16S and 18S short reads data types.

Additional File 2. Complete commands traces of each job of each data analysis run.

Additional File 3. QIIME2 command traces for the 16S V4 region mock community.

Additional File 4. AmpliconTagger user guide.

Table S1. MiSeq V4 and PacBio even microbial mock community strains and their genome properties. Concentration values taken from the manufacturer. Microorganism Expected Normalized distribution (MEND) was computing following procedure described in Tremblay et al., 2015 (Front. Microbiol.).

Table S2. MiSeq V4 staggered microbial mock community strains and their genome properties. Taken from Tremblay et al., 2015 (Front. Microbiol.). MEND=Microorganism Expected Normalized distribution.

Table S3. Mantel *r* statistics comparing distance matrices of each ASVs/OTUs generation method for each project. Each *r* statistic had a p value < 0.001.

Table S4. Staircase pattern observed for six samples selected from the AAD ASV table.

giz146_GIGA-D-19-00228_Original_Submission

giz146_GIGA-D-19-00228_Revision_1

giz146_Response_to_Reviewer_Comments_Original_Submission

giz146_Reviewer_1_Report_Original_Submission Naga Betrapally -- 7/22/2019 Reviewed

giz146_Reviewer_1_Report_Revision_1 Naga Betrapally -- 9/24/2019 Reviewed

giz146_Supplemental_Files

## Abbreviations

AAD: antibiotic-associated diarrhea; ASV: amplicon sequence variant; bp: base pairs; CCS: Circular Consensus Sequence; Gb: gigabase pairs; GB: gigabytes; GUI: graphical user interface; HPC: high-performance computing; MB: megabytes; NCBI: National Center for Biotechnology Information: NIH: National Institutes of Health; OTU: operational taxonomic unit; PacBio: Pacific Biosciences; QC: quality control; RAM: random access memory; rRNA: ribosomal RNA; SRA: Sequence Read Archive.

## Competing Interests

The authors declare that they have no competing interests.

## Authors' Contributions

J.T. planned the experimental design, wrote the software, analysed the data, and wrote the manuscript. E.Y. edited the manuscript.
