# Supplementary material for: Systematic processing of ribosomal RNA gene amplicon sequencing data
Source: Gigascience. 2019 Dec 9;8(12):giz146. doi: 10.1093/gigascience/giz146 (PMC6901069; doi:10.1093/gigascience/giz146)
Supplement: giz146_GIGA-D-19-00228_Original_Submission [file giz146_giga-d-19-00228_original_submission.pdf]

GigaScience
Systematic processing of rRNA gene amplicon sequencing data
--Manuscript Draft--

**Manuscript Number:**	GIGA-D-19-00228
**Full Title:**	Systematic processing of rRNA gene amplicon sequencing data
**Article Type:**	Research
**Funding Information:**	
**Abstract:**	With the advent of high throughput sequencing, microbiology is increasingly becoming a data intensive field of science. Because of its low cost, robust databases and established bioinformatic workflows, sequencing of 16S/18S/ITS rRNA gene amplicons, which provides a marker of choice for phylogenetic studies, has become ubiquitous and has grown into the backbone of modern microbial ecology.<br><br>Many established end-to-end bioinformatic pipelines are available to perform short amplicon sequence data analysis and have proven to be central for advancing the field of microbial ecology. These pipelines have been partly written for a general audience, which is arguably a main reason for their widespread adoption. However, few options exist for more specialized users that are experienced in Linux-based systems and high performance computing (HPC) environments. For such an audience, existing pipelines can be limiting to fully leverage modern HPC capabilities and perform tweaking and optimization operations. Moreover, a wealth of stand-alone software packages that perform specific targeted bioinformatic tasks are increasingly accessible through code repositories and scientific publications and finding a way to easily integrate these applications in a pipeline is critical in fast-paced evolution of bioinformatic methodologies.<br><br>Here we describe AmpliconTagger, a short rRNA marker gene amplicon pipeline coded in a python framework that enables fine tuning and integration of virtually any potential rRNA gene amplicon bioinformatic procedure. It is designed to work within an HPC environment, supporting a complex network of job-dependencies with a smart-restart mechanism in case of job failure or parameter modifications.<br><br>As proof of concept, we present end results obtained with AmpliconTagger using 16S, 18S, ITS rRNA short gene amplicons and PacBio long read amplicon data types as input. Using a selection of published algorithms for generating Operational Taxonomic Units (OTUs) and Amplicon Sequence Variants (ASVs) and for computing downstream taxonomic summaries and diversity metrics, we demonstrate the performance and versatility of our pipeline for systematic analyses of amplicon sequence data.
**Corresponding Author:**	Julien Tremblay<br><br>CANADA
**Corresponding Author Secondary Information:**	
**Corresponding Author's Institution:**	
**Corresponding Author's Secondary Institution:**	
**First Author:**	Julien Tremblay
**First Author Secondary Information:**	
**Order of Authors:**	Julien Tremblay
	Etienne Yergeau
**Order of Authors Secondary Information:**	
**Additional Information:**	

Powered by Editorial Manager® and ProduXion Manager® from Aries Systems Corporation

Question	Response
Are you submitting this manuscript to a special series or article collection?	No
**Experimental design and statistics**<br><br>Full details of the experimental design and statistical methods used should be given in the Methods section, as detailed in our Minimum Standards Reporting Checklist. Information essential to interpreting the data presented should be made available in the figure legends.<br><br>Have you included all the information requested in your manuscript?	Yes
**Resources**<br><br>A description of all resources used, including antibodies, cell lines, animals and software tools, with enough information to allow them to be uniquely identified, should be included in the Methods section. Authors are strongly encouraged to cite Research Resource Identifiers (RRIDs) for antibodies, model organisms and tools, where possible.<br><br>Have you included the information requested as detailed in our Minimum Standards Reporting Checklist?	Yes
**Availability of data and materials**<br><br>All datasets and code on which the conclusions of the paper rely must be either included in your submission or deposited in publicly available repositories (where available and ethically appropriate), referencing such data using a unique identifier in the references and in the "Availability of Data and Materials" section of your manuscript.	Yes

Have you have met the above requirement as detailed in our Minimum Standards Reporting Checklist?	

Manuscript

Click here to download Manuscript AmpliconTagger_paper_rev3_2019-06-19.docx

Systematic processing of rRNA gene amplicon sequencing data.

Julien Tremblay[1#] and Etienne Yergeau[2]

[1]: Energy Mining and Environment, National Research Council Canada, Montreal, QC, Canada H4P-2R2

[2]: Centre INRS-Institut Armand-Frappier, Institut national de la recherche scientifique, 531 Boul. des Prairies, Laval, QC, Canada, H7V-1B7

[#]: Corresponding author - julien.tremblay@nrc-cnrc.gc.ca

Running title: AmpliconTagger pipeline

Abstract

With the advent of high throughput sequencing, microbiology is increasingly becoming a data intensive field of science. Because of its low cost, robust databases and established bioinformatic workflows, sequencing of 16S/18S/ITS rRNA gene amplicons, which provides a marker of choice for phylogenetic studies, has become ubiquitous and has grown into the backbone of modern microbial ecology.

Many established end-to-end bioinformatic pipelines are available to perform short amplicon sequence data analysis and have proven to be central for advancing the field of microbial ecology. These pipelines have been partly written for a general audience, which is arguably a main reason for their widespread adoption. However, few options exist for more specialized users that are experienced in Linux-based systems and high performance computing (HPC) environments. For such an audience, existing pipelines can be limiting to fully leverage modern HPC capabilities and perform tweaking and optimization operations. Moreover, a wealth of stand-alone software packages that perform specific targeted bioinformatic tasks are increasingly accessible through code repositories and scientific publications and finding a way to easily integrate these applications in a pipeline is critical in fast-paced evolution of bioinformatic methodologies.

Here we describe AmpliconTagger, a short rRNA marker gene amplicon pipeline coded in a python framework that enables fine tuning and integration of virtually any potential

rRNA gene amplicon bioinformatic procedure. It is designed to work within an HPC environment, supporting a complex network of job-dependencies with a smart-restart mechanism in case of job failure or parameter modifications.

As proof of concept, we present end results obtained with AmpliconTagger using 16S, 18S, ITS rRNA short gene amplicons and PacBio long read amplicon data types as input. Using a selection of published algorithms for generating Operational Taxonomic Units (OTUs) and Amplicon Sequence Variants (ASVs) and for computing downstream taxonomic summaries and diversity metrics, we demonstrate the performance and versatility of our pipeline for systematic analyses of amplicon sequence data.

Background

High throughput sequencing of amplicons of fragments of the 16S, 18S and ITS rRNA marker genes has grown into a cornerstone of microbial ecology research activities. Amplicon sequencing is now massively widespread and has been used in large research initiative such as the NIH funded Human Microbiome Project[1–3] and Earth Microbiome Project[4].

Despite this, it is still objectively difficult to adequately analyze data[5,6]. Initiatives to provide graphical user interface-based applications have been reported[7–9]. These types of interfaces, by their fundamental nature, are not prone for systematic analysis in a production context involving the processing of high data loads of multiple projects

simultaneously.

Efforts to integrate bioinformatic pipelines as a standard tool to establish microbiome profiles in food safety and energy settings are increasingly being reported[10,11] and 16S rRNA marker genes studies are increasingly being reported to be relevant to complement traditional methods in a clinical context[12–16].

The bioinformatic landscape for processing short marker gene amplicon sequencing data contains a wide array of solutions and is dominated by a few open-source popular pipelines such as Qiime[17] and Mothur[18]. In order to execute, these pipelines usually require users to use streamlined or pre-defined steps with limited ability for advanced customization. For instance Qiime2[19], allows to use dada2[20] for ASV generation, but only a few input parameters are accessible to the user compared to much more from the dada2 original R package. These efforts were and are continuing to be immensely important in democratizing rRNA amplicon data processing, making it possible for uninitiated Linux users to be able to perform their own data analysis. As such, these pipelines are arguably targeting investigators unfamiliar with computer coding and command line execution. This kind of enclosed setting, however, can become a limiting factor for the types of users who are both proficient at code scripting and comfortable in a Linux/command-line environment. Moreover, bioinformatic methods are constantly evolving so that production bioinformatic pipelines need to be adapted and modified on a regular basis to properly integrate newly published bioinformatic packages.

Bioinformatic pipelines are intrinsically complex with up to hundreds of steps depending on the input sequencing data type with some of these steps needing large compute resources to properly execute. It is critical to introduce robust and flexible ways of systematically processing metagenomic sequencing data types (*i.e.* mainly amplicons and shotgun) in order to increase their adoption in the aforementioned settings. The GenPipes workflow management system, including an implementation of a Qiime-based 16S rRNA amplicon pipeline, was recently published[21]. Here, as a proof of concept, we leveraged GenPipes's capabilities to build AmpliconTagger, a versatile bioinformatic pipeline managing job generation, submission, dependency and smart restart that can process any type of gene amplicon sequencing data (16S, 18S, ITS rRNA genes and other custom marker or functional genes) of various sequencing configurations integrating multiple bioinformatic packages. We validated our pipeline with three Qiime2 workflows (vsearch, deblur and dada2) using two mock communities datasets for which we know the exact community composition. We then present microbiome profiling results from published short (MiSeq) and long (PacBio) amplicons sequencing datasets using two Operational Taxonomic Units (OTUs) and one Amplicon Sequence Variants (ASVs) algorithms. We also present detailed information on our methodology so that it can be promptly used, adapted and improved by others.

Data Description

In order to document various aspects of AmpliconTagger, we processed seven published and publicly available datasets of rRNA amplicon sequencing data of various

sequencing configuration, targeting various marker genes and regions (Table 1). We aimed to include datasets from a variety of ecosystems: indoor, human gut and oral, soil and water. We also included a novel dataset consisting of a commercial mock community.

Analyses

Experimental design

Each of the datasets described in table 1 were processed into the AmpliconTagger pipeline which contains from 91 to 94 jobs depending on the OTU/ASV generation algorithm used and on sequencing configuration (paired vs single end sequencing data). All datasets were processed following a common core of quality filtering procedures, but submitted to two different methods of OTUs generation (Additional file 1 - Fig. S1) (Vsearch[29] and Dnaclust[30]) and one ASVs method (Deblur[31]). In addition, short amplicons mock community datasets were also entirely processed in Qiime2 using a vsearch, deblur and dada2 workflow, in order to compare our pipeline against a third-party reference method. We also present and discuss community profiling results of long PacBio amplicons using a mock community and a published oral microbiome sequencing dataset (Table 1). More in-depth analyses of common microbial ecology metrics were assessed for each project for each OTU/ASV generation method and are available in Additional file 1. Although we do present some high level analysis of ASVs vs OTUs end results, this study primarily aims at demonstrating the modularity and methodology implemented in AmpliconTagger and less at performing an exhaustive

comparison of the OTU/ASV-generating packages that we used. The complete data processing description of AmpliconTagger is described in the methods section and the complete commands of each job of each data analysis run is available in additional file 2 (AmpliconTagger command traces) and 3 (Qiime2 command traces for the 16S V4 region mock community). An exhaustive user guide is available in additional file 4.

Validation with mock communities and comparison with third party reference pipeline.

In order to validate AmpliconTagger, we processed two defined mock communities, one with even concentrations of 20 bacterial strains (Table S1) and one with staggered concentrations of 9 genomes (Table S2). We obtained community profiling results of each mock sample using AmpliconTagger (vsearch, dnaclust and deblur) and compared them with the end results of the same sequencing libraries, but entirely processed with Qiime2 using vsearch, deblur and dada2 workflows. Taxonomic profiles are highly similar across all tested methods (Fig. 1a and 1b) and minor differences are probably caused by the two different Silva R128 training sets used by AmpliconTagger (100% identity sequences) vs Qiime2 (database, clustered at 99% identity). Notably, Qiime2-dada2 overestimated the abundance of *Staphyloccocus* in one of the even mock samples (Fig. 1a) and all Qiime2 workflows identified major taxa as being assigned to *Clostridiales*;Other and *Enterobacteriales*;Others while AmpliconTagger classified them instead as *Lachnoclostridium* and *Pantoea* (Fig. 1b). Regardless of the methods used, all samples clustered similarly in beta-diversity ordinations (Fig. 1c) and show relatively similar alpha diversity values (Fig. 1d).

Performance

We compiled compute resources consumed for each of the datasets considered for this study (Fig. 2). The common core of AmpliconTagger consists of steps 1 to 6 as detailed below in the methods section. The indoor microbiome, Lake Michigan and AAD studies consumed similar amounts of core•hours (22.7, 20.2 and 26.6 core•hours respectively) while the mock community, oral microbiome and rhizosphere microbiome transplant studies took significantly less resources with respective values of 0.53, 2.2 and 5.2 core•hours. In terms of real time, the indoor microbiome data took the longest (8.8 hrs) followed by AAD (6.0), lake Michigan (4.8) and plant microbiome transplant. OTU/ASV generation jobs consumed the most resources for the indoor microbiome data which again is the dataset containing both the highest number of reads and base-pairs. A similar trend is observed with the long PacBio reads from the oral microbiome project where downstream steps consumed more resources than the common core steps. Dnaclust notably consumed lots of compute time to complete the clustering of PacBio (oral microbiome) reads. In contrast, common core steps consumed similar core•hours and more real time compared to downstream steps for the lake Michigan and AAD datasets.

Microbial ecology metrics obtained for each dataset.

Globally, ecological patterns were similar for the three tested methods for all projects (Table 3 and Additional file 1), except for the oral microbiome (16S PacBio) and plant microbiome transplant (ITS). The numbers of ASVs and OTUs obtained for a given project were fairly similar for short amplicon data types, except for the indoor

microbiome data which yielded 27,833 ASVs and 18,338 dnaclust OTUs and 14,334 vsearch OTUs (Table 2). In contrast, the number of deblur ASVs obtained with the oral microbiome data went from 757 to up to 114,783, depending of deblur's `-min-reads` parameter, compared to 10,022 and 5,180 for dnaclust and vsearch OTUs respectively. The total number of reads included in these OTU/ASV was of 18,083, 242,071, 353,676 and 385,900 for deblur `-min-reads 3`, deblur `-min-reads 1`, dnaclust and vsearch respectively. In general the amounts of generated ASVs/OTUs were similar, but ASVs represented less total reads.

In order to highlight differences between the three tested OTU/ASV generation methods, we compared their taxonomic summaries, alpha- and beta- diversities. These results are included in figures S2 to S7 (Additional file 1) and for clarity and practicality purposes, we narrowed sample selection for each project to a subset of experimental variables. We also computed the Mantel *r* statistic to assess for correlation between weighted UniFrac and Bray-Curtis distance matrices between all three tested methods (Table 3). Overall, and consistent with results reported in additional file 1, these results show that weighted UniFrac and Bray-Curtis distances/dissimilarities obtained with all three tested methods are quite similar with *r* statistic values greater than 0.9 for the indoor microbiome, AAD and Lake Michigan data types. The ITS data (rhizosphere transplant) showed low *r* statistics between the weighted UniFrac matrices (deblur vs dnaclust = 0.258; deblur vs vsearch = 0.570; dnaclust vs vsearch = 0.414). However, Mantel tests between Bray-Curtis dissimilarity matrices of all three OTU/ASV ITS data types gave *r* statistics of 0.903 (deblur vs dnaclust), 0.893 (deblur vs vsearch) and

0.991 (dnaclust vs vsearch). Weighted UniFrac distances of all three methods were similar for the Mock community with *r* statistics values all greater than 0.9. However, Bray-Curtis dissimilarity matrices were more divergent with *r* statistics values of 0.790 (deblur dnaclust), 0.221 (deblur vs vsearch) and 0.400 (dnaclust vs vsearch). Overall, this indicates that the type of distance metric used had a great impact on the microbial population structure assessment.

Validation of procedures with long PacBio reads datatype.

Using PacBio full length amplicon sequencing for microbial communities profiling is increasingly making inroads to complement population characterization based on short amplicon sequencing, but bioinformatics procedures are objectively recent and not as mainstream as they are with short length amplicon sequencing data. We therefore validated our long 16S reads processing methodology with a PacBio mock community library [27] (Table S1). Taxonomic profiles of all three tested methods (vsearch, dnaclust and deblur) are generally similar with some minor differences in low abundant taxa. However, more important discrepancies are actually observed between expected taxa and what was obtained with the actual sequencing libraries (Fig. S6a). Alpha diversity metrics were computed and observed ASVs reached nearly 1,000 whereas observed OTUs were much closer to the expected value of 23 (Fig. S6b). Mantel *r* statistics between dnaclust and vsearch were relatively high (0.851 weighted UniFrac and 0.844 for Bray-Curtis), but not computable for ASV data. For the PacBio (oral microbiome) data, Mantel *r* statistics computed between the UniFrac distance matrices of all three data types (Table 3) showed values of 0.721 (deblur vs dnaclust), 0.744 (deblur vs vsearch) and 0.777 (dnaclust vs vsearch), indicating relatively high similarity

between all ASVs and OTUs distance matrices when phylogenetic distance is factored in the distance computation. Bray-Curtis matrices, however, showed lower correlations between deblur vs dnaclust (0.268) and deblur vs vsearch (0.213), while dnacust vs vsearch (0.928) distances were highly correlated.

Discussion

Backbone for rRNA sequence processing methodology.

The primary objective of this report was to showcase AmpliconTagger, a highly modular HPC oriented pipeline geared for performing bioinformatic analyses of rRNA amplicon data. To facilitate implementation and testing, we shared a docker repository of a CentOS-7 image with a fully working implementation of AmpliconTagger which includes data of 20 sequencing libraries from the AAD study, all the databases, training sets and fully detailed commands of each job of each step of our pipeline. Our workflow also relies on a set of perl scripts and libraries labeled nrc_tools for which the complete code is also available in a code repository. We wish to emphasize that we do not present our methodology as a gold standard, but rather as a blueprint of an end-to-end open source working modular pipeline that is able to incorporate virtually any in-house scripts or third-party packages. We also recognize that some steps in our workflow could be improved and even be replaced by other potentially more efficient software or method, which is highly facilitated by the open modular nature of the pipeline.

Resources and time consumption.

With bioinformatics entering many spheres of research and service fields, there is a need for flexible, scalable and robust methods to systematically analyze high-throughput nucleic acid sequencing data of all types. Here we presented in details our gene amplicons production workflow with performance metrics and actual results from published rRNA marker gene datasets. We executed our workflow using two OTU generation methods - vsearch and dnaclust and one ASV generation method - deblur. We included vsearch because it represents the open source version of the popular usearch closed-source software. As such, vsearch is increasingly being used by the research community as a suitable replacement for usearch. Our group started to use dnaclust as a suitable open-source alternative to replace usearch some years ago and since then, we have processed many rRNA marker gene sequencing datasets using this package and it is the reason for its inclusion in this study. The goal of the present study was not necessarily to benchmark these three OTU/ASV generation methods, but rather to show the modular nature of the pipeline and offer useful metrics of what microbiome profiling results and resource consumption can be expected from using these software in the context of our whole workflow methodology.

While short amplicon sequence data processing is not that demanding from a computational resource point of view, a complete working pipeline requires many steps in order to get from the raw data to key end-results. Here we show that quality control and fastq preprocessing steps of sequencing data prior to OTU/ASV generation consumed the most core•hours for datasets that had an appreciable number of reads (indoor microbiome, Lake Michigan and AAD). The rhizosphere transplant project had

comparable number of reads, but possibly because of short ITS read length (once paired-end assembled), jobs relying on Qscore metrics like quality filtering and quality score profile compilation quickly completed (Fig. 2). For downstream steps, which correspond to the OTU/ASV generation to up to the pipeline completion, the indoor microbiome (single-end 150 bp reads) and oral microbiome (single-end 1400 bp reads) consumed the most resources, the former, because of the high raw data load (16.8 Gb) and the latter because of its long read lengths. In terms of memory consumption, the indoor microbiome project, again because of its high amount of raw bp, consumed significantly more RAM than the other projects.

Validation with mock communities.

Mock community profiling results obtained by our pipeline with the ones obtained with a qiime2 –vsearch -deblur and -dada2 workflow were highly concordant (Fig. 1). For the even mock community (Fig. 1b), the main difference resided in the high abundance of *Staphylococcus* in one of the four replicates of the qiime2-dada workflow results. Otherwise all results deriving from either AmpliconTagger or Qiime2 were essentially similar and consistent with the expected taxonomy. The staggered community (Fig. 1b) gave almost identical community structure patterns, but slight differences in taxonomic assignments were observed for the most abundant taxon which was supposed to be a *Lachnoclostridium* (Fig. 1b - expected panel), but ended up being classified as a *Lachnocolstridium 10* by AmpliconTagger (dnaclust, vsearch and deblur) and an undefined genus belonging to the *Clostridiales* order for the Qiime2 based workflows. A similar situation is observed with Qiime2 assigning a major taxa to an undefined genus

belonging to the *Enterobacteriales* order, while AmpliconTagger assigned it to the Escherichia-Shigella genus (Fig. 2b). Even though the two training sets used to classify OTUs/ASVs are built from the Silva 128 release, the Qiime2 training set was trained on a 99% identity clustered database and the AmpliconTagger training set was trained on a 100% identity (*i.e.* unclustered) fasta database, which probably explains the differences observed in taxonomy, which, all things considered, are quite minimal.

Robustness of ecological patterns between OTU and ASV based results.

One of the key advantage of the pipeline introduced here is that it is modular and can be customized to fit the research need of the user. For instance, we have shown here that both ASV and OTU generation methods can be used interchangeably, making the Amplicon Tagger pipeline agnostic to the current heated debate about ASV vs. OTU. ASV generation is a method that has recently gained traction[32] and is increasingly being adopted as an alternative to OTU-based analyses. Here we processed seven types of datasets and compared end results of OTU clustering procedures done with dnaclust, vsearch and one ASV based method (deblur). In most cases, results given by deblur are essentially identical to what is obtained by vsearch and dnaclust. One notable exception is for the ITS data type where the weighted UniFrac Mantel correlations between the three methods are significantly lower than what is observed for short 16S and 18S amplicon data types. This discrepancy might be due to the fact that ITS amplicon sequence lengths are quite variable as shown in figure S8 (Additional file 1), with significant amounts of reads dispersed from 200 to 350 bp. During the clustering process, many of the shorter reads are “absorbed” by the larger reads. For ASVs, all

these reads of various lengths eventually form a distinct ASV and in consequence many ASVs do not reach the cutoff of 25 reads (`--min-reads 25` argument in deblur). This holds true for all projects, were the number of ASV reads is always lower than the number of OTU reads (table 2), but is probably exacerbated in the ITS data were much of the amplicon sequences are of different lengths (Additional file 1 - Fig. S8). That said, one would still have expected the weighted UniFrac mantel correlation to be higher between dnaclust and vsearch OTUs - as observed in other data types - which was not the case. This could be explained by the way clusters are constructed in dnaclust and vsearch. We hypothesized that in the case of such a scattered profile of amplicon read lengths, it might be wise to avoid potential clustering artifacts and favor using ASVs with a lenient abundance cutoff (i.e. `--min-reads <int>` parameter for deblur). We therefore ran the pipeline again using minimal ASVs or OTUs abundance of 5 (data not shown), but once again the Mantel *r* statistics were still weak (deblur vs dnaclust: 0.467, deblur vs vsearch: 0.367 and dnaclust vs vsearch: 0.549). Using an abundance of cutoff of 0 did not improve either (deblur vs dnaclust: 0.233, deblur vs vsearch: 0.647 and dnaclust vs vsearch: 0.535). In contrast to UniFrac metrics comparisons, Bray-Curtis dissimilarity matrices were highly concordant between deblur, vsearch and dnaclust for the ITS data type. This implies that global alignments, inherent to generating the Unifrac distance matrix, may be impractical because of the high variability in sequence and length of ITS amplicons, as previously suggested [33].

PacBio long reads

PacBio taxonomic profiles generated by AmpliconTagger were generally consistent with the expected taxonomy (Fig. S6), which validates our pipeline's procedures for processing long high-quality 16S rRNA gene reads. However, this type of amplicon data also showed lower correlations between the three tested methods (Table 3). PacBio Circular Consensus Sequence (CCS) reads have an accuracy of 99.999% according to the manufacturer's specifications. However, with long amplicons of 1,400 bp, even such a high percentage of accuracy will eventually translate into actual errors, inevitably reflecting a high proportion of singlet sequences in OTU/ASV tables. To the best of our knowledge, deblur was not optimized for the processing of long reads, but we wished to investigate if the ASV paradigm could be applied to this type of data. We applied deblur's default parameters except for the abundance cutoff parameters (--min-size <int>). We tested two abundance cutoffs (deblur arguments: --min-reads 3 --min-size 1) such that all ASVs having a cumulative abundance lower than 3 reads across all samples are discarded. The same cutoff was applied for vsearch and dnaclust. However, because the clustering process of OTU generation "absorbs" singlets and other lower abundant reads into larger clusters, far less PacBio OTUs ended up being rejected compared to ASVs (Table 2) which greatly affected the resulting diversity metrics of PacBio 16S ASVs. This is especially reflected in the low correlations between the ASVs vs OTUs Bray-Curtis dissimilarity matrices: 0.416 for deblur vs dnaclust and 0.361 for deblur vs vsearch, but not between dnaclust vs vsearch (OTU vs OTU) (0.928). Lowering the --min-reads parameter to 1 helped in increasing the number of ASVs (Table 2) to more effective values. This issue is also exposed in the PacBio mock community were the quasi totality of the reads were

actually singlets. In that case, the deblur --min-reads parameter had to be set to 1 in order to not reject more 99.9% of the reads. This also has the consequence that nearly all reads in the resulting ASV table are also singlets, which makes downstream beta- and alpha- diversity metrics spurious. We tested deblur with more lenient parameters (–-mean-error <float> --indel-prob <float>), but it only marginally decreased the number of singlet ASVs (data not shown). While taxonomic profiles obtained with ASVs for the PacBio mock community are generally consistent with what is obtained with OTUs, there seems to be a low but consistent proportion of ASVs that are assigned closely related species in addition to the expected targets. For instance, the mock community contained two species belonging to the *Staphylococcus* genus (Fig. S6a): *Staphylococcus aureus* and *Staphylococcus epidermidis*. These two species have been identified in the OTU data, but the ASVs were assigned more closely related species such as *Staphylococcus saccharolyticus*, an uncultured *Staphylococcus* and undefined *Staphylococcus* species (*i.e.* Other). This situation is also observed with OTUs, especially with dnaclust OTUs, but is less exacerbated. More generally, our results highlight the challenge of properly assigning species taxa to long 16S reads using general databases, in our case the Silva DB. Regardless of the nature of the data (ASVs or OTUs), taxonomic assignment of PacBio CCS were accurate for the majority of the species of the mock community (Fig. S6a - *Actinomyces*, *Bacteroides*, *Clostridium*, *Deinococcus*, *Enterococcus*, *Helicobacter*, *Listeria*, *Neisseria*, *Propionibacterium*, *Pseudomonas* and *Streptococcus* panels), but seemed to be more challenging for *Escherichia coli*, *Bacillus cereus*, *Streptococcus mutans* and *Staphylococcus epidermidis*. For instance, we expected to identify the *Bacillus cereus*

species, but instead observed the *Bacillus Antharaxis* (vsearch) and an undefined bacillus (Other) (dnaclust) or all three of them in ASVs (deblur). This suggests that in some instances, PacBio CCS contains at least enough errors to cause misclassification at the species level when processed as ASVs and that clustering in the objective of correcting these errors also result in misclassification.

Similarly to what was observed for the PacBio oral microbiome data, the mock community distances highly correlated in weighted UniFrac metrics comparisons, but not when comparing Bray-Curtis dissimilarities. In both cases (oral microbiome and mock community), it seems that factoring in phylogeny into distance computation (weighted UniFrac) attenuated the differences between the OTU/ASV generation methods. Overall, the choice of distance/dissimilarity metric (weighted UniFrac or Bray-Curtis) had a remarkable impact on our most “distinctive” datasets: 1) highly diverse amplicon lengths for the ITS datasets, 2) long sequences for the PacBio data and 3) simple bacterial population for the mock community data.

OTUs and ASVs

OTU generation methods can be divided into two broad categories referred to as close-reference and denovo methods. Comparison between the two paradigms have been the subject of recent debates[34,35] and we have focused our attention here on *denovo* methods for OTU generation (dnaclust and vsearch). More recently, the adoption of ASVs as an alternative to OTUs has gained traction in the microbial ecology community, mainly to avoid the arbitrary dissimilarity clustering threshold inherent to denovo OTU

generation methods. One reported advantage of ASV is the higher resolution that they provide compared to OTUs - because no signal is lost during the sequence clustering process. ASVs have been reportedly used to distinguish between bacteria at the species level, but such a practice of identifying short amplicons at the species and strain level is controversial as there is no uniformly accepted definition of bacterial species[36,37] or strain[36]. Moreover, even with PacBio long amplicon rRNA data (1,400 bp reads) we often cannot confidently assign taxonomy at the species level using a defined mock community (personal observations and figure S6). Therefore, inferring classification of short amplicons of a few hundred bases data to up to the species level should be considered with extreme caution. Besides, our data show that, except for ITS and long PacBio (for the reasons explained above) 16S amplicons, ecological patterns (taxonomy, beta- and alpha- diversity) were very similar between ASVs and OTUs in the 16S and 18S based-studies we analyzed which is consistent with recent studies comparing ASVs and OTUs[38,39]. Another argument in favor of adopting ASVs is that they should allow to compare different studies without the need to recompute OTUs[32]. However, reusability across studies assumes that DNA of these different studies have been extracted using the same method[40,41] and DNA amplified using the same primer sequences. If sequencing libraries to be analyzed meet these criteria, in practice, it is probably more opportune to pool all libraries together and re-initiate ASV generation to make sure that samples have been processed the same way (e.g. same parameters) in upstream steps of ASVs/OTUs generation. Given the constant improvement of compute hardware and efficiency of OTU clustering methods, re-generating OTUs or ASVs as new datasets for a given project gets available is certainly a viable option: our

results (Fig. 1 - right panels) show that vsearch completed in about 4.5 hours compared to 5.2 hours for deblur for the indoor microbiome project data, but that vsearch significantly outperformed deblur for the other datasets. Another aspect that we observed from our ASV tables is the “staircase” pattern typically observed in lower abundant ASV, which is illustrated using the ASV table from the AAD project with six samples as an example (Additional file 1 and table S3). From this table, we see that because ASVs are discerned at a single base resolution, multiple ASVs pointing to the same taxonomic groups are generated. Probably, because the sequencing errors are “corrected” or “absorbed” by large clusters during the clustering process, this staircase pattern is absent in OTU tables. There are situations were ASVs can be useful to achieve correlation between an amplicon sequence and its associated genome. In such a situation, an alignment of 100% identity between an ASV and a reference sequenced genome may be necessary to make such a correlation, because of its inherent nature, an OTU representative sequence would probably rarely achieve a perfect alignment against its associated reference genome. Regardless of the ASVs or OTUs generation method used, the use of short amplicon sequencing should mainly aim at offering a broad snapshot of the microbial communities at stakes in a biological system. In all cases, the Amplicon Tagger pipeline can be customized and accommodate any OTU or ASV generation method, being agnostic to the current debate.

In conclusion, microbial ecology is more than ever relying on high-throughput sequencing technologies. Bioinformatic pipelines used for analyzing these data loads are increasing in complexity and there is a need for increased flexibility in the

systematic analysis of short rRNA amplicon data. End-to-end pipelines do exist, but these solutions are not necessarily conducive to an easy integration of third party packages or in-house software. Moreover, these pipelines, as they are provided, are mostly geared toward interactive or single batch job processing and can generate inessential intermediate files, which can be constraining in a production context. AmpliconTagger is intended to provide a backbone for automated short amplicon data processing with an easy way for literate Python coders for adding or removing jobs and steps and thus customize the pipeline to their specific needs or preferences.

Potential implications

High throughput nucleic acid sequencing is entering public life and is getting increasingly democratized. However, the bioinformatics analysis dimension that comes with nucleic acid sequencing projects is still often underestimated or poorly considered in the overall planning of sequencing data processing. Bioinformatic pipelines are complex with many different fine-tuned steps and there is a need for flexibility for parametrization and customization. The objective of the present study was to provide an example of a fully functional automated pipeline to process a variety of rRNA amplicon sequencing data types. Short amplicon data size is inherently small compared to other high throughput sequencing fields such as shotgun metagenomics or large eukaryotes genome sequencing. This small data type was chosen specifically to illustrate the proof of concept of creating a highly customized marker gene pipeline offering bioinformaticians who operate them a suitable alternative to existing widespread solutions such as Qiime and Mothur. AmpliconTagger is integrated into the GenPipes

workflow management system[42] and as such, it is easily customizable to adapt for specific needs and is practical for the integration of external bioinformatic packages. It allows leveraging compute job schedulers that are part of modern HPC environments and options tweaking and optimization. For instance, a clinical laboratory performing the monitoring of microbial communities could effectively add a step that compute source tracking[43] - to predict the source of microbial communities in a set of samples - for each pipeline run. A laboratory with research interests in non-conventional marker genes (e.g. *cpn60* and *rpoB*) or functional genes such as *phoD* and *pmoA* could also build their own reference database and training sets and promptly integrate them into the workflow.

Methods

Structure of the AmpliconTagger workflow

This is the main structure of the AmpliconTagger workflow. Specific parameters mentioned in this section reflect the ones that were used in this study, but can be customized as per the user's needs as described in the user guide (Additional file 4).

1. Reads are first scanned for contaminants (*e.g.* Illumina, 454 or PacBio adapter sequences) and PhiX reads using a Decontamination Using Kmers approach (bbduk, part of the bbmap software (unpublished - http://sourceforge.net/projects/bbmap/). Usually, a small proportion of reads are contaminants and accordingly, 0-25% are PhiX reads.
2. Removal of unpaired reads. From step 1, paired-end reads may be disrupted.

This means that one of the read pairs might be lost due to the screening in step #1. All of these unpaired reads are discarded. This is usually a fairly small proportion of all reads. This step is not performed if reads are single ended (MiSeq single ended, PacBio, IonTorrent or 454).

3. If reads are of single-end configuration (*i.e.* 454, IonTorrent or PacBio data types), they are trimmed to a fixed length that is variable depending on the quality of sequencing run and amplicon length. If reads are paired end (Illumina), trimming can be optional and should be done in such a way that enough bases are left on the 3' end of each read pair to allow assembly using forward (reads 1) and reverse (reads 2) common overlapping parts during merging of read pairs in the next step.
4. if paired-end reads : Reads are assembled (overlapping paired assembly) with the FLASH software[44].
5. Primer sequences may or may not be removed from the assembled/single end reads. Primer sequences should be removed when possible/applicable as the primer annealing regions of amplified DNA may be overrepresented in sequencing errors (personal observations).
6. The trimmed assembled/single-end reads from steps #4-5 are filtered for quality. All reads having an average quality score lower than 33 or more than 1 N (undefined base) and 5 nucleotides below quality 15 are discarded. The remaining reads will be referred to as filtered reads from now on.
7. Filtered reads are then clustered with our in-house clustering workflow. Briefly, reads are clustered at 100% identity and then clustered/denoised at 99% identity

(vsearch, dnaclust -[29,30]). Clusters having abundances lower than 3 are discarded. Remaining clusters are then scanned for chimeras with vsearch's version of UCHIME denovo and UCHIME reference[29,45] and clustered at 97% (dnaclust or vsearch) to form the final clusters/OTUs. For PacBio long amplicons, filtered reads are clustered at 97% identity and clusters having less than 2 reads (*i.e.* customizable parameter) are discarded. Remaining reads are scanned for chimera using UCHIME reference. If deblur is used, filtered reads are used as input for deblur[31]. Resulting ASVs are then scanned for chimeras with vsearch's version of UCHIME denovo and UCHIME reference[29,45]

8. OTU/ASV are then assigned a taxonomy. Briefly, OTU/ASV are classified with the RDP classifier[46] using an in-house training set containing the complete Silva release 128 database[47] supplemented with eukaryotic sequences from the Silva databases and a customized set of mitochondria, plastid and bacterial 16S sequences. ITS2 database consist of the UNITE ITS database (ITS1-ITS2) region. The 18S training set was built with the Silva eukaryote release 128 database. The RDP classifier gives a score (0 to 1) to each taxonomic depth of each OTUs. Each taxonomic depth having a score >= 0.5 is kept to reconstruct the final lineage. OTU/ASV are also blasted against the most recent NCBI nt database for complementary information.

9. Using taxonomic lineages obtained from step 8 combined with cluster abundance from step 7, a raw OTU/ASV table is generated. From that raw OTU/ASV table, an OTU/ASV table containing both bacterial and archeal organisms is generated. From this latter OTU/ASV table, a normalized OTU/ASV table (edgeR -[48,49]) is

generated]. If data consists of ITS amplicons, the same procedures are applied, but the raw OTU/ASV table is filtered to keep fungal organisms only. If the data is derived from 18S amplicons, the OTU/ASV table is filtered to keep eukaryotic organisms only.

10. A summary of read counts throughout the different steps of the pipeline is generated. This is useful to get a global outlook on the sequencing run: how many reads were sequenced, how many reads were filtered out after QC, how many OTUs/ASVs were generated, etc.

11. From these classified OTUs/ASVs, diversity metrics are obtained by aligning OTU/ASV sequences on a Greengenes core reference alignment[50] using the PyNAST aligner[17]. If data type is ITS or 18S, OTU/ASV sequences are aligned against a unite or silva eukaryote core alignment, respectively. For short amplicon data, Alignments are filtered to keep only the hypervariable region of the alignment. For long PacBio reads, the whole alignment is being kept.

12. A phylogenetic tree is then built from that alignment (from step 11) with FastTree[51]. Alpha (observed species) and beta (-weighted, unweighted UniFrac and Bray Curtis distances) diversity metrics and taxonomic summaries are then computed using the QIIME 1 software suite[17,52]. Along with the OTU/ASV tables, these last tables represent end results from the pipeline and can then be used to generate various types of plots and statistics computation.

Reads clustering and ASV methodology.

Our OTU generation procedure was implemented based on a procedure previously described (Lundberg et al., 2012) and uses either dnaclust of vsearch for the reads clustering step. Briefly, quality controlled reads/sequences are de-replicated at 100% identity. The dereplication step is necessary to lower data load for the clustering software as only one representative of many thousands of identical sequences are kept for clustering. Counts of each unique sequence representative are kept in sequence headers after the dereplication process. For instance for the AAD study, the fastq file holding quality controlled paired-end assembled reads holds 9,605,732 sequences. Once de-replicated, these sequences are actually regrouped into 822,807 sequences which represent a 11.7-fold data reduction. These dereplicated sequences are then clustered at 99% identity (dnaclust of vsearch). Clusters having an abundance of less than 25 reads are then discarded and the remaining clusters are then scanned for chimeras with UCHIME denovo and UCHIME reference[45] and clustered again at 97% identity (dnaclust or vsearch) to form the final clusters.

RDP classifier training sets.

The RDP classifier is a bayesian classifier whose purpose is to classify sequences against a training set. Existing training sets are based on 99% identity clustered versions of either Greengenes or Silva databases. The RDP database (not to be confused with the RDP classifier software) was also built in a similar manner. In order to improve resolution of classification, we built our own custom training sets using the whole Silva SSU (release 128) database. We had to semi-automatically and manually alter the classification of certain taxa in order to make the lineages unique and non-

conflicting. At the time of writing, we are using a training set based on the Silva 128 release. We also built our own training sets for 18S and ITS sequences. The taxonomic classification system for eukaryotic organisms is far more complex that what it is for the simpler bacterial kingdom. As such, additionally to the common kingdom, phylum, class, order, family and genus fields found in prokaryotic taxonomy, eukaryotic taxonomy includes ranks such as subphylum, subdivision, subclass, superorder, suborder and subfamily, which makes the task of generating consistent values for each rank challenging. Importantly, in order to obtain more resolution from taxonomic classifications, our training sets were generated using the whole databases of the Silva and Unite databases and not the clustered or "OTU" versions (i.e. clustered at various identity thresholds ranging from 95 to 99%) of these databases. Perl code used to generate training sets and training sets themselves are available in the following repository: https://github.com/jtremblay/RDP-training-sets.

Normalizing OTU/ASV tables with a multi-rarefaction procedure.

Normalization of ASVs or OTUs is a controversial topic[48,53]. Until a durable solution gets accepted by the microbial ecology research community, we favor a multi-rarefaction approach as a mean to generate a normalized OTU/ASV table. Briefly, the raw OTU table is first filtered for targeted microorganisms - if 16S primers were used, only OTUs/ASVs matching to Bacteria at the kingdom level will be kept for downstream steps. This filtered OTU/ASV table is then rarefied 500 times and the mean of each OTU/ASV of each sample is then computed so that a consensus rarefied table is obtained. Proceeding this way avoids the bias introduced by performing a single

random rarefaction, which inevitably leaves out low abundance microorganisms. This consensus rarefied table is then used for downstream analyses (alpha-, beta- diversity, taxonomic summaries, etc.).

Smart restart mechanism using a workflow management system.

Bioinformatics pipelines are intrinsically complex with many steps that need to be executed in a specific order. In order to improve productivity, pipelines should be executed on a compute cluster using a compute job scheduler (e.g. Torque, SLURM) supporting job dependencies. This way, the jobs of a complex pipeline can be submitted all at once to the job scheduler so that each job can be available for execution only when their depending job has successfully completed. For example, in a typical rRNA gene amplicon pipeline, the OTU/ASV generation job(s) can enter the waiting queue only when the quality control job it depends on have all been successfully completed. Only then, OTU/ASV generation job will enter the queue for execution. Many pipeline modules (software that generates scripts of job submissions) have been written and published[42,54]. A good pipeline framework should generate jobs, manage their dependencies and have a smart restart mechanism in case of job failure. In the context of a complex pipeline with hundreds to thousands of jobs, a smart restart mechanism is indispensable to gain productivity and save time determining which job failed. For instance if the execution of AmpliconTagger gets interrupted because of a job failure, it should be straightforward to identify exactly which job failed to properly execute. With a smart restart mechanism implementation, the pipeline framework should find, upon re-execution, which job actually failed to successfully complete and effectively rewrite them

for re-submission. Bioinformatics pipeline frameworks are also critical in that they allow sequencing data to be systematically analyzed in reproducible ways and that each step or job that it generates is parameterizable. For instance when analyzing quality controlled read data results, one can realize that the quality filtering parameters were too stringent given the quality score profiles of the input sequencing data. By slightly decreasing the quality filtering parameters and re-running the pipeline framework, all the downstream jobs affected by this modified parameter will be re-generated and re-submitted to the job scheduler. Proceeding with a pipeline framework also leaves traces of parameters used in all jobs should the data and analyses be revisited in the future.

Sequencing library preparation for mock community DNA.

Mock communities purified DNA was purchased form BEI resources (Manassas, VA) as HM-782D (even spike-in of total mock community). 16S rRNA gene amplicon libraries were prepared as described[55].

Availability of source code and requirements

Project Name: AmpliconTagger

Project Home Page: http://jtremblay.github.io/amplicontagger.html

Operating System: CentOS 7

Programming Languages: Python, Perl, R

Other requirements: pynast/1.2.2; perl/5.26.0; rdp_classifier/2.5; fasttree/2.1.10; FLASH/1.2.11; qiime/1.9.1; duk/1.051; dnaclust/3; fastx/0.0.13.2; python/2.7.5;

python/3.6.5; R/3.4.0; java/jdk1.8.0_144; blast/2.6.0+; deblur/1.0.4; vsearch/2.7.1; R/3.6.0

License: GNU GPL

Restriction: No licence required.

Code availability.

The AmpliconTagger pipeline wrapper code and Python, Perl and R scripts that are being called by AmpliconTagger are available here:

https://bitbucket.org/jtremblay514/nrc_pipeline_public/src/1.1/

https://bitbucket.org/jtremblay514/nrc_tools_public/src/1.1/

External software packages module install scripts are available here:

https://bitbucket.org/jtremblay514/nrc_resources_public/src/1.1/

A Docker image built on the CentOS 7 operational system which contains all necessary modules for full pipeline functionality is available for testing/evaluation purposes and running small datasets (https://cloud.docker.com/u/julio514/repository/docker/julio514/centos). Scripts used to generate RDP training sets are available here: and the training sets files are available on the docker image. The PipelineViewer web page is located here: http://jtremblay.github.io/PipelineViewer/amplicontagger.html and its source code is available here: https://github.com/jtremblay/PipelineViewer.

Availability of supporting data and materials

Sequencing for the indoor microbiome project is available through the ENA portal under accession number ERP005806. 16S rRNA amplicon sequence data for the AAD study is available in the NCBI's SRA portal under accession number SRP120170. PacBio full length 16S rRNA amplicons for the oral microbiome project is available under SRR56217[29-69]. ITS amplicons from the plant root microbiome transplant study are available under PRJNA301462. 18S rRNA gene amplicon data from the Chicago Michigan lake study is available under PRJNA294919/SRP063479. The even mock community reads are available under PRJNA510326. The staggered mock community is available under SRR2082918-20 and the PacBio mock libraries under SRR559331[4]-[7], SRR55933[19]-[20] and SRR559333[2]-[3]. All raw data and intermediate files used and generated for this study are available in GigaDB at accession number #xyz. All commands used to process all of the six datasets are also available in GigaDB.

Declarations

List of abbreviations

ASV = Amplicon Sequence Variant; OTU = Operational Taxonomic Unit; Gb = Giga-base; GB = Gigabyte; rRNA = ribosomal RNA; CCS = Circular Consensus Sequence; HPC = High Performance Computing; bp = base-pairs; GB = Gigabyte; MB = Megabyte.

Competing Interest

The authors declare that they have no competing interests.

Author contributions

JT planned the experimental design, wrote the software, analyzed the data and wrote the manuscript. EY edited the manuscript.

Acknowledgments

We wish to acknowledge Compute Canada for access to both the Waterloo University (Graham system) and McGill University (Guillimin system) High Performance Computing (HPC) infrastructures. We thank Charles W Greer for editing the manuscript and Jessica Wasserscheid for editing the user guide.

Figure legends

Figure 1. Comparison between deblur, dnaclust and vsearch as implemented in AmpliconTagger and Qiime2-vsearch, Qiime2-dada2 and Qiime2-deblur for a) taxonomic profiles, b) beta diversity and c) alpha diversity of mock community samples (16S V4 region; 2x250 bp).

Figure 2. Resources consumption for investigated datasets and each OTUs/ASVs generation method. There are no common core steps for Qiime2-dada2 workflow as raw reads were submitted to dada2 directly.

References

1. Human Microbiome Project Consortium. A framework for human microbiome research. Nature. 2012;486:215–21.

2. Human Microbiome Project Consortium. Structure, function and diversity of the healthy human microbiome. Nature. 2012;486:207–14.

3. Integrative HMP (iHMP) Research Network Consortium. The Integrative Human Microbiome Project: dynamic analysis of microbiome-host omics profiles during periods of human health and disease. Cell Host Microbe. 2014;16:276–89.

4. Thompson LR, Sanders JG, McDonald D, Amir A, Ladau J, Locey KJ, et al. A communal catalogue reveals Earth's multiscale microbial diversity. Nature. 2017;551:457–63.

5. Watson-Haigh NS, Shang CA, Haimel M, Kostadima M, Loos R, Deshpande N, et al. Next-generation sequencing: a challenge to meet the increasing demand for training workshops in Australia. Brief Bioinform. 2013;14:563–74.

6. Daber R, Sukhadia S, Morrissette JJD. Understanding the limitations of next generation sequencing informatics, an approach to clinical pipeline validation using artificial data sets. Cancer Genet. 2013;206:441–8.

7. Li P-E, Lo C-C, Anderson JJ, Davenport KW, Bishop-Lilly KA, Xu Y, et al. Enabling the democratization of the genomics revolution with a fully integrated web-based bioinformatics platform. Nucleic Acids Res. 2017;45:67–80.

8. Shringarpure SS, Carroll A, De La Vega FM, Bustamante CD. Inexpensive and Highly Reproducible Cloud-Based Variant Calling of 2,535 Human Genomes. PLoS One. 2015;10:e0129277.

9. Afgan E, Baker D, van den Beek M, Blankenberg D, Bouvier D, Čech M, et al. The Galaxy platform for accessible, reproducible and collaborative biomedical analyses: 2016 update. Nucleic Acids Res. 2016;44:W3–10.

10. Alkema W, Boekhorst J, Wels M, van Hijum SAFT. Microbial bioinformatics for food safety and production. Brief Bioinform. 2016;17:283–92.

11. Hess M, Sczyrba A, Egan R, Kim T-W, Chokhawala H, Schroth G, et al. Metagenomic discovery of biomass-degrading genes and genomes from cow rumen. Science. 2011;331:463–7.

12. Manaka A, Tokue Y, Murakami M. Comparison of 16S ribosomal RNA gene sequence analysis and conventional culture in the environmental survey of a hospital. J Pharm Health Care Sci. 2017;3:8.

13. Martineau C, Li X, Lalancette C, Perreault T, Fournier E, Tremblay J, et al. Serratia marcescens Outbreak in a Neonatal Intensive Care Unit: New Insights from Next-Generation Sequencing Applications. J Clin Microbiol [Internet]. 2018;56. Available from: http://dx.doi.org/10.1128/JCM.00235-18

14. Hewitt KM, Mannino FL, Gonzalez A, Chase JH, Caporaso JG, Knight R, et al. Bacterial diversity in two Neonatal Intensive Care Units (NICUs). PLoS One. 2013;8:e54703.

15. Bokulich NA, Mills DA, Underwood MA. Surface microbes in the neonatal intensive care unit: changes with routine cleaning and over time. J Clin Microbiol. 2013;51:2617–24.

16. Deurenberg RH, Bathoorn E, Chlebowicz MA, Couto N, Ferdous M, García-Cobos S, et al. Application of next generation sequencing in clinical microbiology and infection prevention. J Biotechnol. 2017;243:16–24.

17. Caporaso JG, Kuczynski J, Stombaugh J, Bittinger K, Bushman FD, Costello EK, et al. QIIME allows analysis of high-throughput community sequencing data. Nat Methods. 2010;7:335–6.

18. Schloss PD, Westcott SL, Ryabin T, Hall JR, Hartmann M, Hollister EB, et al. Introducing mothur: open-source, platform-independent, community-supported software for describing and comparing microbial communities. Appl Environ Microbiol. 2009;75:7537–41.

19. Bolyen E, Rideout JR, Dillon MR, Bokulich NA, Abnet C, Al-Ghalith GA, et al. QIIME 2: Reproducible, interactive, scalable, and extensible microbiome data science. PeerJ Preprints. 2018.

20. Callahan BJ, McMurdie PJ, Rosen MJ, Han AW, Johnson AJ, Holmes SP. DADA2: High resolution sample inference from amplicon data [Internet]. Available from: http://dx.doi.org/10.1101/024034

21. Bourgey M, Dali R, Eveleigh R, Chen KC, Letourneau L, Fillon J, et al. GenPipes: an open-source framework for distributed and scalable genomic analyses. GigaScience [Internet]. 2019;8. Available from: https://doi.org/10.1093/gigascience/giz037

22. Tremblay J, Singh K, Fern A, Kirton ES, He S, Woyke T, et al. Primer and platform effects on 16S rRNA tag sequencing. Front Microbiol. 2015;6:771.

23. Lax S, Smith DP, Hampton-Marcell J, Owens SM, Handley KM, Scott NM, et al. Longitudinal analysis of microbial interaction between humans and the indoor environment. Science. 2014;345:1048–52.

24. Searle D, Sible E, Cooper A, Putonti C. 18S rDNA dataset profiling microeukaryotic populations within Chicago area nearshore waters. Data Brief. 2016;6:526–9.

25. MacPherson CW, Mathieu O, Tremblay J, Champagne J, Nantel A, Girard S-A, et al. Gut Bacterial Microbiota and its Resistome Rapidly Recover to Basal State Levels after Short-term Amoxicillin-Clavulanic Acid Treatment in Healthy Adults. Sci Rep. 2018;8:11192.

26. Yergeau E, Bell TH, Champagne J, Maynard C, Tardif S, Tremblay J, et al. Transplanting Soil Microbiomes Leads to Lasting Effects on Willow Growth, but not on the Rhizosphere Microbiome. Front Microbiol. 2015;6:1436.

27. Earl JP, Adappa ND, Krol J, Bhat AS, Balashov S, Ehrlich RL, et al. Species-level bacterial community profiling of the healthy sinonasal microbiome using Pacific Biosciences sequencing of full-length 16S rRNA genes. Microbiome. 2018;6:190.

28. Wang Y, Zhang J, Chen X, Jiang W, Wang S, Xu L, et al. Profiling of Oral Microbiota in Early Childhood Caries Using Single-Molecule Real-Time Sequencing. Front Microbiol. 2017;8:2244.

29. Rognes T, Flouri T, Nichols B, Quince C, Mahé F. VSEARCH: a versatile open source tool for metagenomics. PeerJ. 2016;4:e2584.

30. Ghodsi M, Liu B, Pop M. DNACLUST: accurate and efficient clustering of phylogenetic marker genes. BMC Bioinformatics. 2011;12:271.

31. Amir A, McDonald D, Navas-Molina JA, Kopylova E, Morton JT, Zech Xu Z, et al. Deblur Rapidly Resolves Single-Nucleotide Community Sequence Patterns. mSystems [Internet]. 2017;2. Available from: http://dx.doi.org/10.1128/mSystems.00191-16

32. Callahan BJ, McMurdie PJ, Holmes SP. Exact sequence variants should replace operational taxonomic units in marker-gene data analysis. ISME J. 2017;11:2639–43.

33. Lindahl BD, Nilsson RH, Tedersoo L, Abarenkov K, Carlsen T, Kjøller R, et al. Fungal community analysis by high-throughput sequencing of amplified markers--a user's guide. New Phytol. 2013;199:288–99.

34. Westcott SL, Schloss PD. De novo clustering methods outperform reference-based methods for assigning 16S rRNA gene sequences to operational taxonomic units. PeerJ. 2015;3:e1487.

35. He Y, Caporaso JG, Jiang X-T, Sheng H-F, Huse SM, Rideout JR, et al. Stability of operational taxonomic units: an important but neglected property for analyzing microbial diversity. Microbiome. 2015;3:20.

36. Segata N. On the Road to Strain-Resolved Comparative Metagenomics. mSystems [Internet]. 2018;3. Available from: http://dx.doi.org/10.1128/mSystems.00190-17

37. Riley MA, Lizotte-Waniewski M. Population Genomics and the Bacterial Species Concept. Methods in Molecular Biology. 2009. p. 367–77.

38. Glassman SI, Martiny JBH. Broadscale Ecological Patterns Are Robust to Use of Exact Sequence Variants versus Operational Taxonomic Units. mSphere [Internet]. 2018;3. Available from: http://dx.doi.org/10.1128/mSphere.00148-18

39. Nearing JT, Douglas GM, Comeau AM, Langille MGI. Denoising the Denoisers: an independent evaluation of microbiome sequence error-correction approaches. PeerJ. 2018;6:e5364.

40. Filippidou S, Junier T, Wunderlin T, Lo C-C, Li P-E, Chain PS, et al. Under-detection of endospore-forming Firmicutes in metagenomic data. Comput Struct Biotechnol J. 2015;13:299–306.

41. Wesolowska-Andersen A, Bahl MI, Carvalho V, Kristiansen K, Sicheritz-Pontén T, Gupta R, et al. Choice of bacterial DNA extraction method from fecal material influences community structure as evaluated by metagenomic analysis. Microbiome. 2014;2:19.

42. Bourgey M, Dali R, Eveleigh R, Chen KC, Letourneau L, Fillon J, et al. GenPipes: an open-source framework for distributed and scalable genomic analyses [Internet]. 2018. Available from: http://dx.doi.org/10.1101/459552

43. Knights D, Kuczynski J, Charlson ES, Zaneveld J, Mozer MC, Collman RG, et al. Bayesian community-wide culture-independent microbial source tracking. Nat Methods. 2011;8:761–3.

44. Magoč T, Salzberg SL. FLASH: fast length adjustment of short reads to improve genome assemblies. Bioinformatics. 2011;27:2957–63.

45. Edgar RC, Haas BJ, Clemente JC, Quince C, Knight R. UCHIME improves sensitivity and speed of chimera detection. Bioinformatics. 2011;27:2194–200.

46. Wang Q, Garrity GM, Tiedje JM, Cole JR. Naive Bayesian classifier for rapid assignment of rRNA sequences into the new bacterial taxonomy. Appl Environ Microbiol. 2007;73:5261–7.

47. Quast C, Pruesse E, Yilmaz P, Gerken J, Schweer T, Yarza P, et al. The SILVA ribosomal RNA gene database project: improved data processing and web-based tools. Nucleic Acids Res. 2013;41:D590–6.

48. McMurdie PJ, Holmes S. Waste Not, Want Not: Why Rarefying Microbiome Data Is Inadmissible. PLoS Comput Biol. 2014;10:e1003531.

49. Robinson MD, McCarthy DJ, Smyth GK. edgeR: a Bioconductor package for differential expression analysis of digital gene expression data. Bioinformatics. 2010;26:139–40.

50. DeSantis TZ, Hugenholtz P, Larsen N, Rojas M, Brodie EL, Keller K, et al. Greengenes, a chimera-checked 16S rRNA gene database and workbench compatible with ARB. Appl Environ Microbiol. 2006;72:5069–72.

51. Price MN, Dehal PS, Arkin AP. FastTree 2--approximately maximum-

likelihood trees for large alignments. PLoS One. 2010;5:e9490.

52. Kuczynski J, Stombaugh J, Walters WA, González A, Caporaso JG, Knight R. Using QIIME to analyze 16S rRNA gene sequences from microbial communities. Curr Protoc Bioinformatics. 2011;Chapter 10:Unit 10.7.

53. Weiss S, Xu ZZ, Peddada S, Amir A, Bittinger K, Gonzalez A, et al. Normalization and microbial differential abundance strategies depend upon data characteristics. Microbiome. 2017;5:27.

54. Leipzig J. A review of bioinformatic pipeline frameworks. Brief Bioinform. 2017;18:530–6.

55. Yergeau E, Michel C, Tremblay J, Niemi A, King TL, Wyglinski J, et al. Metagenomic survey of the taxonomic and functional microbial communities of seawater and sea ice from the Canadian Arctic. Sci Rep. 2017;7:42242.

 Click here to download Table tables.docx

Table1. Details of investigated datasets.

Study	Targeted gene and region	Average reads length of paired assembled fragments[1] (mean +/- standard deviation)	Sequencing configuration	Number of reads	Number of basepairs	Number of samples	File size of sequencing data (gzip compressed)
Even mock community (this study)	16S bacteria/archaea; V4	250.3 +/- 0.7 bp	Illumina 2x250 bp	1,987,408	0.50 Gb	4	375 MB
Staggered mock community[22]	16S bacteria/archaea; V4	250.2 +/- 0.7 bp	Illumina 2x250 bp	289,434	0.072 Gb	3	30 MB
Indoor microbiome[23]	16S bacteria; V3-V4 region	No assembled fragments, single end reads of 151 bp	Illumina 1x150 bp	111,093,697	16.8 Gb	1625	6.9 GB
Chicago nearshore water profiling[24]	18S eukaryotes; 1181F-1624R	250.5 +/- 3.1 bp	Illumina 2x150 bp	19,359,618	4.86 Gb	89	2.3 GB
Antibiotic-Associated Diarrhea[25]	16S bacteria/archaea; V4	250.4 +/- 0.8 bp	Illumina 2x250 bp	22,003,478	3.3 Gb	276	2.9 GB
Soil microbiome transplant[26]	Fungi ITS; ITS1	249.0 +/- 7.9 bp	Illumina 2x250 bp	30,775,636	7.72 Gb	94	4.1 GB
PacBio mock community[27]	16S bacterial; Full length	No assembled fragments, single end reads of 1400 bp[2]	PacBio Single end sequencing	93,905	0.14 Gb	8	35 MB
Oral Microbiota[28]	16S bacterial; Full length	No assembled fragments, single end reads of 1400 bp[2]	PacBio Single end sequencing	627,138	0.88 Gb	40	117 MB

[1]:These are the reads that are sent for OTUs/ASVs generation after having been paired-end assembled (for paired-end sequencing) and controlled for quality as described in methods.

[2]: PacBio long reads were trimmed at 1,400 bp before processing them in AmpliconTagger.
bp = base-pairs, GB = Gigabyte, MB = Megabyte.

Table 2. Number of reads and OTUs/ASVs throughout AmpliconTagger's execution.

Project	OUT/ASV generation method	Total reads	Contaminants reads	Phix reads	Non contaminant and non phix reads	Non contaminant and non phix reads 1	Non contaminant and non phix reads 2	Reads 1 QC passed	Assembled reads	Assembled reads QC passed	Clustered or dereplicated sequences	Number of clusters or dereplicated sequences
Mock community (V4 16S; paired-end)	deblur	2,602,808	8	27	2,276,776	1,138,388	1,138,388	-	1,112,137	1,055,828	639,767	67
	dnaclust										801,550	66
	vsearch										1,001,083	34
	Qiime2-deblur		-	-	-	-	-	-	966,899	966,829	620,421	46
	Qiime2-vsearch		-	-	-	-	-	-	966,899	966,829	966,829	1,074
	Qiime2-dada2		-	-	-	-	-	-	-	-	282,708	151
Indoor Microbiome (V4 16S; single end)	deblur	111,093	48,996	-	111,044,701	-	-	107,944,223			73,493,917	27,833
	dnaclust										95,113,536	18,338
	vsearch										100,277,958	14,334
Lake Michigan (1181F-1624R 18S; paired-end)	deblur	19,359,618	275,694	4,521	18,803,930	9,401,965	9,401,965	-	7,982,424	3,156,643	1,626,113	580
	dnaclust										2,397,521	504
	vsearch										2,538,513	468
	deblur	22,003,478	151	809	22,001,808	11,000,904	11,000,904	-	10,847,216	9,582,952	5,793,587	1,674
	dnaclust										7,780,216	1,021

**AAD (V4 16S; paired-end)**	**vsearch**										8,453,098	838
**Plant microbiome transplant (ITS1 ITS; paired-end)**	**deblur**	30,775,636	174,471	5,770,656	24,816,770	12,408,385	12,408,385		9,803,309	7,469,192	3,483,561	1,093
	**dnaclust**										6,308,659	1,225
	**vsearch**										7,273,795	1,091
**Mock community (full length 16S; single end)**	**deblur (`--min-reads 1`*)**	93,905	-	-	93,905	93,905	-	61,058	-	-	42,151	42,135
	**dnaclust**										55,249	751
	**vsearch**										47,182	246
**Oral microbiome (full length 16S; single end)**	**deblur-mr1 (`--min-reads 1`*)**	627,138	-	-	627,138	627,138	-	562,896	-	-	242,071	114,783
	**deblur-mr3 (`--min-reads 3`*)**										18,083	757
	**dnaclust**										353,676	10,022
	**vsearch**										385,900	5,180

* Setting Deblur`--min-reads` to 2 yielded 12 ASVs only and was therefore not investigated further. `--min-reads` was set to 25 for short amplicon data.

** Only some of the read counts statistics were available for deblur and non for dada2.

Table 3. Mantel *r* statistics comparing distance matrices of each ASVs/OTUs generation method for each project. Each *r* statistic had a *p* value < 0.001.

	Weighted UniFrac			Bray-Curtis		
	**deblur vs dnaclust**	**deblur vs vsearch**	**dnaclust vs vsearch**	**deblur vs dnaclust**	**deblur vs vsearch**	**dnaclust vs vsearch**
**Mock community (V4 16S; paired-end)**	0.971	0.997	0.973	0.79	0.221	0.4
**Indoor Microbiome (V4 16S; single end)**	0.941	0.936	0.968	0.991	0.967	0.98
**Lake Michigan (1181F-1624R 18S; paired-end)**	0.971	0.980	0.984	0.996	0.995	0.998
**AAD (V4 16S; paired-end)**	0.95	0.987	0.963	0.967	0.941	0.97
**Plant microbiome transplant (ITS1 ITS; paired-end)**	0.258	0.570	0.414	0.903	0.893	0.991
**Mock community (full length 16S; single end)***	-	-	0.851	-	-	0.844

**Oral microbiome (full length 16S; single end)***	0.721	0.744	0.777	0.268	0.213	0.928

*: deblur run with parameter `–min-reads 1`

-: Mantel *r* statistics not computable

Figure 1

Click here to download Figure Figure_1.pdf

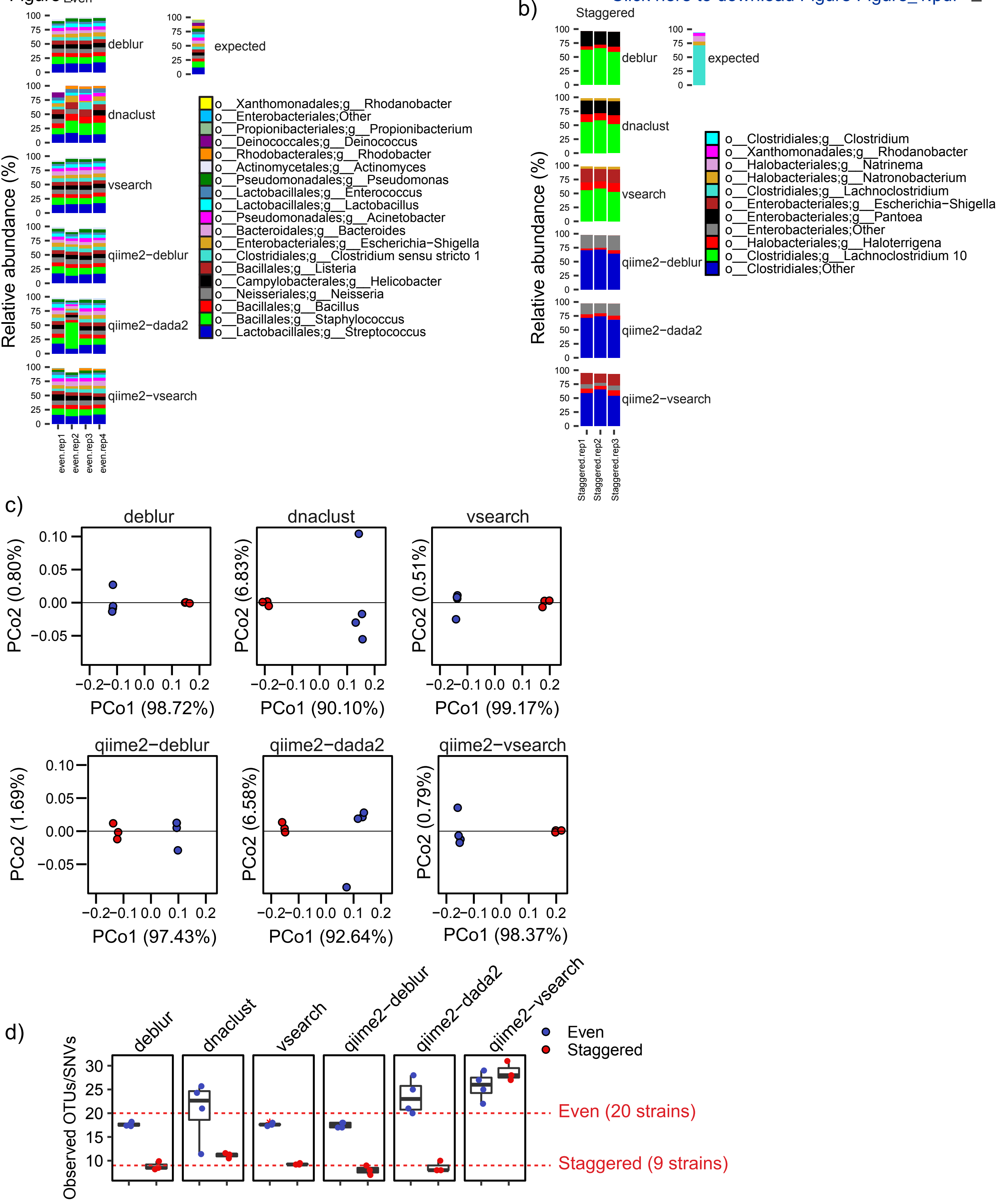

Figure 2 Click here to download Figure Figure_2.pdf

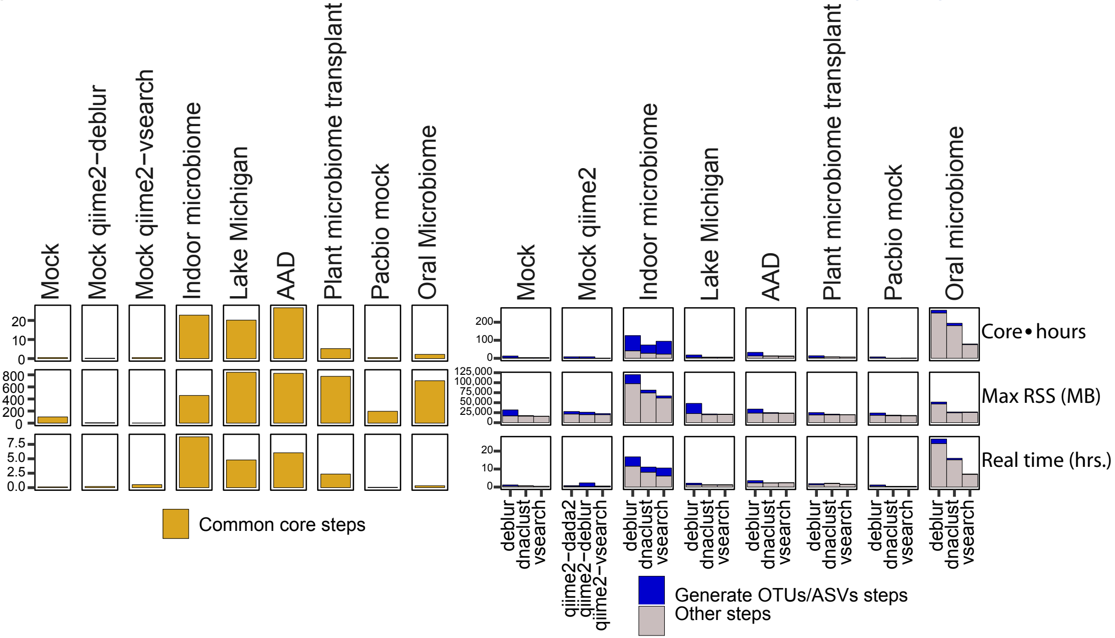

Additional file 1

Click here to access/download
Supplementary Material
Additional_file_1.pdf

Additional file 1 figure legends

Click here to access/download
Supplementary Material
Additional_file_1_figure_legends.docx

Additional file 2

Click here to access/download
Supplementary Material
additional_file_2.tar.gz.7z

Additional file 3

Click here to access/download
Supplementary Material
additional_file_3.docx

Additional file 4

Click here to access/download
Supplementary Material
Additional_file_4.html

Supplementary tables

Click here to access/download
Supplementary Material
supplementary_tables.xlsx

GIGA-D-19-00051
Systematic processing of rRNA gene amplicon sequencing data Julien Tremblay; Etienne Yergeau
GigaScience

Dear Dr Tremblay,

Thank you for considering GigaScience. Peer review of your manuscript is now complete and, in the light of the reports, and my own assessment as Editor, I regret to inform you that your manuscript cannot be accepted for publication in GigaScience. Unfortunately significant additional work is required to address the issues of usability and documentation, as well as convincing comparisons and testing against the state of the art.

Please find the reviewers' reports at the end of this email. Please also take a moment to check our website at https://www.editorialmanager.com/giga/ for any additional comments that were saved as attachments.

If at some stage you are able to fully address these concerns, you may wish to submit to GigaScience with the revised manuscript. If you are able to do this a full cover letter, explaining the revisions made, should accompany the submission.

I wish you every success with your research and hope that you will consider us again in the future.

Best wishes,

Scott Edmunds, Ph.D.
GigaScience
www.gigasciencejournal.com

Reviewer reports:

Reviewer #1: In this manuscript, they author have developed a new analytic pipeline, AmpliconTagger, 16S/18S/ITS rRNA gene amplicons sequencing. The pipeline integrated multiple analytic algorithms and support multiple different input types. The article is well-written, and the analysis is well designed. However, I have following concerns from the overall design and detailed analysis of this project:

1. The pipeline is designed for experienced bioinformaticians who actually does not really depend on established pipeline to perform data analysis. In most cases, pipeline is designed from unexperienced computational biologist or wet bench scientist for them to perform bioinformatic analysis easily. As mentioned by the author, since each analytic step requires multiple parameters to be optimized, the tools which could optimize combinations of parameters and increase the biological significance of output automatically would be more useful than a pipeline just including more options and parameters.

We understand the reviewer’s concern and we are at the moment unfortunately unable to generate a method that would automatically generate all optimal parameters in a satisfactory way as there are much factors to consider in this (reads quality, sequencing configuration, community complexity, etc.), but would definitely be something worthwhile exploring in the future. We also favor a paradigm in which the pipeline operator avoids relying on automatic parameters, which “forces” the operator to

know exactly what data types has to be processed by the pipeline (reads configuration, what primer pair and consequently what microorganisms are being targeted for amplification, what database to use for taxonomic assignment, etc.), which in the end motivates the operator to gain a deep enough understanding of all the steps required to analyze data. In that regard, we wrote, in the manuscript's revised version, an extensive user guide that among others, discusses the key parameters important for the execution of our pipeline. On the GigaDB resource, should our manuscript is accepted, we hope to include all the .ini files of all projects we report in the manuscript, which should cover the vast majority of parameters definitions for most used amplicon sequencing data types. In addition, the majority of amplicon data types in the field of microbial ecology is currently generated as MiSeq paired-end reads which usually overlaps enough to generate merged amplicon sequences (primer pairs V4, V3-V4, V4-V5, etc) and the example datasets (AAD_demo) we provide and discuss in the Docker image and user guide (additional file 4 in the revised manuscripts) follows these specifications: V4 primer pair with overlapping forward and reverse reads.

2. Long PacBio amplicons seems have high dissimilarity when using different distance metric. And since the results are very different, when which analytic methods give a more "correct" result for long PacBio reads?

We thank the reviewer for expressing this important concern. In consequence, we improved the manuscript on two elements regarding the analysis of PacBio data:

1) We added a PacBio mock community dataset to compare our pipeline's outputs against what is expected. The analysis of the mock community made us realize that the vsearch and dnaclust parameters seemed to be adequate, but that the deblur `–min-reads 2` or 3 (which rejects reads having an abundance less than 2 and 3 respectively) ended up in the rejection of the majority of the reads in the dataset, inevitably affecting downstream diversity metrics. We did not report ASV results for `–min-reads` 3 as we ended up with 4 ASVs only, which didn't make sense to pursue. However, setting the `–min-reads` parameter to 1, ended up in keeping the majority of reads for ASV generation. However, this resulted in a situation where all ASVs had an abundance of 1 (Table 2) with almost no ASVs overlapping across samples. In consequence, the downstream alpha and beta diversity metrics does not really have any useful meaning as shown in figure S6b). Taxonomic summary obtain with deblur was highly similar to the ones obtained with dnaclust and vsearch.

2) We integrated an additional deblur analysis with the `–min-reads 1` parameter (in addition to the original `–min-reads 3` parameter) for the PacBio oral microbiome dataset (Table 2 and figure S7). The results obtained with –min-reads 1, but not –min-reads 3 (mr1 in the figure) shows similar taxonomic profiles as the ones obtained with dnaclust and vsearch. However, because both parameters distorts the resulting ASV abundance profiles so much - very few ASVs with `–min-reads 3` and really high abundance of singlets with `–min-reads 1` – resulting alpha and beta diversity values are in the end meaningless as shown in figure S7b) and c) (and also figure 6b). We also investigated the use of more lenient parameters `--mean-error <float> --indel-prob <float>` with very little improvement on the number of singlets.

Consequently, we believe that ASVs may not the be the best fit to appropriately to analyze long PacBio amplicon reads and we lean toward using a clustering workflow to analyse long PacBio amplicons. That said, performing an ASV analysis were all ASVs are kept (including the singlets) might be appropriate for taxonomic profiles (but not alpha and beta diversity) assessment.

Lines 215-228, 354-358, and 267-375 in the revised manuscript discuss these matters.

3. Looks like the author use comparison among different methods to self-validate the accuracy of this pipeline, but is there a test case measured with biological significance? A case with known taxonomic summaries and diversity metrics.

We thank the reviewer for this remark. We therefore added an additional set of mock community data in the revised manuscript. We now report and discuss two mock community short amplicon data set (now figure 2) analyzed with AmpliconTagger (i.e. deblur, dnaclust and vsearch) and also with one qiime2-deblur based and one qiime2-dada2 workflows. This new data is reported and discussed in the revised version of the manuscript at lines 145-160. As an additional note, we replaced the original mock community dataset with another one (table 1) as the original one showed high variation in one of the replicate, which we feared could introduce confusion in readers.

Overall, I think this article is well-written and can be accepted after revision.

Reviewer #2: Tremblay and Yergeau present a potentially more flexible and alternative software package to be used to analyze amplicon sequencing data. I have many concerns with the currently presented report. I have tried to limit my comments to what I think are the most pressing concerns and have separated them into major and minor comments. I hope the authors find these comments helpful and constructive.

Major Comments:

Line 39-41: mothur and qiime have command line functionality that allow what the authors state in the abstract, "...leverage modern HPC capabilities and perform tweaking and optimization operations."
We do not put into question the fact that Qiime and Mothur can be executed from the command line. However, they do not have automation of systematic job generation, submission and smart restart mechanism which is the objective of the current manuscripts: present an amplicon pipeline integrated into a workflow management system that takes care of all the job generation, submission, dependency and smart restart in case of failure. We clarified this at line 101.

The authors need to define what they mean by "advanced customization". As an example, mothur allows for tweaking of hundreds of parameters on the command line while using a submission system. Surely this would fit under advanced customization.
We precised our thoughts on this matter at lines 80-82.

Line 96, the authors mention, "metagenomics." Do the authors mean 16S here or do they mean actual metagenomic sequencing? It is unclear, since most of the text before and after focus solely on 16S or amplicon approaches.
We have clarified our meaning at lines 96-97. In the context of this manuscript metagenomics include both shotgun metagenomics and amplicon sequencing. This sentence really meant to say that metagenomics in general (including 18S, 16S and ITS amplicon sequencing and shotgun metagenomics) would benefit from more robust and flexible ways of performing systematic analyses.

Both qiime and mothur have options to change the type of clustering completed. This pipeline that is reported by the authors does not seem to allow flexibility in this regard.
Our pipeline allows to customize a vast array of parameters for clustering or ASV generation. In the main methods text of the manuscript, we refer to default parameters that we used for the data analyzed for the manuscript, but lots of these parameters can be customized as described in the user-guide now available in the revised version as additional file 4 – mentioned in the text at line 143.

Additionally, it is not clear if changing the clustering algorithm is allowed at all within the current clustering framework.
As stated in the manuscript's introduction (lines 107-108), analyses (lines 127-128), figure S1 and elsewhere in the results and discussion sections, our pipeline support supports two clustering algorithms (dnaclust and vsearch). We further expand on this in the user guide (additional file 4) available in the revised manuscript supplementary material.

Even for advanced users it is unclear why three different languages are used to create the program. This makes it seem more like an aggregated workflow that is made transferable with tools like docker rather than a stand-alone program
Never have we pretended our pipeline to be a stand-alone program. On the contrary, we explicitly acknowledge in the introduction that the bioinformatics ecosystem contains an ever-increasing amount

of software and that our pipeline addresses this problematic by providing an efficient way of integrating these packages in a coherent whole. That is actually what distinguish AmpliconTagger from the other available solutions and in the end is the reason for its existence.

To add further clarity, the workflow management system is coded in Python. The software packages and scripts that are being implemented in the workflow management system can actually be written in any language. For instance the RDP classifier is written in Java, Deblur in Python and Vsearch in C. The installation instructions which contains all the URL or code repository addresses of these external packages are included in this code repository (nrc_resources_public). Our internal scripts were in majority written in Perl and R (repo nrc_tools_public). The reader is now referred to the user guide (additional file 4) at line 143 in the revised manuscript.

Not everyone has a SLURM scheduler and the code provided in the supplemental assumes that they do.
We used the SLURM scheduler, because it is the scheduler that is implemented in production in many modern high performing computing facilities and is the one available on the compute cluster that we use. The pipeline could be adapted to Torque, but is not officially supported. Our pipeline supports the generation of jobs for interactive execution, by specifying the `-j batch` argument instead of `-j slurm`. Doing so, instructs the pipeline to generate jobs free of all the SLURM syntax and can be executed directly from the command line, on an interactive node for instance. Again, the reader is now referred to the user guide (additional file 4) at line 143 in the revised manuscript.

A considerable amount of time is spent talking about the importance of using an HPC scheduling system when every single program that has been previously created and are used heavily by bioinformatic analysts already allows for this to be doable.
We certainly are aware that about any informatics program can be executed on a compute cluster. The main feature of our pipeline however is that it easily allows the submission of many jobs at once that will all be executed in the correct order following their job dependency network. This is now clarified in the user guide/additional file 4 in the revised manuscripts.

The true comparison is whether the standard approaches that are command line based provide the same functionality. Many of the described functions can be executed using other programs and require less dependencies. Additionally, the authors need to explain why using a program workflow with more dependencies and seemingly less flexibility is desirable.

We definitely agree with the reviewer that there many programs out there that can perform a given task or functionality. As stated in the introduction, this is one of the core motivation behind writing our pipeline: to be able to incorporate any of these methods with a minimum of coding, which will allow an experienced bioinformatics analyst to generate and submit jobs that will be executed in due time according to their dependency network.
We respectfully disagree with the reviewer that our pipeline is less flexible. On the contrary it so flexible, that it allows the integration of virtually any software/package. For instance, if say Vsearch3 was to be released tomorrow, we would only have to 1) install the software, 2) write a minimal amount of code in the `./pipelines/amplicon_tagger.py` and `./bio/rrna_amplicons.py` to provide instruction on the commands to be written upon the workflow management system's execution and 3) submit the generated jobs. That said, we acknowledge that these details were lacking in the original manuscript and we elaborated more on this in the user guide (additional file 4) now available in the manuscript's supplementary material.

Even with the Docker image many of the relied upon software will be updated (e.g. the R version is over a year old). Also, newer software versions could provide better speed or analysis but could break the pipeline that the authors are writing about. This seems to be a major limitation since much of the pipeline is borrowed from other programs.
The reviewer is correct, our pipeline, like every software or code in general will need to be periodically updated. In that regard, the code repository we provided will be regularly updated – which of course includes thorough testing before each release to make sure that new additions or modification (including new software versions) did not break the flow of the pipeline. In contrast to the reviewer, we don't see this as a limitation, but rather as a strength of our pipeline which can be easily updated to satisfy the requirements of an updated external software package. The reviewer states that "newer software versions could provide better speed or analysis" – This is precisely one of the strength of our pipeline: to allow efficient integration of new software, which we state at lines 249-250 and 45-46.

With regards to the, "Smart restart mechanism using a workflow management system" section: What the authors describe in this section could also be accomplished with a Makefile without needing to have the correct scheduler installed on the HPC.
Here we take the opportunity to state that our workflow management system (WMS) and the job scheduler are two distinct things. The WMS generates jobs that can either be written in the objective of being submitted to a job Scheduler (options `-j slurm`) or to be executed directly on the command line (option `–j bash`). We believe it would be impractical to write a similar WMS using Makefile as it does not, by its inherent nature, offer the same flexibility as Python to generate jobs (Job objects described in the user guide) and manage their dependencies which is certainly more complex than writing simple scripts. Again we acknowledge this was not sufficiently explained in the original manuscript and the user guide (additional file 4) now discusses these topics at length.

Alternatively, custom python or perl scripts could accomplish this and allow the pipeline to be more contained and independent.
We thank the reviewer for acknowledging that a workflow management system could be written in Python or Perl. This is exactly what we present in this manuscript: a fully functioning WMS written in Python.

Minor Comments:

"M" in mothur is not capitalized
We thank the reviewer for noting this typo. This has been corrected.

Table 2 is odd and should be reformatted to fit the theme of table 2
We thank the reviewer for this suggestion. We assumed the reviewer meant to reformat the theme of table 2 to the one of table 1? If that is the case this has been done in the new table 2.

Reviewer #3: The authors here have discussed a pipeline by implementing available OTU and ASV methods as a docker image to make use of these methods on a HPC simpler for marker gene analysis for high throughput sequencing and significant effort has gone towards the development of a pipeline for their purposes.
We thank the reviewer for appreciating our work.

While the authors have taken considerable efforts to develop a docker image and describe the available tools for managing the pipelines, stating the goal of the study was to show the modular nature of their pipeline and available microbiome profiling results, a reproducible example discussing the involved steps at every decision process of the pipeline of processing with results at these steps will encourage users to adopt the docker image in their own settings. The non-availability of these steps makes it difficult to understand the provided results, their pre-processing and post processing steps along with the understanding of microbiome profiling steps. A reproducible example will also provide support to their interactive or single batch job processing approach which will be useful for a production context.
We fully agree with the reviewer's suggestion to include a reproducible example discussing the pipeline's steps. In the revised manuscript, we added an exhaustive user guide (additional file 4), among others describing in detail each step of our pipeline for the AAD_demo dataset (a subset of the AAD study presented in table 1) which represents paired-end sequencing (2x250 bp) of the V4 region of the 16S rRNA gene.

The availability of OTU and ASV methods as an option for the user is one of the strongest points of this pipeline. However, while reviewing the methods it seems that the authors suggest to trim all the reads to a fixed length. While trimming to a fixed length is useful for 16S genes the same is not necessarily true for ITS datasets. Trimming ITS datasets to a fixed length results in loss of resolution leading to creation of false clusters/ASVs as also observed in the results. This is inherently a problem with dnaclust, vsearch and deblur which require the reads to be trimmed to a fixed length. If the authors can provide for retaining the length variability of ITS datasets, the results for ITS datasets will be beneficial.
We agree with the reviewer that ITS should indeed not be trimmed at the same length because of the high variability typically observed in their amplicon length which was discussed in the original manuscript (lines 327-328 and figure S8 in the revised manuscript). The point raised by the reviewer is due to lack of clarity from our part in the description of the method. To summarize, no reads trimming is performed for any of the paired-end amplicon datasets. As now explicitly discussed in the user guide (additional file 4). In the original manuscript, we really meant to say that reads should be trimmed for 454, IonTorrent and PacBio data types, because they can vary significantly in length. In practice, Illumina reads are of the same length and therefore do not need to be trimmed at the same length before entering clustering process. That said, for historical reasons, we do have a step that can optionally trim reads before reads merging: earlier versions of the FLASH software mishandled the reads merging process when paired-end reads were overlapping too much. In consequence, we implemented a step to trim both R1 and R2 before merging them with FLASH which substantially improved the number of merged read pairs. Newer versions of FLASH do not have this issue anymore, but we decided to leave that optional step of trimming reads as an additional layer of control for unforeseen scenarios. We re-wrote bullet point #3) in the methods section describing our workflow (lines 512-518 in the revised manuscripts).

The analysis of Pacbio datasets using dnaclust, vsearch and deblur is an interesting portion of the pipeline. The OTU clustering methods, dnaclust, vsearch and the ASV method deblur do not have any documented evidence of being shown to work with Pacbio reads and obtain consistent comparable

results. Did the authors have to modify these methods to work with Pacbio datasets and if the authors did have to make changes, it would be beneficial to the user to understand their implementation.
We thank the reviewer in expressing this important concern. We did not modified deblur in any ways to make it capable of processing PacBio CCS reads, but the very nature of long reads probably makes this type of data impractical for performing ASV based analysis.

In consequence, we improved the manuscript on two elements regarding the analysis of PacBio data:

1) We added a PacBio mock community dataset to compare our pipeline's output against what is expected. The analysis of the mock community made us realize that the vsearch and dnaclust parameters seemed to be adequate, but that the deblur –min-reads 2 or 3 (reject reads having an abundance less than 2 or 3 respectively). Argument ended up in the rejection of the majority of the reads in the dataset, inevitably affecting downstream diversity metrics. We did not report ASV results for –min-reads lower than 3 as we ended up with 4 ASVs only, which didn't make sense to pursue. However, setting the –min-reads parameter to 1, ended up in keeping the majority of reads for ASV generation. However, this resulted in a situation where all ASVs had an abundance of 1 (Table 2) with almost no ASVs overlapping across samples. In consequence, the downstream diversity metrics alpha does not really have any useful meaning as shown in figure S6b). Taxonomic summary obtain with deblur was highly similar to the ones obtained with dnaclust and vsearch.

2) We integrated an additional deblur analysis with the `–min-reads 1` parameter (in addition to the original `–min-reads 3` parameter) for the PacBio oral microbiome dataset (Table 2 and figure S7). The results obtained with –min-reads 1, but not –min-reads 3 (mr1 in the figure) shows similar taxonomic profiles as the ones obtained with dnaclust and vsearch. However, because both parameters distorts the resulting ASV abundance profiles so much - very few ASV with `–min-reads 3` and high abundance of singlets with `–min-reads 1` – resulting alpha and beta diversity values are in the end meaningless as shown in figure S7b) and c) (and also figure 6b).

Consequently, we believe that ASVs are not the best fit to appropriately to analyze long PacBio amplicon reads and we lean toward using a clustering workflow to analyse long PacBio amplicons. That said, performing an ASV analysis were all ASVs are kept (including the singlets) might be appropriate for taxonomic profiles (but not alpha and beta diversity) assessment. We also investigated the use of more lenient parameters `--mean-error <float> --indel-prob <float>` with very little improvement on the number of singlets.

Lines 215-228, 354-358, and 267-375 in the revised manuscript discuss these matters.

Additionally, please describe the alignment step followed for Pacbio dataset.
We added description for the alignment step (step 11, line 567, in the methods of the revised manuscript) when data type is long PacBio reads. We also include details on this step in the user guide (additional file 4).

The available end to end solutions such as QIIME2, Mothur, DADA2 + Phyloseq are designed to be both interactive as well as to be operated well on a HPC environment, the discussed pipeline here provides no significant advantage for users without discussing comparisons in run time such as core hours, accuracy in results across the different publicly available pipelines. An interactive environment is essential both in a production environment as well as for carrying out batch analysis as these steps allows the researcher to understand their dataset which is the most important and significant time

consuming portion of obtaining accurate analysis of marker gene dataset. The authors show the results of a mock community which contains both even and staggered community. A comparison of the different pipelines with Amplicon tagger will provide the user a confidence of the tool if they display similar results across the tools.
We agree with the reviewer's comments and therefore added an additional set of mock community data in the revised manuscript. We now report and discuss two mock community short amplicon data set (now figure 2) analyzed with AmpliconTagger (i.e. deblur, dnaclust and vsearch) and also with one qiime2-deblur, one qiime2-dada2 and another one with qiime2-vsearch workflows. This new data is reported and discussed in the revised version of the manuscript at lines 288-306. As an additional note, we replaced the original mock community dataset with another one (table 1) as the original one showed high variation in one of the replicate, which we feared could introduce confusion in readers.
Regarding the comment about interactive environment, our pipeline supports the generation of jobs for interactive execution, by specifying the `-j batch` argument instead of `-j slurm`. Doing so, instructs the pipeline to generate jobs free of all the SLURM syntax and can be executed directly from the command line.

The use of DUK software, an unpublished method may not be the best choice. If the authors could use a published contaminant removal tool for this step by replacing DUK software will make the entire pipeline more robust.
We agree that the DUK software is an objectively old, unpublished and unmaintained software. We therefore replaced DUK with BBDUK from the BBTOOLS software suite. While BBTOOLS has not been published yet in a peer reviewed journal, it is exhaustively documented and actively maintained and used by/at the DOE – Joint Genome Institute, a reference institution in bioinformatics of microbial ecology. BBDUK is also running faster than DUK. We hope this replacement will satisfy the reviewer.

Minor: The docker contains PICRUSt as a tool without providing much detail on their website or in the article if any portion of PICRUSt is implemented.
We thank the reviewer for pointing this: all traces of PICRUSt have been removed from the docker image, pipeline repository and website.
