# Supplementary material for: Systematic processing of ribosomal RNA gene amplicon sequencing data
Source: Gigascience. 2019 Dec 9;8(12):giz146. doi: 10.1093/gigascience/giz146 (PMC6901069; doi:10.1093/gigascience/giz146)
Supplement: giz146_GIGA-D-19-00228_Revision_1 [file giz146_giga-d-19-00228_revision_1.pdf]

GigaScience

Systematic processing of rRNA gene amplicon sequencing data

--Manuscript Draft--

Manuscript Number:	GIGA-D-19-00228R1
Full Title:	Systematic processing of rRNA gene amplicon sequencing data
Article Type:	Research
Funding Information:	
Abstract:	With the advent of high throughput sequencing, microbiology is increasingly becoming a data intensive field of science. Because of its low cost, robust databases and established bioinformatic workflows, sequencing of 16S/18S/ITS rRNA gene amplicons, which provides a marker of choice for phylogenetic studies, has become ubiquitous and has grown into the backbone of modern microbial ecology. Many established end-to-end bioinformatic pipelines are available to perform short amplicon sequence data analysis and have proven to be central for advancing the field of microbial ecology. These pipelines have been partly written for a general audience, which is arguably a main reason for their widespread adoption. However, few options exist for more specialized users that are experienced in Linux-based systems and high performance computing (HPC) environments. For such an audience, existing pipelines can be limiting to fully leverage modern HPC capabilities and perform tweaking and optimization operations. Moreover, a wealth of stand-alone software packages that perform specific targeted bioinformatic tasks are increasingly accessible through code repositories and scientific publications and finding a way to easily integrate these applications in a pipeline is critical in fast-paced evolution of bioinformatic methodologies. Here we describe AmpliconTagger, a short rRNA marker gene amplicon pipeline coded in a python framework that enables fine tuning and integration of virtually any potential rRNA gene amplicon bioinformatic procedure. It is designed to work within an HPC environment, supporting a complex network of job-dependencies with a smart-restart mechanism in case of job failure or parameter modifications. As proof of concept, we present end results obtained with AmpliconTagger using 16S, 18S, ITS rRNA short gene amplicons and PacBio long read amplicon data types as input. Using a selection of published algorithms for generating Operational Taxonomic Units (OTUs) and Amplicon Sequence Variants (ASVs) and for computing downstream taxonomic summaries and diversity metrics, we demonstrate the performance and versatility of our pipeline for systematic analyses of amplicon sequence data.
Corresponding Author:	Julien Tremblay<br><br>CANADA
Corresponding Author Secondary Information:	
Corresponding Author's Institution:	
Corresponding Author's Secondary Institution:	
First Author:	Julien Tremblay
First Author Secondary Information:	
Order of Authors:	Julien Tremblay
	Etienne Yergeau
Order of Authors Secondary Information:	
Response to Reviewers:	Reviewer #1: I thank the authors for addressing my comments and making changes to their manuscript. I also thank the authors for maintaining the list of commands, their SLURM scripts and the documentation of the tool. I believe the manuscript has benefited from these changes. However, please review the below responses.<br><br>Analysis of Mock communities:

Powered by Editorial Manager® and ProduXion Manager® from Aries Systems Corporation

As stated in lines 139 - 143 the analysis steps for each data analysis is provided in Additional file 2 and 3 with explicitly stating that QIIME2 commands are contained within the Additional file 3. While I refer to this file, I notice that the file (Additional file 3) is labeled as, "Qiime2 commands used to process the indoor microbiome data" which is not the same as the mock community dataset.

Answer: We thank the reviewer for pointing this inconsistency. We indeed uploaded the wrong file during the submission process. The file the reviewer examined is actually the "old" additional file 3 from the previous manuscript version. We made sure to provide the correct additional file 3 in the revised manuscript and we apologize for this.

Assuming Additional file 3 is the file with the parameters the authors used for processing the Mock community, I do not see a constant use of the same trimming parameters between different methods. For example: QIIME-deblur method allows reads to be 249 bp whereas QIIME-DADA2 method trims the reads to 150 bp. If these trimming parameters were followed, the discrepancy described in the lines, 154-160: of overestimated abundance of Staphylococcus is quite possible. Why are not all the trimming options been kept consistent?

Answer: The reason for this is because DADA2 does not allow to use merged paired-end amplicons. So basically, reads have to be submitted separately as R1 and R2 to DADA2, hence the different read length of 150 bp compared to the one used in Deblur which accepts merged amplicon reads.

I do notice the authors mention, "dada2 kept rejecting the staggered synthetic community library, I had to lower the parameters to make it accepts all the libraries".
If the authors could not get the QIIME2-DADA implementation work, can they test this against the stand-alone DADA2 version where there is a larger flexibility of choosing options than considered here.

Answer: We investigated why exactly our QIIME2-DADA2 workflow behaved that way and we found the correct parameters to make it work accordingly (as included in additional
file 3). We did our best to run DADA2 with parameters similar as much as we could to what was done in our pipeline.

There seems to be an inconsistency with respect to assigning taxonomy. They assign taxonomy using 2 different sets of Silva R128 training sets, one for AmpliconTagger and one for QIIME2 results. Why was this choice made?

Answer: Like stated above the reason to use QIIME2 here was to process the mock community data completely with three QIIME2 implementations which includes using the QIIME2 provided database and see if it compares with end results obtained by AmpliconTagger.

The authors in the paper state that, "We wish to emphasize that we do not present our methodology as a gold standard but rather a blueprint of an end-to-end open source working modular pipeline". This statement seems to be contrary to the decisions followed for analysis.

Answer: In this manuscript we showcase the capability of our pipeline by exposing end results of several datasets covering many data types processed with 4 different workflows (DADA2, Deblur, VSEARCH and DNACLUST), we believe that this objectively shows that our pipeline indeed provides a blueprint of an end-to-end open source flexible/modular pipeline. At the reviewer's excellent suggestion in the previous round of revisions, we added an extensive documentation of our pipeline so others could implement their methodology as they see fit. Of course we think our methodology provides accurate results for the interpretation of amplicon sequence data, but that does not mean that we present our methodology as a gold standard and nowhere in the manuscript do we suggest that.

Figure 1b needs to be labeled to describe which portion of data was run in Amplicon tagger vs QIIME2.

Powered by Editorial Manager® and ProduXion Manager® from Aries Systems Corporation

Answer: OK, this has been addressed in the revised manuscript at lines 756-757.
Figure 1c: This figure is very difficult to read. Please re-order this figure especially the center panel where the dnaclust is placed above qiime2-dada2 results. They are not the same comparisons.

Answer: OK, this has been addressed in the revised manuscript. We believe figure 1 is now clearer.

Dnaclust comparison from a 3rd party is missing. DADA2 is missing from the tool list in Amplicontagger.

Answer: To the best of our knowledge, DNACLUST implementation from a third party pipeline is not available which explains its absence in the 3rd party validation section. We added this important fact in the manuscript at lines 154-155. DADA2 has been added from the tool list in the revised manuscript (line 692).

Also dada2 analysis has been included only for the mock communities and is missing from their analysis of other datasets. Why were these choices made?

Answer: Since DADA2 was not implemented in AmpliconTagger, in the manuscripts's previous iteration we did not use it for datasets other than the mock community. The use of DADA2 in the mock community has strictly been done to compare results of AmpliconTagger against results of the same data entirely processed with an established method – in this case QIIME2 with either DADA2, VSEARCH or Deblur as ASV/OTU generation method.
However, in line with the below comments from the reviewer, we implemented DADA2 in AmpliconTagger and now provide analyses done with DADA2 for all datasets investigated in the manuscript.

From Figure 1d) there is a large difference in the results for the staggered community results observed for AmpliconTagger vsearch v/s QIIME2-vsearch. Their vsearch implementation provides a result of 9 OTU's vs 25 - 30 OTU's in the QIIME2-vsearch results. Were same parameters used? Did they use the same version of vsearch in the comparisons?
Additionally, from figure 1D: Did the authors obtain a greater number of ASV's than in the mock community? This usually points to not having similar trimming parameters across different methods used.

Answer: The parameters in QIIME2-VSEARCH were modified so that they could as close as possible to what is used in AmpliconTagger-VSEARCH. The actual parameter that was critical and that we failed to properly include in the QIIME2 analyses of the previous iteration was the minimum reads cutoff per ASV or OTU which was of 25 for all AmpliconTagger's short reads analyses, but was unfortunately not applied for the QIIME2 workflows. This has been corrected in the revised version of the manuscript. We re-ran all analyses and the number of ASVs for the mock community analyses are now much more consistent.

Table 3 provides the correlations between the different tested methods, however it would have been interesting to compare the AmpliconTagger vs QIIME2 results for deblur, vsearch. I think addition of this statistic will provide more useful information to the user of their tool than the current implementation.
Answer: OK we added this information in Table S3 in the revised manuscript and added discussion elements at lines 163-167.

Additionally, table 3 does not list the Mantel statistics for all the datasets. Why?

Answer: Table 3 missed the comparison from the QIIME2 processed results. As stated in the answer above, this information is now available in Table S3. Table 3 of the current iteration of the manuscript includes all other non-QIIME2 comparisons.

As a user and a reviewer, while I look at a tool and its performance: I look for the following parameters to obtain confidence on the method,

Powered by Editorial Manager® and ProduXion Manager® from Aries Systems Corporation

	i. Same trimming parameters across all comparisons,<br>ii. Same version of a method across comparisons,<br>iii. Same taxonomic assignment databases.<br>iv. Consistent results across different methods.<br><br>From following the results of the mock community analysis, I do not see their decisions in the mock community analysis satisfying the above criteria.<br><br>The discrepancies in the above steps makes it difficult to obtain confidence in the comparison carried out here.<br><br>Answer: This comment is in line with the reviewer's previous comments regarding this matter and we wish to clarify our reasoning (briefly stated in the manuscript at lines 130-134).<br>1-Following one of the reviewers' comment on the previous iteration of our manuscript, our objective with QIIME2 was to compare the end results of amplicon data from our method against an established pipeline, in this case QIIME2. In that context, the choice to use different parameters was unavoidable as the packages or scripts used to process the data (other than VSEARCH, DADA2 and Deblur) are inherently different in AmpliconTagger vs QIIME2. Besides, we wanted to see how AmpliconTagger's results compared against QIIME2 ran with standard parameters (i.e. identical or very close to what is described in the QIIME2 help web page) with three OTU/ASV generation methods supported in QIIME2. These parameters are probably what many users will use in "production" settings and confidently assessing that AmpliconTagger gives similar results to what is obtain with QIIME2 under these "standard" circumstances supports our method to be valid.<br>2-As stated in point 1) above, comparing our method with QIIME2 executed with identical parameters is impossible as our pipeline's methods are in the end different enough from the ones that QIIME2 implements that it is not possible to accomplish. For instance trimming and reads filtering parameters is performed differently in AmpliconTagger (tagsQC.pl) compared with what is done in QIIME2. As another example, there are no reads contaminants filtering steps in QIIME2 in contrast to our pipeline.<br>3-Regarding taxonomic database, we deliberately choose a database provided on QIIME2's website so that again we could compare analyses with the ones done entirely by QIIME2 – which includes using QIIME2's stock database. In that case, however, we also deliberately choose QIIME2's Silva 128 release (the same release that we used), but again, the methods (classifier software) and the way the training sets are built from the Silva 128 release are different.<br>4-We strongly believe that we demonstrated the results obtained with QIIME2 vs AmpliconTagger to be comparable and consistent. Moreover this consistency is robust to the aforementioned differences between the two methods (AmpliconTagger vs Qiime2).<br><br>Pacbio long reads analysis:<br><br>While the taxonomic profiles obtained show good results across all the comparisons, i.e., dnaclust, vsearch and deblur the number of ASV's for deblur does not inspire confidence in being available as a method for Pacbio analysis. I would suggest the author's disable deblur as an option for Pacbio due to the large discrepancy of the number of ASV's.<br><br>Answer: After examining more closely the output of the PacBio mock community data, we realized that CCS reads were provided in both 53' and 3'5' orientation which affected downstream results. We entirely reprocessed the PacBio data mock community data from scratch by re-orienting the reads in the correct orientation. Moreover, we refined our methods and kept reads that had length between 1200 and 1600 bases which rejected many shorted and longer sequences that were probably artefacts. In consequence, the end results are much cleaner for all the 4 methods (Dnaclust, Vsearch, DADA2 and Deblur). Our results show that the results Deblur provides are now in line with what is generated by the other 3 packages and we wish to keep Deblur support in our pipeline. We also reprocessed the PacBio oral microbiome data, but in that case reads were already oriented in the 5'->3' direction.

Powered by Editorial Manager® and ProduXion Manager® from Aries Systems Corporation

Of all the above methods, none of them have been specifically designed for long reads as all of them were designed for short read data. I think this tool will hugely benefit from including DADA2 due to: a) It will provide users 2 OTU methods and 2 ASV methods as options; b) DADA2 is the only tool which has a defined Pacbio analysis.

The pre-print for DADA2 Pacbio:
https://www.biorxiv.org/content/10.1101/392332v2.abstract.

If the authors are keenly interested to provide Pacbio processing, I would strongly suggest them to implement DADA2 in their tool which will give them a ASV method designed for Pacbio data analysis.

Answer: We thank the reviewer for this excellent suggestion. We added support for DADA2 PacBio data processing in our pipeline.

Figure S5:c has SNV's instead of ASV. Please fix this.
Answer: Done.

Minor comments:

Qiime2 is written as QIIME2.
Answer:Corrected.

dada2 is written as DADA2.
Answer: Corrected.

Additional Feedback on missing reviewer 2:

Getting external advice on reviewer 2's comments they don't feel you have sufficiently addressed the definition of "advanced customization" in mothur in lines 80-82 as this is only a quick comment on how Qiime offers limited options compared to R. As the reviewer says, mothur offers this functionality with many more options. How is AmpliconTagger advantageous over this?

Answer: We believe that the sentence at line 80-82 is a good example highlighting that many bioinformatics software packages have a wide range of options and that only a selection of these options are often included in pipeline wrappers. Moreover, nowhere do we suggest that the QIIME offers limited options over R, because we think this is not a relevant comparison: R is a programming language and QIIME is a pipeline supporting the execution of a range of software packages written in different languages. Regarding the reviewer's comment, we attentively screened Mothur's documentation and to the best of knowledge, it does not support DADA2 or ASV generation methods. In contrast to QIIME, Mothur is more of a stand-alone program that re-implements relevant microbial ecology algorithms into a stand-alone software. In that regard, it is true that our comment of limited advanced customization was not entirely appropriate in the case of Mothur – we therefore rephrased our statement at lines 80-82 of the revised manuscript.
Our pipeline is targeting an audience that is comfortable in code scripting, Linux and HPC environments (stated at lines 33-38 and 472-474) and as such it is advantageous over other pipelines because it allows, with some python scripting, to add literally any parameter to any software package (thoroughly explained in the User guide /Additional file 4).

If the primary objective of this report was to showcase AmpliconTagger, to do this more work is probably required to better demonstrate and discuss what advantage AmpliconTagger has over existing pipeline tools like Snakemake, Cromwell, or Nextflow for HPC pipelines, or even Nephele which offers easy interfaces and tools for microbiome analyses, without the need to install a whole system on a web interface.

Answer: The tools that the reviewer is referring to : Snakemake, Nextflow, Cromwell and Nextflow are declarative workflow description languages such as CWL (Common Workflow Language) or WDL (Workflow Description Language) are dedicated to providing a customizable framework to build bioinformatics pipelines. Such solutions

Powered by Editorial Manager® and ProduXion Manager® from Aries Systems Corporation

	are flexible and can help in pipeline implementation but do not offer the same level of features (for instance smart job restart/regeneration mechanism) and completely functional and modular 16S amplicon pipeline that was built and ready for production (our manuscript). We appreciate the reviewer's interest in comparing our method to the ones he mentions, but that would be an entirely separate manuscript in itself (i.e. comparing GenPipes with Snakemake, Nextflow, etc.). As such re-implementing our pipeline using these tools is outside the scoop of the work present here and would not add any benefits to the readers. Again, in this work, we present an exhaustive description and demonstration (including all the code) of a modular and flexible pipeline able to process any types of amplicon data and we provide all the results to back these claims.<br>Nephele is web (graphical) interface supporting a limited implementation of 16S and ITS data types processing. The authors realize that while this tool may be convenient in some situation, it does not in any way compare with the flexibility that AmpliconTagger provides with its modular nature and command-line –based execution. We are not sure what the reviewer is referring to when he states that we need to install a whole system on a web interface: AmpliconTagger is run directly from the command line on a Linux-based system.
Additional Information:	
Question	Response
Are you submitting this manuscript to a special series or article collection?	No
**Experimental design and statistics**<br><br>Full details of the experimental design and statistical methods used should be given in the Methods section, as detailed in our Minimum Standards Reporting Checklist. Information essential to interpreting the data presented should be made available in the figure legends.<br><br>Have you included all the information requested in your manuscript?	Yes
**Resources**<br><br>A description of all resources used, including antibodies, cell lines, animals and software tools, with enough information to allow them to be uniquely identified, should be included in the Methods section. Authors are strongly encouraged to cite Research Resource Identifiers (RRIDs) for antibodies, model organisms and tools, where possible.<br><br>Have you included the information requested as detailed in our Minimum	Yes

Standards Reporting Checklist?	
**Availability of data and materials**<br><br>All datasets and code on which the conclusions of the paper rely must be either included in your submission or deposited in publicly available repositories (where available and ethically appropriate), referencing such data using a unique identifier in the references and in the "Availability of Data and Materials" section of your manuscript.<br><br>Have you have met the above requirement as detailed in our Minimum Standards Reporting Checklist?	Yes

Manuscript
Click here to access/download;Manuscript;AmpliconTagger_paper_rev4_2019
Click here to view linked References

Systematic processing of rRNA gene amplicon sequencing data.

Julien Tremblay[1#] and Etienne Yergeau[2]

[1]: Energy Mining and Environment, National Research Council Canada, Montreal, QC, Canada H4P-2R2

[2]: Centre INRS-Institut Armand-Frappier, Institut national de la recherche scientifique, 531 Boul. des Prairies, Laval, QC, Canada, H7V-1B7

[#]: Corresponding author - julien.tremblay@nrc-cnrc.gc.ca

ORCID IDs:

Julien Tremblay : 0000-0002-6085-3481; Etienne Yergeau: 0000-0002-7112-3425

Running title: AmpliconTagger pipeline

Abstract

With the advent of high throughput sequencing, microbiology is increasingly becoming a data intensive field of science. Because of its low cost, robust databases and established bioinformatic workflows, sequencing of 16S/18S/ITS rRNA gene amplicons, which provides a marker of choice for phylogenetic studies, has become ubiquitous and has grown into the backbone of modern microbial ecology.

Many established end-to-end bioinformatic pipelines are available to perform short amplicon sequence data analysis and have proven to be central for advancing the field of microbial ecology. These pipelines have been partly written for a general audience, which is arguably a main reason for their widespread adoption. However, few options exist for more specialized users that are experienced in code scripting, Linux-based systems and high performance computing (HPC) environments. For such an audience, existing pipelines can be limiting to fully leverage modern HPC capabilities and perform tweaking and optimization operations. Moreover, a wealth of stand-alone software packages that perform specific targeted bioinformatic tasks are increasingly accessible through code repositories and scientific publications and finding a way to easily integrate these applications in a pipeline is critical in fast-paced evolution of bioinformatic methodologies.

Here we describe AmpliconTagger, a short rRNA marker gene amplicon pipeline coded in a python framework that enables fine tuning and integration of virtually any potential

rRNA gene amplicon bioinformatic procedure. It is designed to work within an HPC environment, supporting a complex network of job-dependencies with a smart-restart mechanism in case of job failure or parameter modifications.

As proof of concept, we present end results obtained with AmpliconTagger using 16S, 18S, ITS rRNA short gene amplicons and PacBio long read amplicon data types as input. Using a selection of published algorithms for generating Operational Taxonomic Units (OTUs) and Amplicon Sequence Variants (ASVs) and for computing downstream taxonomic summaries and diversity metrics, we demonstrate the performance and versatility of our pipeline for systematic analyses of amplicon sequence data.

Background

High throughput sequencing of amplicons of fragments of the 16S, 18S and ITS rRNA marker genes has grown into a cornerstone of microbial ecology research activities. Amplicon sequencing is now massively widespread and has been used in large research initiatives such as the NIH funded Human Microbiome Project[1–3] and Earth Microbiome Project[4].

Despite this, it is still objectively difficult to adequately analyze data[5,6]. Initiatives to provide graphical user interface-based applications have been reported[7–9]. These types of interfaces, by their fundamental nature, are not prone for systematic analysis in a production context involving the processing of high data loads of multiple projects

simultaneously.

Efforts to integrate bioinformatic pipelines as a standard tool to establish microbiome profiles in food safety and energy settings are increasingly being reported[10,11] and 16S rRNA marker genes studies are increasingly being reported to be relevant to complement traditional methods in a clinical context[12–16].

The bioinformatic landscape for processing short marker gene amplicon sequencing data contains a wide array of solutions and is dominated by a few open-source popular pipelines such as QIIME[17] and Mothur[18]. In order to execute, these pipelines usually require users to use streamlined or pre-defined steps with limited ability for advanced customization or possibility to use specific unsupported third party software packages. For instance DADA2[19], a package for ASV generation is not available in Mothur, but is implemented in QIIME2[20] with only a subset of input parameters accessible to the user compared to much more from the DADA2 original R package. These efforts were and are continuing to be immensely important in democratizing rRNA amplicon data processing, making it possible for uninitiated Linux users to be able to perform their own data analysis. As such, these pipelines are arguably targeting investigators unfamiliar with computer coding and command line execution. This kind of enclosed setting, however, can become a limiting factor for the types of users who are both proficient at code scripting and comfortable in a Linux/command-line environment. Moreover, bioinformatic methods are constantly evolving so that production bioinformatic pipelines need to be adapted and modified on a regular basis to properly integrate newly

published bioinformatic packages.

Bioinformatic pipelines are intrinsically complex with up to hundreds of steps depending on the input sequencing data type with some of these steps needing large compute resources to properly execute. It is critical to introduce robust and flexible ways of systematically processing metagenomic sequencing data types (*i.e.* mainly amplicons and shotgun) in order to increase their adoption in the aforementioned settings. The GenPipes workflow management system, including an implementation of a QIIME-based 16S rRNA amplicon pipeline, was recently published[21]. Here, as a proof of concept, we leveraged GenPipes's capabilities to build AmpliconTagger, a versatile bioinformatic pipeline managing job generation, submission, dependency and smart restart that can process any type of gene amplicon sequencing data (16S, 18S, ITS rRNA genes and other custom marker or functional genes) of various sequencing configurations integrating multiple bioinformatic packages. We validated our pipeline with three QIIME2 workflows (VSEARCH, Deblur and DADA2) using two mock communities datasets for which we know the exact community composition. We then present microbiome profiling results from published short (MiSeq) and long (PacBio) amplicons sequencing datasets using two Operational Taxonomic Units (OTUs) and one Amplicon Sequence Variants (ASVs) algorithms. We also present detailed information on our methodology so that it can be promptly used, adapted and improved by others.

Data Description

In order to document various aspects of AmpliconTagger, we processed seven published and publicly available datasets of rRNA amplicon sequencing data of various sequencing configuration, targeting various marker genes and regions (Table 1). We aimed to include datasets from a variety of ecosystems: indoor, human gut and oral, soil and water. We also included a novel dataset consisting of a commercial mock community.

Analyses

Experimental design

Each of the datasets described in table 1 were processed into the AmpliconTagger pipeline which contains from 91 to 94 jobs depending on the OTU/ASV generation algorithm used and on sequencing configuration (paired vs single end sequencing data). All datasets were processed following a common core of quality filtering procedures, but submitted to two different methods of OTUs generation (Additional file 1 - Fig. S1) (Vsearch[29] and Dnaclust[30]) and two ASVs method (Deblur[31] and DADA2). In addition, short amplicons mock community datasets were also entirely processed in QIIME2 using a VSEARCH, Deblur and DADA2 workflow, in order to compare our pipeline against a third-party reference method. We also present and discuss community profiling results of long PacBio amplicons using a mock community and a published oral microbiome sequencing dataset (Table 1). More in-depth analyses of common microbial ecology metrics were assessed for each project for each OTU/ASV generation method and are available in Additional file 1. Although we do

present some high level analysis of ASVs vs OTUs end results, this study primarily aims at demonstrating the modularity and methodology implemented in AmpliconTagger and less at performing an exhaustive comparison of the OTU/ASV-generating packages that we used. The complete data processing description of AmpliconTagger is described in the methods section and the complete commands of each job of each data analysis run is available in additional file 2 (AmpliconTagger command traces) and 3 (QIIME2 command traces for the 16S V4 region mock community). An exhaustive user guide is available in additional file 4.

Validation with mock communities and comparison with third party reference pipeline.
In order to validate AmpliconTagger, we processed two defined mock communities, one with even concentrations of 20 bacterial strains (Table S1) and one with staggered concentrations of 9 genomes (Table S2). We obtained community profiling results of each mock sample using AmpliconTagger (VSEARCH, DNACLUST, Deblur and DADA2) and compared them with the end results of the same sequencing libraries, but entirely processed with QIIME2 using VSEARCH, Deblur and DADA2 workflows (*i.e.* DNACLUST is not implemented in QIIME2). Taxonomic profiles are highly similar across all tested methods (Fig. 1a and 1b) and minor differences are probably caused by the two different Silva R128 training sets used by AmpliconTagger (100% identity sequences) vs QIIME2 (database, clustered at 99% identity). Notably, QIIME2 workflows identified a major taxa as being assigned to *Clostridiales*;Other and *Enterobacteriales*;Others while AmpliconTagger classified them instead as *Lachnoclostridium* and *Pantoea* (Fig. 1b). Regardless of the methods used, all samples

clustered similarly in beta-diversity ordinations (Fig. 1c) and show relatively similar alpha diversity (Observed OTUs/ASVs index) values (Fig. 1d). We computed the Mantel *r* statistic to assess for correlation between weighted UniFrac and Bray-Curtis distance matrices between all seven tested methods (Table S3), which generally shows high correlation (> 0.8) for most comparisons except for the ones were the QIIME2-DADA2 data was involved.

Performance

We compiled compute resources consumed for each of the datasets considered for this study (Fig. 2). The common core of AmpliconTagger consists of steps 1 to 6 as detailed below in the methods section. The indoor microbiome, Lake Michigan and AAD studies consumed similar amounts of core•hours (22.7, 20.2 and 26.6 core•hours respectively) while the mock community, oral microbiome and rhizosphere microbiome transplant studies took significantly less resources with respective values of 0.53, 2.2 and 5.2 core•hours. OTU/ASV generation jobs consumed the most resources for the oral microbiome (PacBio long reads) project followed by the indoor microbiome data which again is the dataset containing both the highest number of (short) reads and base-pairs. For the oral microbiome data, which had a low data input, but long read lengths, DADA2 took the longest time to generate ASVs with downstream steps completing quickly because of the low number of ASVs generated compared to other OTUs or ASVs generation methods (Table 2). Generally, OTU/ASV generation and their downstream steps consumed more resources than the common core steps.

Microbial ecology metrics obtained for each dataset.

Globally, ecological patterns were similar for the four tested methods for all projects (Table 3 and Additional file 1), except for the two 16S PacBio data types (oral microbiome and PacBio mock data). The numbers of ASVs and OTUs obtained for a given project were fairly similar for short amplicon data types, except for the indoor microbiome data which yielded 27,391 Deblur ASVs, 32,647 DADA2 ASVs, 18,340 DNACLUST OTUs and 14,341 VSEARCH OTUs (Table 2). In contrast, the number of Deblur ASVs obtained with the oral microbiome data was of 199,780 compared to 6,535, 77,305 and 69,578 for DADA2, DNACLUST and VSEARCH OTUs respectively. The total number of reads included in these OTU/ASV was of 219,423, 6,535, 77,305 and 523,444 for Deblur, DADA2, DNACLUST and VSEARCH respectively. In general the amounts of generated ASVs/OTUs were similar, but ASVs represented less total reads.

In order to highlight differences between the four tested OTU/ASV generation methods, we compared their taxonomic summaries, alpha- and beta- diversities. These results are included in figures S2 to S7 (Additional file 1) and for clarity and practicality purposes, we narrowed sample selection for each project to a subset of experimental variables. We also computed the Mantel *r* statistic to assess for correlation between weighted UniFrac and Bray-Curtis distance matrices between all four tested methods (Table 3). Overall, and consistent with results reported in additional file 1, these results show that weighted UniFrac and Bray-Curtis distances/dissimilarities obtained with all four tested methods are quite similar with *r* statistic values greater than 0.9 for the

indoor microbiome, AAD and Lake Michigan data types. The ITS data (rhizosphere transplant) showed low *r* statistics between 0.4 and 0.6 between the weighted UniFrac matrices. However, Mantel tests between Bray-Curtis dissimilarity matrices of all four OTU/ASV ITS data types gave *r* statistics higher than 0.9 for all comparisons. Weighted UniFrac distances of all four methods were similar for the Mock community with *r* statistics values all greater than 0.9. However, Bray-Curtis dissimilarity matrices were more divergent with *r* statistics values of 0.580 (Deblur vs VSEARCH), 0.580 (VSEARCH vs DADA2) and 0.724 (DNACLUST vs VSEARCH). Overall, this indicates that the type of distance metric used had a considerable impact on the microbial population structure assessment.

Validation of procedures with long PacBio reads datatype.

Using PacBio full length amplicon sequencing for microbial community profiling is increasingly making inroads to complement population characterization based on short amplicon sequencing, but bioinformatics procedures are objectively recent and not as mainstream as they are with short length amplicon sequencing data. We therefore validated our long 16S reads processing methodology with a PacBio mock community library [32] (Table S1). Taxonomic profiles of all four tested methods (VSEARCH, DNACLUST, DADA2 and Deblur) are generally similar with some minor differences in low abundant taxa (Fig. S6a). However, more important discrepancies are actually observed between expected taxa and what was obtained with the actual sequencing libraries (Fig. S6b). Alpha diversity metrics were computed and observed ASVs ranked near the expected number of 23 ASVs whereas observed OTUs were higher than the expected value: 60.6 +/- 5.9 for DNACLUST and 172.4 +/- 16.2 for VSEARCH. Mantel *r*

statistics between DNACLUST and VSEARCH were relatively high (0.851 weighted UniFrac and 0.844 for Bray-Curtis), but weak for the comparisons involving ASV data. For the PacBio oral microbiome data, Mantel *r* statistics computed between the UniFrac distance matrices of all four data types (Table 3) showed strong values > 0.9 for DNACLUST vs VSEARCH, DNACLUST vs DADA2 and VSEARCH vs DADA2, indicating relatively high similarity between all ASVs and OTUs distance matrices when phylogenetic distance is factored in the distance computation. Bray-Curtis matrices, however, showed lower correlations between DNACLUST vs DADA2 (0.415), VSEARCH vs DADA2 (0.436), while DNACLUST vs VSEARCH (0.928) distances were highly correlated. Comparisons involving Deblur data returned low or negative values.

Discussion

Backbone for rRNA sequence processing methodology.

The primary objective of this report was to showcase AmpliconTagger, a highly modular HPC oriented pipeline geared for performing bioinformatic analyses of rRNA amplicon data. To facilitate implementation and testing, we shared a docker repository of a CentOS-7 image with a fully working implementation of AmpliconTagger which includes data of 20 sequencing libraries from the AAD study, all the databases, training sets and fully detailed commands of each job of each step of our pipeline. Our workflow also relies on a set of perl scripts and libraries labeled nrc_tools for which the complete code is also available in a code repository. We wish to emphasize that we do not present our methodology as a gold standard, but rather as a blueprint of an end-to-end open source

working modular pipeline that is able to incorporate virtually any in-house scripts or third-party packages (see Additional file 4 for more details). We also recognize that some steps in our workflow could be improved and even be replaced by other potentially more efficient software or method, which is highly facilitated by the open modular nature of the pipeline.

Resources and time consumption.

With bioinformatics entering many spheres of research and service fields, there is a need for flexible, scalable and robust methods to systematically analyze high-throughput nucleic acid sequencing data of all types. Here we presented in details our gene amplicons production workflow with performance metrics and actual results from published rRNA marker gene datasets. We executed our workflow using two OTU generation methods - VSEARCH and DNACLUST and two ASV generation method – Deblur and DADA2. We included VSEARCH because it represents the open source version of the popular USEARCH closed-source software. As such, VSEARCH is increasingly being used by the research community as a suitable replacement for USEARCH. Our group started to use DNACLUST as a suitable open-source alternative to replace USEARCH some years ago and since then, we have processed many rRNA marker gene sequencing datasets using this package and it is the reason for its inclusion in this study. The goal of the present study was not necessarily to benchmark these four OTU/ASV generation methods, but rather to show the modular nature of the pipeline and offer useful metrics of what microbiome profiling results and resource consumption can be expected from using these software in the context of our whole

workflow methodology.

While short amplicon sequence data processing is not that demanding from a computational resource point of view, a complete working pipeline requires many steps in order to get from the raw data to key end-results. Here we show that quality control and fastq preprocessing steps of sequencing data prior to OTU/ASV generation consumed the most core•hours for datasets that had an appreciable number of reads (indoor microbiome, Lake Michigan and AAD). The rhizosphere transplant project had comparable number of reads, but possibly because of short ITS read length (once paired-end assembled), jobs relying on Qscore metrics like quality filtering and quality score profile compilation quickly completed (Fig. 2). For downstream steps, which correspond to the OTU/ASV generation to up to the pipeline completion, the indoor microbiome (single-end 150 bp reads) and oral microbiome (single-end ~1,470 bp reads) consumed the most resources, the former, because of the high raw data load (16.8 Gb) and the latter because of its long read lengths. In terms of memory consumption, the indoor microbiome project, again because of its high amount of raw bp, consumed significantly more RAM than the other projects.

Validation with mock communities.

Comparisons of mock community profiling results obtained by our pipeline with the ones obtained with a QIIME2 -VSEARCH -Deblur or -DADA2 workflow were highly concordant (Fig. 1). All results deriving from either AmpliconTagger or QIIME2 were essentially similar and consistent with the expected taxonomy. The staggered

community (Fig. 1b) gave almost identical community structure patterns, but slight differences in taxonomic assignments were observed for the most abundant taxon which was supposed to be a *Lachnoclostridium* (Fig. 1b - expected panel), but ended up being classified as a *Lachnocolstridium 10* by AmpliconTagger (DNACLUST, VSEARCH, DADA2 and Deblur) and an undefined genus belonging to the *Clostridiales* order for the QIIME2 based workflows. A similar situation is observed with QIIME2 assigning a major taxa to an undefined genus belonging to the *Enterobacteriales* order, while AmpliconTagger assigned it to the Escherichia-Shigella genus (Fig. 2b). Even though the two training sets used to classify OTUs/ASVs are built from the Silva 128 release, the QIIME2 training set was trained on a 99% identity clustered database and the AmpliconTagger training set was trained on a 100% identity (*i.e.* unclustered) fasta database, which probably explains the differences observed in taxonomy, which, all things considered, are quite minimal.

Robustness of ecological patterns between OTU and ASV based results.

One of the key advantages of the pipeline introduced here is that it is modular and can be customized to fit the research needs of the user. For instance, we have shown here that both ASV and OTU generation methods can be used interchangeably, making the Amplicon Tagger pipeline agnostic to the current heated debate about ASV vs. OTU. ASV generation is a method that has recently gained traction[33] and is increasingly being adopted as an alternative to OTU-based analyses. Here we processed seven types of datasets and compared end results of OTU clustering procedures done with DNACLUST, VSEARCH and two ASV based method (Deblur and DADA2). In most

cases were short reads are being analyzed, results given by Deblur and DADA2 are essentially identical to what is obtained by VSEARCH and DNACLUST. One notable exception is for the ITS data type where the weighted UniFrac Mantel correlations between the four methods are significantly lower than what is observed for short 16S and 18S amplicon data types. This discrepancy might be due to the fact that ITS amplicon sequence lengths are quite variable as shown in figure S8 (Additional file 1), with significant amounts of reads dispersed from 200 to 350 bp. During the clustering process, many of the shorter reads are “absorbed” by the larger reads. For ASVs, all these reads of various lengths eventually form a distinct ASV and in consequence many ASVs do not reach the cutoff of at least 25 reads per ASV. This holds true for all projects, were the number of ASV reads is often lower than the number of OTU reads (table 2), but is probably exacerbated in the ITS data were much of the amplicon sequences are of different lengths (Additional file 1 - Fig. S8). That said, one would still have expected the weighted UniFrac mantel correlation to be higher between DNACLUST and VSEARCH OTUs - as observed in other data types - which was not the case. On the other hand, Bray-Curtis dissimilarity matrices were highly concordant between Deblur, DADA2, VSEARCH and DNACLUST for the ITS data type. This implies that global alignments, inherent to generating the Unifrac distance matrix, may be impractical because of the high variability in sequence and length of ITS amplicons, as previously suggested [34].

PacBio long reads

PacBio taxonomic profiles of the mock community generated by AmpliconTagger were consistent with what was expected (Fig. S6a). While taxonomic profiles obtained with ASVs for the PacBio mock community are generally consistent with what is obtained with OTUs (Fig. S6a), there seems to be a low but consistent proportion of OTUs that are assigned closely related species in addition to the expected targets. For instance, the mock community contained two species belonging to the *Staphylococcus* genus (Fig. S6a): *Staphylococcus aureus* and *Staphylococcus epidermidis*. These two species have been identified in the OTU and ASV data, but the OTUs were assigned more closely related species such as *Staphylococcus saccharolyticus*, an uncultured *Staphylococcus* and undefined *Staphylococcus* species (*i.e.* Other). Regardless of the nature of the data (ASVs or OTUs), taxonomic assignment of PacBio CCS were accurate for the majority of the species of the mock community (Fig. S6a - *Actinomyces*, *Bacteroides*, *Clostridium*, *Deinococcus*, *Enterococcus*, *Helicobacter*, *Listeria*, *Neisseria*, *Propionibacterium*, *Pseudomonas* and *Streptococcus* panels), but seemed to be more challenging for *Escherichia coli*, *Bacillus cereus* and *Staphylococcus epidermidis*. For instance, we expected to identify the *Bacillus cereus* species, but instead observed the *Bacillus Antharaxis* (VSEARCH, Deblur and DADA2) and an undefined bacillus (Other) (DNACLUST).

The taxonomic profiles of the more complex oral microbiome samples, were overall highly similar to up to the species level with the majority of ASVs/OTUs pointing toward *Streptococcus pseudopneumoniae* and *Streptococcus salivarius* related species. Some differences were also observed in less abundant species: Deblur was encirched in

Streptococcus vestibularis while DADA2 contained *Streptococcus mitis* and *Streptococcus sanguinis*.

Our results highlight the challenge of properly assigning species taxa to long 16S reads using general databases, in our case the Silva DB. This suggests that in some instances, PacBio CCS reads contain at least enough errors to cause misclassification at the species level when processed as ASVs and that clustering reads (OTU methods) in the objective of correcting these errors also result in misclassification.
Deblur ASVs in the mock community gave poor Mantel *r* correlation with other methods because although it held a total of 6,535 reads, 4 samples did not meet the cutoff of 1,000 reads required in the rarefaction procedure to normalize ASV tables, hence resulting in the rejection of 4 samples and lowering Mantel *r* correlations involving Deblur. To the best of our knowledge, Deblur was not optimized for the processing of long reads, but we wished to investigate if, along with DADA2, the ASV paradigm could be applied to this type of data. In that regard the recent implementation of DADA2 geared for PacBio CCS reads [35], effectively managed to integrate more reads into ASVs in comparison with Deblur. However, Mantel correlations of both ASV methods (Deblur and DADA2) with OTUs (VSEARCH and DNACLUST) were fairly low for long 16S reads for the mock community. For the oral microbiome samples, OTU-based (VSEARCH and DNACLUST) communities were highly similar to DADA2's ASVs but different from Deblur's ASVs (Table 3; weighted UniFrac). However, when comparing Bray-Curtis dissimilarity indexes, not factoring in phylogeny, microbial communities generated with OTUs were arguably different from the ones obtained by ASV methods

(Table 3).

Compared to short reads, long PacBio amplicon data showed lower Mantel correlations between the four tested methods (Table 3). PacBio Circular Consensus Sequence (CCS) reads have an accuracy of 99.999% according to the manufacturer's specifications. However, with long amplicons of 1,400 bp, even such a high percentage of accuracy will eventually translate into actual errors, inevitably reflecting a high proportion of low abundant sequences in OTU/ASV tables. This problematics is partially compensated for with clustering methods as reads having at least 97% identity will be merged in the same OTU. With the ASV paradigm, reads showing differences in a single base will form distinct ASVs resulting in a more scattered abundance table, which inevitably affects indexes based on abundance such as the Bray-Curtis dissimilarity index.

Overall, the choice of distance/dissimilarity metric (weighted UniFrac or Bray-Curtis) had a remarkable impact on our most "distinctive" datasets: 1) highly diverse amplicon lengths for the ITS datasets, 2) long sequences for the PacBio data and 3) simple bacterial population for the mock community data.

OTUs and ASVs

OTU generation methods can be divided into two broad categories referred to as close-reference and denovo methods. Comparison between the two paradigms have been the

subject of recent debates[36,37] and we have focused our attention here on *denovo* methods for OTU generation (DNACLUST and VSEARCH). More recently, the adoption of ASVs as an alternative to OTUs has gained traction in the microbial ecology community, mainly to avoid the arbitrary dissimilarity clustering threshold inherent to denovo OTU generation methods. One reported advantage of ASV is the higher resolution that they provide compared to OTUs - because no signal is lost during the sequence clustering process. ASVs have been reportedly used to distinguish between bacteria at the species level, but such a practice of identifying short amplicons at the species and strain level is controversial as there is no uniformly accepted definition of bacterial species[38,39] or strain[38]. Moreover, even with PacBio long amplicon rRNA data (1,400 bp reads) we often cannot confidently assign taxonomy at the species level using a defined mock community (personal observations and figure S6a). Therefore, inferring classification of short amplicons of a few hundred bases data to up to the species level should be considered with extreme caution. Besides, our data shows that, except for ITS and long PacBio (for the reasons explained above) 16S amplicons, ecological patterns (taxonomy, beta- and alpha- diversity) were very similar between ASVs and OTUs in the 16S and 18S based-studies we analyzed which is consistent with recent studies comparing ASVs and OTUs[40,41]. Another argument in favor of adopting ASVs is that they should allow to compare different studies without the need to recompute OTUs[33]. However, reusability across studies assumes that DNA of these different studies have been extracted using the same method[42,43] and DNA amplified using the same primer sequences. If sequencing libraries to be analyzed meet these criteria, in practice, it is probably more opportune to pool all libraries together and re-

initiate ASV generation to make sure that samples have been processed the same way (e.g. exact same parameters) in upstream steps of ASVs/OTUs generation. Given the constant improvement of compute hardware and efficiency of OTU clustering methods, re-generating OTUs or ASVs as new datasets for a given project gets available is certainly a viable option: our results (Fig. 1 - right panels) show that VSEARCH completed in about 4.5 hours compared to 5.2 hours for Deblur for the indoor microbiome project data, but that VSEARCH significantly outperformed Deblur for the other datasets. Another aspect that we observed from our ASV tables is the “staircase” pattern typically observed in lower abundant ASV, which is illustrated using the ASV table from the AAD project with six samples as an example (Additional file 1 and table S4). From this table, we see that because ASVs are discerned at a single base resolution, multiple ASVs pointing to the same taxonomic groups are generated. Probably, because the sequencing errors are “corrected” or “absorbed” by large clusters during the clustering process, this staircase pattern is absent in OTU tables. There are situations were ASVs can be useful to achieve correlation between an amplicon sequence and its associated genome. In such a situation, an alignment of 100% identity between an ASV and a reference sequenced genome may be necessary to make such a correlation, because of its inherent nature, an OTU representative sequence would probably rarely achieve a perfect alignment against its associated reference genome. Regardless of the ASVs or OTUs generation method used, the use of short amplicon sequencing should mainly aim at offering a broad snapshot of the microbial communities at stakes in a biological system. In all cases, the Amplicon Tagger pipeline can be customized and accommodate any OTU or ASV generation method, being

agnostic to the current debate.

In conclusion, microbial ecology is more than ever relying on high-throughput sequencing technologies. Bioinformatic pipelines used for analyzing these data loads are increasing in complexity and there is a need for increased flexibility in the systematic analysis of short rRNA amplicon data. End-to-end pipelines do exist, but these solutions are not necessarily conducive to an easy integration of third party packages or in-house software. Moreover, these pipelines, as they are provided, are mostly geared toward interactive or single batch job processing and can generate inessential intermediate files, which can be constraining in a production context. AmpliconTagger is intended to provide a backbone for automated short amplicon data processing with an easy way for literate Python coders for adding or removing jobs and steps and thus customize the pipeline to their specific needs or preferences.

Potential implications

High throughput nucleic acid sequencing is entering public life and is getting increasingly democratized. However, the bioinformatics analysis dimension that comes with nucleic acid sequencing projects is still often underestimated or poorly considered in the overall planning of sequencing data processing. Bioinformatic pipelines are complex with many different fine-tuned steps and there is a need for flexibility for parametrization and customization. The objective of the present study was to provide an example of a fully functional automated pipeline to process a variety of rRNA amplicon sequencing data types. Short amplicon data size is inherently small compared to other

high throughput sequencing fields such as shotgun metagenomics or large eukaryotes genome sequencing. This small data type was chosen specifically to illustrate the proof of concept of creating a highly customized marker gene pipeline offering bioinformaticians who operate them a suitable alternative to existing widespread solutions such as QIIME and Mothur. AmpliconTagger is integrated into the GenPipes workflow management system[44] and as such, it is easily customizable to adapt for specific needs and is practical for the integration of external bioinformatic packages. It allows leveraging compute job schedulers that are part of modern HPC environments and options tweaking and optimization. For instance, a clinical laboratory performing the monitoring of microbial communities could effectively add a step that compute source tracking[45] - to predict the source of microbial communities in a set of samples - for each pipeline run. A laboratory with research interests in non-conventional marker genes (e.g. *cpn60* and *rpoB*) or functional genes such as *phoD* and *pmoA* could also build their own reference database and training sets and promptly integrate them into the workflow.

Methods

Structure of the AmpliconTagger workflow

This is the main structure of the AmpliconTagger workflow. Specific parameters mentioned in this section reflect the ones that were used in this study, but can be customized as per the user’s needs as described in the user guide (Additional file 4).

1. Reads are first scanned for contaminants (*e.g.* Illumina, 454 or PacBio adapter

sequences) and PhiX reads using a Decontamination Using Kmers approach (bbduk, part of the bbmap software (unpublished - http://sourceforge.net/projects/bbmap/). Usually, a small proportion of reads are contaminants and accordingly, 0-25% are PhiX reads.

2. Removal of unpaired reads. From step 1, paired-end reads may be disrupted. This means that one of the read pairs might be lost due to the screening in step #1. All of these unpaired reads are discarded. This is usually a fairly small proportion of all reads. This step is not performed if reads are single ended (MiSeq single ended, PacBio, IonTorrent or 454).
3. If reads are of single-end configuration (*i.e.* 454, IonTorrent or PacBio data types), they are trimmed to a fixed length that is variable depending on the quality of sequencing run and amplicon length. If reads are paired end (Illumina), trimming can be optional and should be done in such a way that enough bases are left on the 3’ end of each read pair to allow assembly using forward (reads 1) and reverse (reads 2) common overlapping parts during merging of read pairs in the next step. Nevertheless, in the case of paired-end reads, it is arguably preferable to trim the 3’ portion of reads that shows low quality before reads performing the overlapping paired assembly.
4. if paired-end reads : Reads are assembled (overlapping paired assembly) using FLASH software (FLASH, RRID:SCR_005531)[46].
5. Primer sequences may or may not be removed from the assembled/single end reads. Primer sequences should be removed when possible/applicable as the primer annealing regions of amplified DNA may be overrepresented in

sequencing errors (personal observations). In the case of long PacBio reads, it has been observed that CCS reads can be generated in both forward and reverse orientation. In that case, reverse reads should be orientated in the correct orientation using primer sequence information.

6. The trimmed assembled/single-end reads from steps #4-5 are filtered for quality. All reads having an average quality score lower than between 27 to 33 or more than 0 or 1 N (undefined base) and 5 nucleotides below quality 15 are discarded. The remaining reads will be referred to as filtered reads from now on. Filtering parameters are customizable and should be adapted to the quality profile of each dataset.
7. Filtered reads are then clustered with our in-house clustering workflow. Briefly, reads are clustered at 100% identity and then clustered/denoised at 99% identity (VSEARCH, DNACLUST -[29,30]) (DNACLUST, RRID:SCR_001771). Clusters having abundances lower than 25 are discarded. Remaining clusters are then scanned for chimeras with VSEARCH's version of UCHIME denovo and UCHIME reference[29,47](UCHIME, RRID:SCR_008057) and clustered at 97% (DNACLUST or VSEARCH) to form the final clusters/OTUs. In the case of Deblur and DADA2, ASVs having abundance lower than 25 are discarded. For PacBio long amplicons, filtered reads are clustered at 97% identity and clusters having less than 2 reads (*i.e.* customizable parameter) are discarded. Remaining reads are scanned for chimera using UCHIME reference. If Deblur or DADA2 are used, filtered reads are used as input for Deblur or DADA2[31]. In the case of DADA2, input paired-end reads are provided as forward and reverse reads separately

(not paired-end assembled). Resulting Deblur ASVs are then scanned for chimeras with VSEARCH's version of UCHIME denovo and UCHIME reference[29,47]. DADA2's ASVs are filtered for chimera using the package's internal algorithm (removeBimeraDenovo) followed by Vsearch's UCHIME reference.

8. OTUs/ASVs are then assigned a taxonomic lineage with the RDP classifier[48] using an in-house training set containing the complete Silva release 128 database[49] supplemented with eukaryotic sequences from the Silva databases and a customized set of mitochondria, plastid and bacterial 16S sequences. ITS2 database consist of the UNITE ITS database (ITS1-ITS2) region. The 18S training set was built with the Silva eukaryote release 128 database. The RDP classifier gives a score (0 to 1) to each taxonomic depth of each OTU. Each taxonomic depth having a score >= 0.5 is kept to reconstruct the final lineage. OTU/ASV are also blasted against the most recent NCBI nt database for complementary information.

9. Using taxonomic lineages obtained from step 8 combined with cluster abundance from step 7, a raw OTU/ASV table is generated. From that raw OTU/ASV table, an OTU/ASV table containing both bacterial and archeal organisms is generated. From this latter OTU/ASV table, a normalized (edgeR -[50,51])(edgeR, RRID:SCR_012802) and consensus rarefied (as described in more details below) OTU/ASV table is generated. If data consists of ITS amplicons, the same procedures are applied, but the raw OTU/ASV table is filtered to keep fungal organisms only. If the data is derived from 18S amplicons, the OTU/ASV table is

filtered to keep eukaryotic organisms only. From this point on, the rarefied consensus OTU/ASV table is used for downstream analyses.

10. A summary of read counts throughout the different steps of the pipeline is generated. This is useful to get a global outlook on the sequencing run: how many reads were sequenced, how many reads were filtered out after QC, how many OTUs/ASVs were generated, etc.

11. From these classified OTUs/ASVs, a multiple sequence alignment is then obtained by aligning OTU/ASV sequences on a Greengenes core reference alignment[52] using the PyNAST aligner[17]. If data type is ITS or 18S, OTU/ASV sequences are aligned against a Unite or Silva eukaryote core alignment, respectively. For short amplicon data, Alignments are filtered to keep only the hypervariable region of the alignment. For long PacBio reads, the whole alignment is being kept.

12. A phylogenetic tree is then built from that alignment (from step 11) with FastTree (FastTree, RRID:SCR_015501)[53]. Alpha (observed species) and beta (-weighted, unweighted UniFrac and Bray Curtis distances) diversity metrics and taxonomic summaries are then computed using the QIIME 1 software suite (QIIME, RRID:SCR_008249)[17,54]. Along with the OTU/ASV tables, these last tables represent end results from the pipeline and can then be used to generate various types of plots and statistics computation.

Reads clustering and ASV methodology.

Our OTU generation procedure was implemented based on a procedure previously described (Lundberg et al., 2012)[55] and uses either DNACLUST of VSEARCH for the reads clustering step. Briefly, quality controlled reads/sequences are de-replicated at 100% identity. The dereplication step is necessary to lower data load for the clustering software as only one representative of many thousands of identical sequences are kept for clustering. Counts of each unique sequence representative are kept in sequence headers after the dereplication process. For instance for the AAD study, the fastq file holding quality controlled paired-end assembled reads holds 9,209,510 sequences. Once de-replicated, these sequences are actually regrouped into 831,570 sequences which represent a 11.1-fold data reduction. These dereplicated sequences are then clustered at 99% identity (DNACLUST of VSEARCH). Clusters having an abundance of less than 25 reads (*e.g.* customizable parameter) are then discarded and the remaining clusters are then scanned for chimeras with UCHIME denovo and UCHIME reference[47] and clustered again at 97% identity (DNACLUST or VSEARCH) to form the final clusters.

RDP classifier training sets.

The RDP classifier is a bayesian classifier whose purpose is to classify sequences against a training set. Existing training sets are based on 99% identity clustered versions of either Greengenes or Silva databases. The RDP database (not to be confused with the RDP classifier software) was also built in a similar manner. In order to improve resolution of classification, we built our own custom training sets using the whole Silva SSU (release 128) database. We had to semi-automatically and manually

alter the classification of certain taxa in order to make the lineages unique and non-conflicting. At the time of writing, we are using a training set based on the Silva 128 release. We also built our own training sets for 18S and ITS sequences. The taxonomic classification system for eukaryotic organisms is far more complex that what it is for the simpler bacterial kingdom. As such, additionally to the common kingdom, phylum, class, order, family and genus fields found in prokaryotic taxonomy, eukaryotic taxonomy includes ranks such as subphylum, subdivision, subclass, superorder, suborder and subfamily, which makes the task of generating consistent values for each rank challenging. Importantly, in order to obtain more resolution from taxonomic classifications, our training sets were generated using the whole databases of the Silva and Unite databases and not the clustered or "OTU" versions (*i.e.* clustered at various identity thresholds ranging from 95 to 99%) of these databases. Perl code used to generate training sets and training sets themselves are available in the following repository: https://github.com/jtremblay/RDP-training-sets.

Normalizing OTU/ASV tables with a multi-rarefaction procedure.

Normalization of ASVs or OTUs is a controversial topic[50,56]. Until a durable solution gets accepted by the microbial ecology research community, we favor a multi-rarefaction approach as a mean to generate a normalized OTU/ASV table. Briefly, the raw OTU table is first filtered for targeted microorganisms - if 16S primers were used, only OTUs/ASVs matching to Bacteria at the kingdom level will be kept for downstream steps. This filtered OTU/ASV table is then rarefied 500 times and the mean of each OTU/ASV of each sample is then computed so that a consensus rarefied table is

obtained. Proceeding this way avoids the bias introduced by performing a single random rarefaction, which inevitably leaves out low abundance microorganisms. This consensus rarefied table is then used for downstream analyses (alpha-, beta- diversity, taxonomic summaries, etc.).

Smart restart mechanism using a workflow management system.

Bioinformatics pipelines are intrinsically complex with many steps that need to be executed in a specific order. In order to improve productivity, pipelines should be executed on a compute cluster using a compute job scheduler (e.g. Torque, SLURM) supporting job dependencies. This way, the jobs of a complex pipeline can be submitted all at once to the job scheduler so that each job can be available for execution only when their depending job has successfully completed. For example, in a typical rRNA gene amplicon pipeline, the OTU/ASV generation job(s) can enter the waiting queue only when the quality control job it depends on have all been successfully completed. Only then, OTU/ASV generation job will enter the queue for execution. Many pipeline modules (software that generates scripts of job submissions) have been written and published[57]. A good pipeline framework should generate jobs, manage their dependencies and have a smart restart mechanism in case of job failure. In the context of a complex pipeline with hundreds to thousands of jobs, a smart restart mechanism is indispensable to gain productivity and save time determining which job failed. GenPipes fills these requirements and is the reason for its adoption [21]. For instance if the execution of AmpliconTagger gets interrupted because of a job failure, it should be straightforward to identify exactly which job failed to properly execute. With a smart

restart mechanism implementation, the pipeline framework should find, upon re-execution, which job actually failed to successfully complete and effectively rewrite them for re-submission. Bioinformatics pipeline frameworks are also critical in that they allow sequencing data to be systematically analyzed in reproducible ways and that each step or job that it generates is parameterizable. For instance when analyzing quality controlled read data results, one can realize that the quality filtering parameters were too stringent given the quality score profiles of the input sequencing data. By slightly decreasing the quality filtering parameters and re-running the pipeline framework, all the downstream jobs affected by this modified parameter will be re-generated and re-submitted to the job scheduler. Proceeding with a pipeline framework also leaves traces of parameters used in all jobs should the data and analyses be revisited in the future.

Sequencing library preparation for mock community DNA.

Mock communities purified DNA was purchased form BEI resources (Manassas, VA) as HM-782D (even spike-in of total mock community). 16S rRNA gene amplicon libraries were prepared as described[58].

Availability of source code and requirements

Project Name: AmpliconTagger

Project Home Page: http://jtremblay.github.io/amplicontagger.html

Operating System: CentOS 7

Programming Languages: Python, Perl, R

Other requirements: pynast/1.2.2; perl/5.26.0; rdp_classifier/2.5; fasttree/2.1.10;

FLASH/1.2.11; qiime/1.9.1; duk/1.051; DNACLUST/3; fastx/0.0.13.2; python/2.7.5; python/3.6.5; java/jdk1.8.0_144; blast/2.6.0+; Deblur/1.0.4; VSEARCH/2.7.1; R/3.6.0, DADA2/1.12.1

License: GNU GPL

Code availability.

The AmpliconTagger pipeline wrapper code and Python, Perl and R scripts that are being called by AmpliconTagger are available here:

https://bitbucket.org/jtremblay514/nrc_pipeline_public/src/1.1/

https://bitbucket.org/jtremblay514/nrc_tools_public/src/1.1/

External software packages module install scripts are available here:

https://bitbucket.org/jtremblay514/nrc_resources_public/src/1.1/

A Docker image built on the CentOS 7 operational system which contains all necessary modules for full pipeline functionality is available for testing/evaluation purposes and running small datasets (https://cloud.docker.com/u/julio514/repository/docker/julio514/centos). Scripts used to generate RDP training sets are available here: and the training sets files are available on the docker image. The PipelineViewer web page is located here: http://jtremblay.github.io/PipelineViewer/amplicontagger.html and its source code is available here: https://github.com/jtremblay/PipelineViewer.

Availability of supporting data and materials

Sequencing for the indoor microbiome project is available through the ENA portal under accession number ERP005806. 16S rRNA amplicon sequence data for the AAD study is available in the NCBI's SRA portal under accession number SRP120170. PacBio full length 16S rRNA amplicons for the oral microbiome project is available under SRR56217[29-69]. ITS amplicons from the plant root microbiome transplant study are available under PRJNA301462. 18S rRNA gene amplicon data from the Chicago Michigan lake study is available under PRJNA294919/SRP063479. The even mock community reads are available under PRJNA510326. The staggered mock community is available under SRR2082918-20 and the PacBio mock libraries under SRR559331[4]-[7], SRR55933[19]-[20] and SRR559333[2]-[3]. All raw data and intermediate files used and generated for this study are available in GigaDB[59]. All commands used to process all of the six datasets are also available in GigaDB [59].

Declarations

List of abbreviations

ASV = Amplicon Sequence Variant; OTU = Operational Taxonomic Unit; Gb = Giga-base; GB = Gigabyte; rRNA = ribosomal RNA; CCS = Circular Consensus Sequence; HPC = High Performance Computing; bp = base-pairs; GB = Gigabyte; MB = Megabyte.

Competing Interest

The authors declare that they have no competing interests.

Author contributions

JT planned the experimental design, wrote the software, analyzed the data and wrote the manuscript. EY edited the manuscript.

Acknowledgments

We wish to acknowledge Compute Canada for access to both the Waterloo University (Graham system) and McGill University (Guillimin system) High Performance Computing (HPC) infrastructures. We thank Charles W Greer for editing the manuscript and Jessica Wasserscheid for editing the user guide.

Figure legends

Figure 1. Comparison between Deblur, DADA2, DNACLUST and VSEARCH as implemented in AmpliconTagger and QIIME2-VSEARCH, QIIME2-DADA2 and QIIME2-Deblur for the taxonomic profiles of a) even and b) staggered mock community, c) beta diversity and d) alpha diversity of mock community samples (16S V4 region; 2x250 bp). Results labelled with the QIIME2- prefix were entirely processed with QIIME2 using either VSEARCH, Deblur or DADA2 as OUT or ASV generation method.

Figure 2. Resources consumption for investigated datasets and each OTUs/ASVs generation method. There are no common core steps for QIIME2-DADA2 workflow as raw reads were submitted to DADA2 directly.

References

1. Human Microbiome Project Consortium. A framework for human microbiome research. Nature. 2012;486:215–21.

2. Human Microbiome Project Consortium. Structure, function and diversity of the healthy human microbiome. Nature. 2012;486:207–14.

3. Integrative HMP (iHMP) Research Network Consortium. The Integrative Human Microbiome Project: dynamic analysis of microbiome-host omics profiles during periods of human health and disease. Cell Host Microbe. 2014;16:276–89.

4. Thompson LR, Sanders JG, McDonald D, Amir A, Ladau J, Locey KJ, et al. A communal catalogue reveals Earth's multiscale microbial diversity. Nature. 2017;551:457–63.

5. Watson-Haigh NS, Shang CA, Haimel M, Kostadima M, Loos R, Deshpande N, et al. Next-generation sequencing: a challenge to meet the increasing demand for training workshops in Australia. Brief Bioinform. 2013;14:563–74.

6. Daber R, Sukhadia S, Morrissette JJD. Understanding the limitations of next generation sequencing informatics, an approach to clinical pipeline validation using artificial data sets. Cancer Genet. 2013;206:441–8.

7. Li P-E, Lo C-C, Anderson JJ, Davenport KW, Bishop-Lilly KA, Xu Y, et al. Enabling the democratization of the genomics revolution with a fully integrated web-based bioinformatics platform. Nucleic Acids Res. 2017;45:67–80.

8. Shringarpure SS, Carroll A, De La Vega FM, Bustamante CD. Inexpensive and Highly Reproducible Cloud-Based Variant Calling of 2,535 Human Genomes. PLoS One. 2015;10:e0129277.

9. Afgan E, Baker D, van den Beek M, Blankenberg D, Bouvier D, Čech M, et al. The Galaxy platform for accessible, reproducible and collaborative biomedical analyses: 2016 update. Nucleic Acids Res. 2016;44:W3–10.

10. Alkema W, Boekhorst J, Wels M, van Hijum SAFT. Microbial bioinformatics for food safety and production. Brief Bioinform. 2016;17:283–92.

11. Hess M, Sczyrba A, Egan R, Kim T-W, Chokhawala H, Schroth G, et al. Metagenomic discovery of biomass-degrading genes and genomes from cow rumen. Science. 2011;331:463–7.

12. Manaka A, Tokue Y, Murakami M. Comparison of 16S ribosomal RNA gene sequence analysis and conventional culture in the environmental survey of a hospital. J Pharm Health Care Sci. 2017;3:8.

13. Martineau C, Li X, Lalancette C, Perreault T, Fournier E, Tremblay J, et al. Serratia marcescens Outbreak in a Neonatal Intensive Care Unit: New Insights from Next-Generation Sequencing Applications. J Clin Microbiol. 2018;56. doi: 10.1128/JCM.00235-18

14. Hewitt KM, Mannino FL, Gonzalez A, Chase JH, Caporaso JG, Knight R, et al. Bacterial diversity in two Neonatal Intensive Care Units (NICUs). PLoS One. 2013;8:e54703.

15. Bokulich NA, Mills DA, Underwood MA. Surface microbes in the neonatal intensive care unit: changes with routine cleaning and over time. J Clin Microbiol. 2013;51:2617–24.

16. Deurenberg RH, Bathoorn E, Chlebowicz MA, Couto N, Ferdous M, García-Cobos S, et al. Application of next generation sequencing in clinical microbiology and infection prevention. J Biotechnol. 2017;243:16–24.

17. Caporaso JG, Kuczynski J, Stombaugh J, Bittinger K, Bushman FD, Costello EK, et al. QIIME allows analysis of high-throughput community sequencing data. Nat Methods. 2010;7:335–6.

18. Schloss PD, Westcott SL, Ryabin T, Hall JR, Hartmann M, Hollister EB, et al. Introducing mothur: open-source, platform-independent, community-supported software for describing and comparing microbial communities. Appl Environ Microbiol. 2009;75:7537–41.

19. Callahan BJ, McMurdie PJ, Rosen MJ, Han AW, Johnson AJ, Holmes SP. DADA2: High resolution sample inference from amplicon data [Internet]. Available from: http://dx.doi.org/10.1101/024034

20. Bolyen E, Rideout JR, Dillon MR, Bokulich NA, Abnet C, Al-Ghalith GA, et al. QIIME 2: Reproducible, interactive, scalable, and extensible microbiome data science. PeerJ Preprints. 2018.

21. Bourgey M, Dali R, Eveleigh R, Chen KC, Letourneau L, Fillon J, et al. GenPipes: an open-source framework for distributed and scalable genomic analyses. GigaScience [Internet]. 2019;8. Available from:

https://doi.org/10.1093/gigascience/giz037

22. Tremblay J, Singh K, Fern A, Kirton ES, He S, Woyke T, et al. Primer and platform effects on 16S rRNA tag sequencing. Front Microbiol. 2015;6:771.

23. Lax S, Smith DP, Hampton-Marcell J, Owens SM, Handley KM, Scott NM, et al. Longitudinal analysis of microbial interaction between humans and the indoor environment. Science. 2014;345:1048–52.

24. Searle D, Sible E, Cooper A, Putonti C. 18S rDNA dataset profiling microeukaryotic populations within Chicago area nearshore waters. Data Brief. 2016;6:526–9.

25. MacPherson CW, Mathieu O, Tremblay J, Champagne J, Nantel A, Girard S-A, et al. Gut Bacterial Microbiota and its Resistome Rapidly Recover to Basal State Levels after Short-term Amoxicillin-Clavulanic Acid Treatment in Healthy Adults. Sci Rep. 2018;8:11192.

26. Yergeau E, Bell TH, Champagne J, Maynard C, Tardif S, Tremblay J, et al. Transplanting Soil Microbiomes Leads to Lasting Effects on Willow Growth, but not on the Rhizosphere Microbiome. Front Microbiol. 2015;6:1436.

27. Singer E, Bushnell B, Coleman-Derr D, Bowman B, Bowers RM, Levy A, et al. High-resolution phylogenetic microbial community profiling. ISME J. 2016;10:2020–32.

28. Wang Y, Zhang J, Chen X, Jiang W, Wang S, Xu L, et al. Profiling of Oral Microbiota in Early Childhood Caries Using Single-Molecule Real-Time Sequencing. Front Microbiol. 2017;8:2244.

29. Rognes T, Flouri T, Nichols B, Quince C, Mahé F. VSEARCH: a versatile open source tool for metagenomics. PeerJ. 2016;4:e2584.

30. Ghodsi M, Liu B, Pop M. DNACLUST: accurate and efficient clustering of phylogenetic marker genes. BMC Bioinformatics. 2011;12:271.

31. Amir A, McDonald D, Navas-Molina JA, Kopylova E, Morton JT, Zech Xu Z, et al. Deblur Rapidly Resolves Single-Nucleotide Community Sequence Patterns. mSystems [Internet]. 2017;2. Available from:

http://dx.doi.org/10.1128/mSystems.00191-16

32. Earl JP, Adappa ND, Krol J, Bhat AS, Balashov S, Ehrlich RL, et al. Species-level bacterial community profiling of the healthy sinonasal microbiome using Pacific Biosciences sequencing of full-length 16S rRNA genes. Microbiome. 2018;6:190.

33. Callahan BJ, McMurdie PJ, Holmes SP. Exact sequence variants should replace operational taxonomic units in marker-gene data analysis. ISME J. 2017;11:2639–43.

34. Lindahl BD, Nilsson RH, Tedersoo L, Abarenkov K, Carlsen T, Kjøller R, et al. Fungal community analysis by high-throughput sequencing of amplified markers--a user’s guide. New Phytol. 2013;199:288–99.

35. Callahan BJ, Wong J, Heiner C, Oh S, Theriot CM, Gulati AS, et al. High-throughput amplicon sequencing of the full-length 16S rRNA gene with single-nucleotide resolution [Internet]. Available from: http://dx.doi.org/10.1101/392332

36. Westcott SL, Schloss PD. De novo clustering methods outperform reference-based methods for assigning 16S rRNA gene sequences to operational taxonomic units. PeerJ. 2015;3:e1487.

37. He Y, Caporaso JG, Jiang X-T, Sheng H-F, Huse SM, Rideout JR, et al. Stability of operational taxonomic units: an important but neglected property for analyzing microbial diversity. Microbiome. 2015;3:20.

38. Segata N. On the Road to Strain-Resolved Comparative Metagenomics. mSystems [Internet]. 2018;3. Available from: http://dx.doi.org/10.1128/mSystems.00190-17

39. Riley MA, Lizotte-Waniewski M. Population Genomics and the Bacterial Species Concept. Methods in Molecular Biology. 2009. p. 367–77.

40. Glassman SI, Martiny JBH. Broadscale Ecological Patterns Are Robust to Use of Exact Sequence Variants versus Operational Taxonomic Units. mSphere [Internet]. 2018;3. Available from: http://dx.doi.org/10.1128/mSphere.00148-18

41. Nearing JT, Douglas GM, Comeau AM, Langille MGI. Denoising the

Denoisers: an independent evaluation of microbiome sequence error-correction approaches. PeerJ. 2018;6:e5364.

42. Filippidou S, Junier T, Wunderlin T, Lo C-C, Li P-E, Chain PS, et al. Under-detection of endospore-forming Firmicutes in metagenomic data. Comput Struct Biotechnol J. 2015;13:299–306.

43. Wesolowska-Andersen A, Bahl MI, Carvalho V, Kristiansen K, Sicheritz-Pontén T, Gupta R, et al. Choice of bacterial DNA extraction method from fecal material influences community structure as evaluated by metagenomic analysis. Microbiome. 2014;2:19.

44. Bourgey M, Dali R, Eveleigh R, Chen KC, Letourneau L, Fillon J, Michaud M, Caron M, Sandoval J, Lefebvre F, Leveque G, Mercier E, Bujold D, Marquis P, Van PT, Anderson de Lima Morais D, Tremblay J, Shao X, Henrion E, Gonzalez E, Quirion PO, Caron B, Bourque G. GenPipes: an open-source framework for distributed and scalable genomic analyses. Gigascience. 2019 Jun 1;8(6). pii: giz037. doi:10.1093/gigascience/giz037.

45. Knights D, Kuczynski J, Charlson ES, Zaneveld J, Mozer MC, Collman RG, et al. Bayesian community-wide culture-independent microbial source tracking. Nat Methods. 2011;8:761–3.

46. Magoč T, Salzberg SL. FLASH: fast length adjustment of short reads to improve genome assemblies. Bioinformatics. 2011;27:2957–63.

47. Edgar RC, Haas BJ, Clemente JC, Quince C, Knight R. UCHIME improves sensitivity and speed of chimera detection. Bioinformatics. 2011;27:2194–200.

48. Wang Q, Garrity GM, Tiedje JM, Cole JR. Naive Bayesian classifier for rapid assignment of rRNA sequences into the new bacterial taxonomy. Appl Environ Microbiol. 2007;73:5261–7.

49. Quast C, Pruesse E, Yilmaz P, Gerken J, Schweer T, Yarza P, et al. The SILVA ribosomal RNA gene database project: improved data processing and web-based tools. Nucleic Acids Res. 2013;41:D590–6.

50. McMurdie PJ, Holmes S. Waste Not, Want Not: Why Rarefying Microbiome Data Is Inadmissible. PLoS Comput Biol. 2014;10:e1003531.

51. Robinson MD, McCarthy DJ, Smyth GK. edgeR: a Bioconductor package for differential expression analysis of digital gene expression data. Bioinformatics. 2010;26:139–40.

52. DeSantis TZ, Hugenholtz P, Larsen N, Rojas M, Brodie EL, Keller K, et al. Greengenes, a chimera-checked 16S rRNA gene database and workbench compatible with ARB. Appl Environ Microbiol. 2006;72:5069–72.

53. Price MN, Dehal PS, Arkin AP. FastTree 2--approximately maximum-likelihood trees for large alignments. PLoS One. 2010;5:e9490.

54. Kuczynski J, Stombaugh J, Walters WA, González A, Caporaso JG, Knight R. Using QIIME to analyze 16S rRNA gene sequences from microbial communities. Curr Protoc Bioinformatics. 2011;Chapter 10:Unit 10.7.

55. Lundberg DS, Lebeis SL, Paredes SH, Yourstone S, Gehring J, Malfatti S, et al. Defining the core Arabidopsis thaliana root microbiome. Nature. 2012;488:86–90.

56. Weiss S, Xu ZZ, Peddada S, Amir A, Bittinger K, Gonzalez A, et al. Normalization and microbial differential abundance strategies depend upon data characteristics. Microbiome. 2017;5:27.

57. Leipzig J. A review of bioinformatic pipeline frameworks. Brief Bioinform. 2017;18:530–6.

58. Yergeau E, Michel C, Tremblay J, Niemi A, King TL, Wyglinski J, et al. Metagenomic survey of the taxonomic and functional microbial communities of seawater and sea ice from the Canadian Arctic. Sci Rep. 2017;7:42242.

59. Tremblay J; Yergeau E (2019): Supporting data for "Systematic processing of rRNA gene amplicon sequencing data" GigaScience Database. http://dx.doi.org/10.5524/100658

 Click here to access/download;Table;tables.docx

Table1. Details of investigated datasets.

Study	Targeted gene and region	Average reads length of paired assembled fragments[1] (mean +/- standard deviation)	Sequencing configuration	Number of reads	Number of basepairs	Number of samples	File size of sequencing data (gzip compressed)
Even mock community (this study)	16S bacteria/archaea; V4	250.3 +/- 0.7 bp	Illumina 2x250 bp	1,987,408	0.50 Gb	4	375 MB
Staggered mock community[22]	16S bacteria/archaea; V4	250.2 +/- 0.7 bp	Illumina 2x250 bp	289,434	0.072 Gb	3	30 MB
Indoor microbiome[23]	16S bacteria; V3-V4 region	No assembled fragments, single end reads of 151 bp	Illumina 1x150 bp	111,093,697	16.8 Gb	1625	6.9 GB
Chicago nearshore water profiling[24]	18S eukaryotes; 1181F-1624R	250.5 +/- 3.1 bp	Illumina 2x150 bp	19,359,618	4.86 Gb	89	2.3 GB
Antibiotic-Associated Diarrhea[25]	16S bacteria/archaea; V4	250.4 +/- 0.8 bp	Illumina 2x250 bp	22,003,478	3.3 Gb	276	2.9 GB
Soil microbiome transplant[26]	Fungi ITS; ITS1	249.0 +/- 7.9 bp	Illumina 2x250 bp	30,775,636	7.72 Gb	94	4.1 GB
PacBio mock community[27]	16S bacterial; Full length	No assembled fragments, single end reads of 1472.7 +/- 215.5 bp	PacBio Single end sequencing	86,353	0.13 Gb	8	25 MB
Oral Microbiota[28]	16S bacterial; Full length	No assembled fragments, single end reads of 1470.2 +/- 225.8 bp	PacBio Single end sequencing	689,430	1.01 Gb	40	140 MB

[1]:These are the reads that are sent for OTUs/ASVs generation after having been paired-end assembled (for paired-end sequencing) and controlled for quality as described in methods.
bp = base-pairs, GB = Gigabyte, MB = Megabyte.

Table 2. Number of reads and OTUs/ASVs throughout AmpliconTagger's execution.

Project	OUT/ASV generation method	Total reads	Contaminants reads	Phix reads	Non contaminant and non phix reads	Non contaminant and non phix reads 1	Non contaminant and non phix reads 2	Reads 1 QC passed	Assembled reads	Assembled reads QC passed	Clustered or dereplicated sequences	Number of clusters or dereplicated sequences
**Mock community (V4 16S; paired-end)**	**Deblur**	2,602,808	8	27	2,276,776	1,138,388	1,138,388	-	1,123,408	1,032,461	597,547	67
	**DNACLUST**										918,257	67
	**VSEARCH**										973,750	34
	**DADA2**							928,310 R1 + 928,310 R2	-	-	885,235	96
	**QIIME2-Deblur**		-	-	-	-	-	-	966,899	966,829	599,522	46
	**QIIME2-VSEARCH**		-	-	-	-	-	-	966,899	966,829	916,980	37
	**QIIME2-DADA2**		-	-	-	-	-	-	-	-	980,184	124
**Indoor Microbiome (V4 16S; single end)**	**Deblur**	111,093	48,996	-	111,044,701	-	-	108,008,427	-	-	73,010,229	27,391
	**DNACLUST**										95,120,374	18,340
	**VSEARCH**										100,289,243	14,341
	**DADA2**										103,031,599	32,647
**Lake Michigan (1181F-1624R 18S; paired-end)**	**Deblur**	19,359,618	275,694	4,521	18,803,930	9,401,965	9,401,965	-	8,052,948	3,356,475	2,201,736	662
	**DNACLUST**										2,629,227	564
	**VSEARCH**										2,672,395	483
	**DADA2**							2,522,201 R1 +	-	-	1,880,759	854

								2,522,201 R2				
**AAD (V4 16S; paired-end)**	**Deblur**	22,003,478	151	809	22,001,808	11,000,904	11,000,904	-	10,860,416	9,209,510	5,657,445	1,560
	**DNACLUST**										7,791,719	1,053
	**VSEARCH**										8,100,048	827
	**DADA2**							7,435,547 R1 + 7,435,547 R2	-	-	6,583,440	1,791
**Plant microbiome transplant (ITS1 ITS; paired-end)**	**Deblur**	30,775,636	174,471	5,770,656	24,816,770	12,408,385	12,408,385		9,850,519	7,479,355	1,901,625	780
	**DNACLUST**										6,124,824	1,172
	**VSEARCH**										7,166,333	1,056
	**DADA2**							3,215,102 R1 + 3,215,102 R2	-	-	2,954,161	1,130
**Mock community (full length 16S; single end)**	**Deblur**	93,905	-	-	93,905	86,353	-	74,485	-	-	6,543	47
	**DNACLUST**										59,348	1,026
	**VSEARCH**										60,499	415
	**DADA2**										50,876	49
**Oral microbiome (full length 16S; single end)**	**Deblur**	627,138	-	-	627,138	562,986	-	562,896	-	-	219,423	199,780
	**DNACLUST**										520,992	77,305
	**VSEARCH**										523,444	69,478
	**DADA2**										262,414	6,535

* Setting Deblur`--min-reads` to 2 yielded 12 ASVs only and was therefore not investigated further. `--min-reads` was set to 25 for short amplicon data.
** Only some of the read counts statistics were available for deblur and non for dada2.

Table 3. Mantel *r* statistics comparing distance matrices of each ASVs/OTUs generation method for each project. Each *r* statistic had a *p* value < 0.001.

Weighted UniFrac

	Deblur vs DNACLUST	Deblur vs VSEARCH	Deblur vs DADA2	DNACLUST vs VSEARCH	DNACLUST vs DADA2	VSEARCH vs DADA2
Mock community (V4 16S; paired-end)	0.984	0.999	0.999	0.983	0.986	0.999
Indoor Microbiome (V4 16S; single end)	0.940	0.900	0.905	0.934	0.955	0.956
Lake Michigan (1181F-1624R 18S; paired-end)	0.952	0.956	0.949	0.986	0.977	0.978
AAD (V4 16S; paired-end)	0.943	0.943	0.942	0.968	0.970	0.958
Plant microbiome transplant (ITS1 ITS; paired-end)	0.403	0.508	0.542	0.403	0.465	0.617
Mock community (full length 16S; single end)	0.359	0.225	0.461	0.988	-0.078	-0.080
Oral microbiome (full length 16S; single end)	-0.097	-0.100	-0.093	0.987	0.958	0.953

Bray-Curtis

	Deblur vs DNACLUST	Deblur vs VSEARCH	Deblur vs DADA2	DNACLUST vs VSEARCH	DNACLUST vs DADA2	VSEARCH vs DADA2
Mock community (V4 16S; paired-end)	0.864	0.580	0.999	0.724	0.865	0.580
Indoor Microbiome (V4 16S; single end)	0.991	0.967	0.996	0.980	0.993	0.972
Lake Michigan (1181F-1624R 18S; paired-end)	0.994	0.996	0.993	0.997	0.993	0.991
AAD (V4 16S; paired-end)	0.960	0.941	0.993	0.980	0.967	0.949
Plant microbiome transplant (ITS1 ITS; paired-end)	0.821	0.812	0.792	0.979	0.935	0.914
Mock community (full length 16S; single end)	-0.331	-0.241	-0.119	0.901	0.023	0.194
Oral microbiome (full length 16S; single end)	0.046	0.040	-0.026	0.933	0.415	0.436

Figure 1

Click here to access/download;Figure;Figure 1.pdf

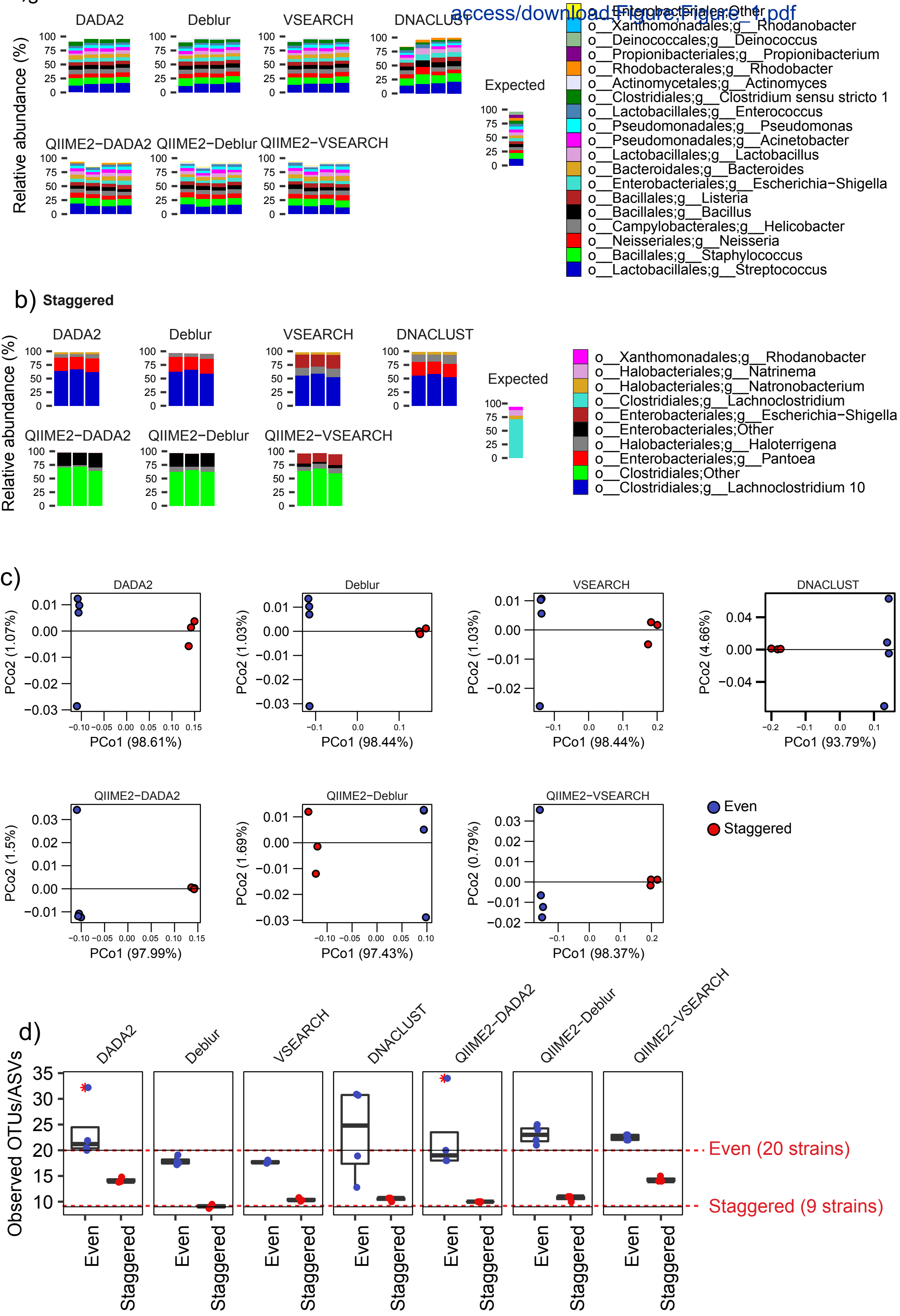

Figure 2

Click here to access/download;Figure;Figure_2.pdf

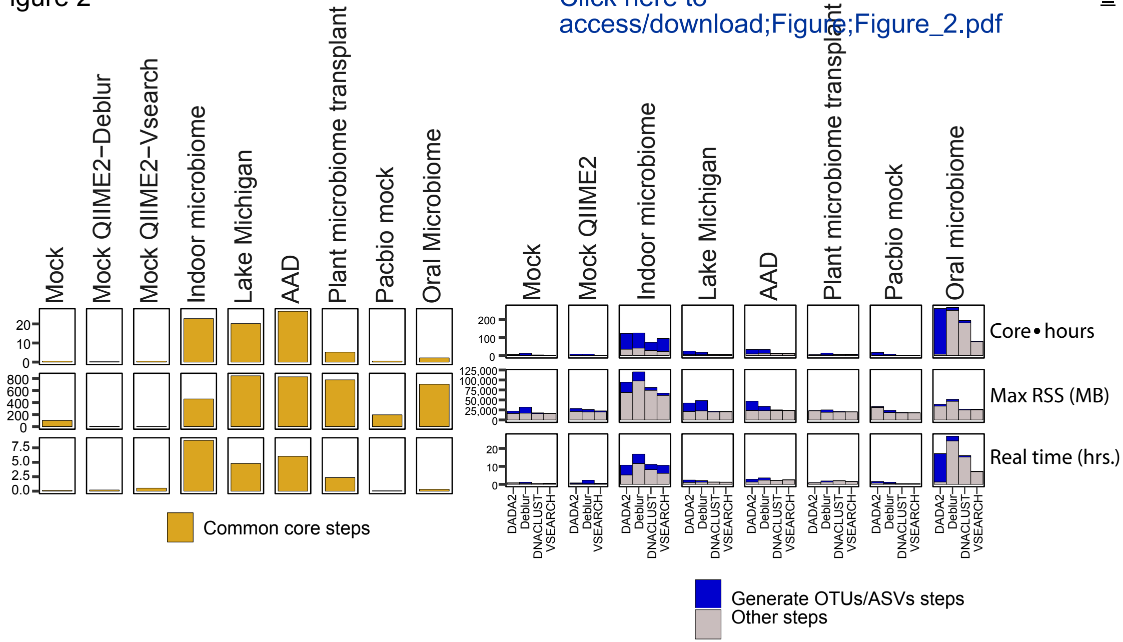

Additional file 1

Click here to access/download
Supplementary Material
Additional file 1.pdf

Additional file 2

Click here to access/download
Supplementary Material
Additional file 2.7z

Additional file 3

Click here to access/download
Supplementary Material
additional_file_3.docx

Additional file 4

Click here to access/download
Supplementary Material
Additional file 4.html

Supplementary tables S1,S2,S3,S4

Click here to access/download
Supplementary Material
supplementary_tables.xlsx
