# Supplementary material for: Systematic processing of ribosomal RNA gene amplicon sequencing data
Source: Gigascience. 2019 Dec 9;8(12):giz146. doi: 10.1093/gigascience/giz146 (PMC6901069; doi:10.1093/gigascience/giz146)
Supplement: giz146_Supplemental_Files [file giz146_supplemental_files.zip › Additional file 1.pdf]

Figure S1

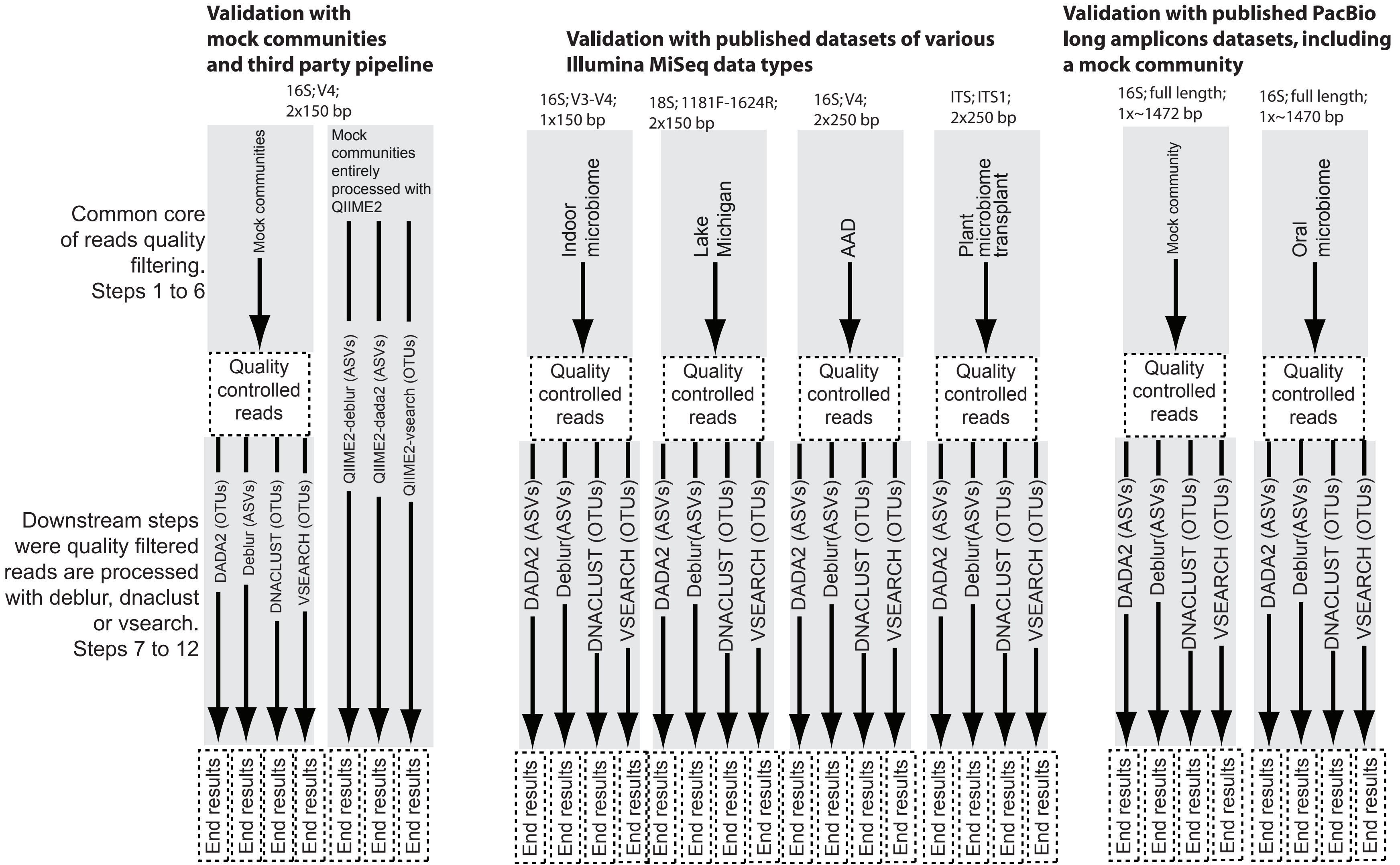
Validation with mock communities and third party pipeline
Validation with published datasets of various Illumina MiSeq data types
Validation with published PacBio long amplicons datasets, including a mock community
Common core of reads quality filtering. Steps 1 to 6
Downstream steps were quality filtered reads are processed with deblur, dnaclust or vsearch. Steps 7 to 12
16S; V4; 2x150 bp
Mock communities
Mock communities entirely processed with QIIME2
QIIME2-deblur (ASVs)
QIIME2-dada2 (ASVs)
QIIME2-vsearch (OTUs)
16S; V3-V4; 1x150 bp
Indoor microbiome
18S; 1181F-1624R; 2x150 bp
Lake Michigan
16S; V4; 2x250 bp
AAD
ITS; ITS1; 2x250 bp
Plant microbiome transplant
16S; full length; 1x~1472 bp
Mock community
16S; full length; 1x~1470 bp
Oral microbiome
Quality controlled reads
DADA2 (OTUs)
Deblur (ASVs)
DNACLUST (OTUs)
VSEARCH (OTUs)
DADA2 (ASVs)
Deblur(ASVs)
End results

Figure S2

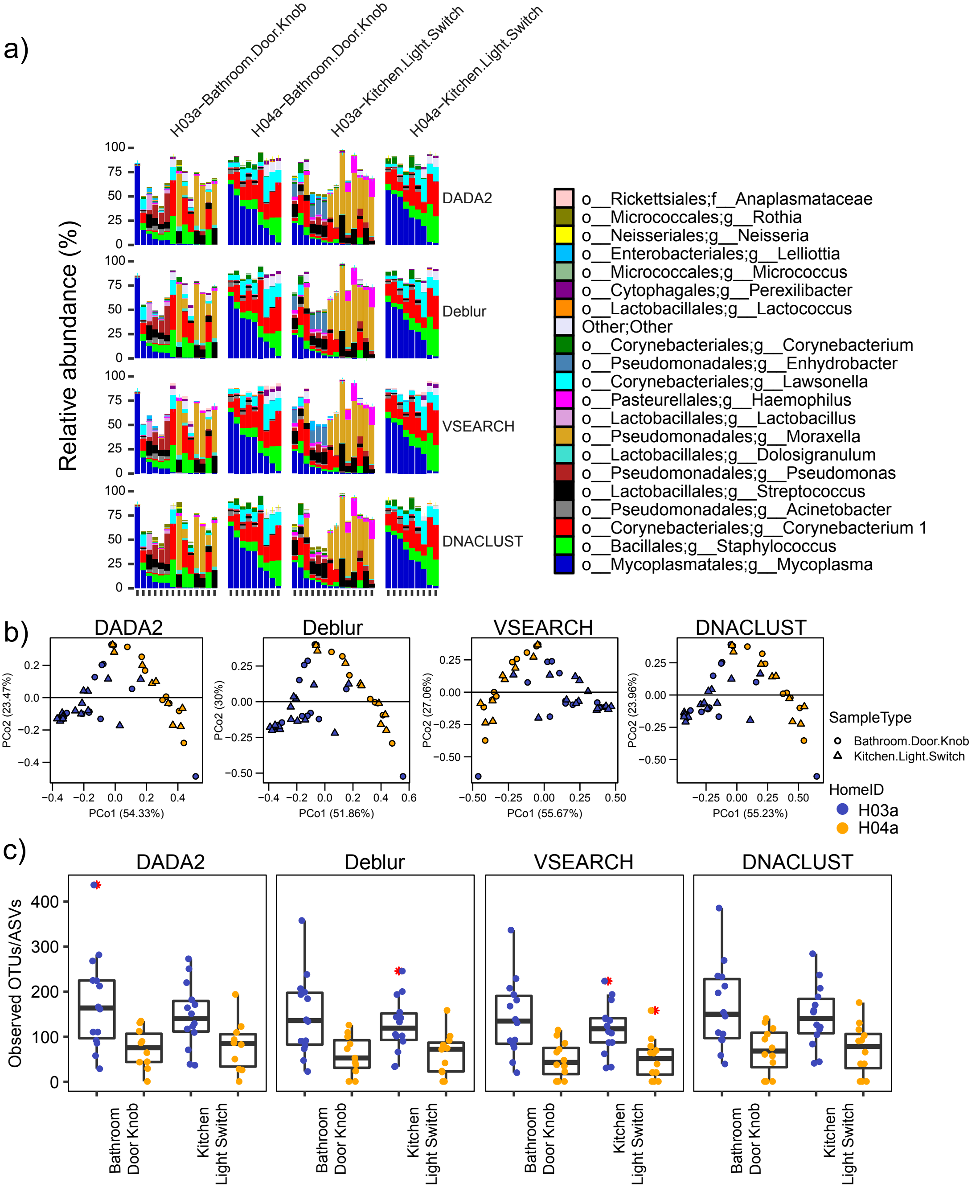

a)
H03a–Bathroom.Door.Knob
H04a–Bathroom.Door.Knob
H03a–Kitchen.Light.Switch
H04a–Kitchen.Light.Switch
Relative abundance (%)
DADA2
Deblur
VSEARCH
DNACLUST
o__Rickettsiales;f__Anaplasmataceae
o__Micrococcales;g__Rothia
o__Neisseriales;g__Neisseria
o__Enterobacteriales;g__Lelliottia
o__Micrococcales;g__Micrococcus
o__Cytophagales;g__Perexilibacter
o__Lactobacillales;g__Lactococcus
Other;Other
o__Corynebacteriales;g__Corynebacterium
o__Pseudomonadales;g__Enhydrobacter
o__Corynebacteriales;g__Lawsonella
o__Pasteurellales;g__Haemophilus
o__Lactobacillales;g__Lactobacillus
o__Pseudomonadales;g__Moraxella
o__Lactobacillales;g__Dolosigranulum
o__Pseudomonadales;g__Pseudomonas
o__Lactobacillales;g__Streptococcus
o__Pseudomonadales;g__Acinetobacter
o__Corynebacteriales;g__Corynebacterium 1
o__Bacillales;g__Staphylococcus
o__Mycoplasmatales;g__Mycoplasma
b)
DADA2
PCo1 (54.33%)
PCo2 (23.47%)
Deblur
PCo1 (51.86%)
PCo2 (30%)
VSEARCH
PCo1 (55.67%)
PCo2 (27.06%)
DNACLUST
PCo1 (55.23%)
PCo2 (23.96%)
SampleType
Bathroom.Door.Knob
Kitchen.Light.Switch
HomeID
H03a
H04a
c)
DADA2
Deblur
VSEARCH
DNACLUST
Observed OTUs/ASVs
Bathroom Door Knob
Kitchen Light Switch

Figure S3

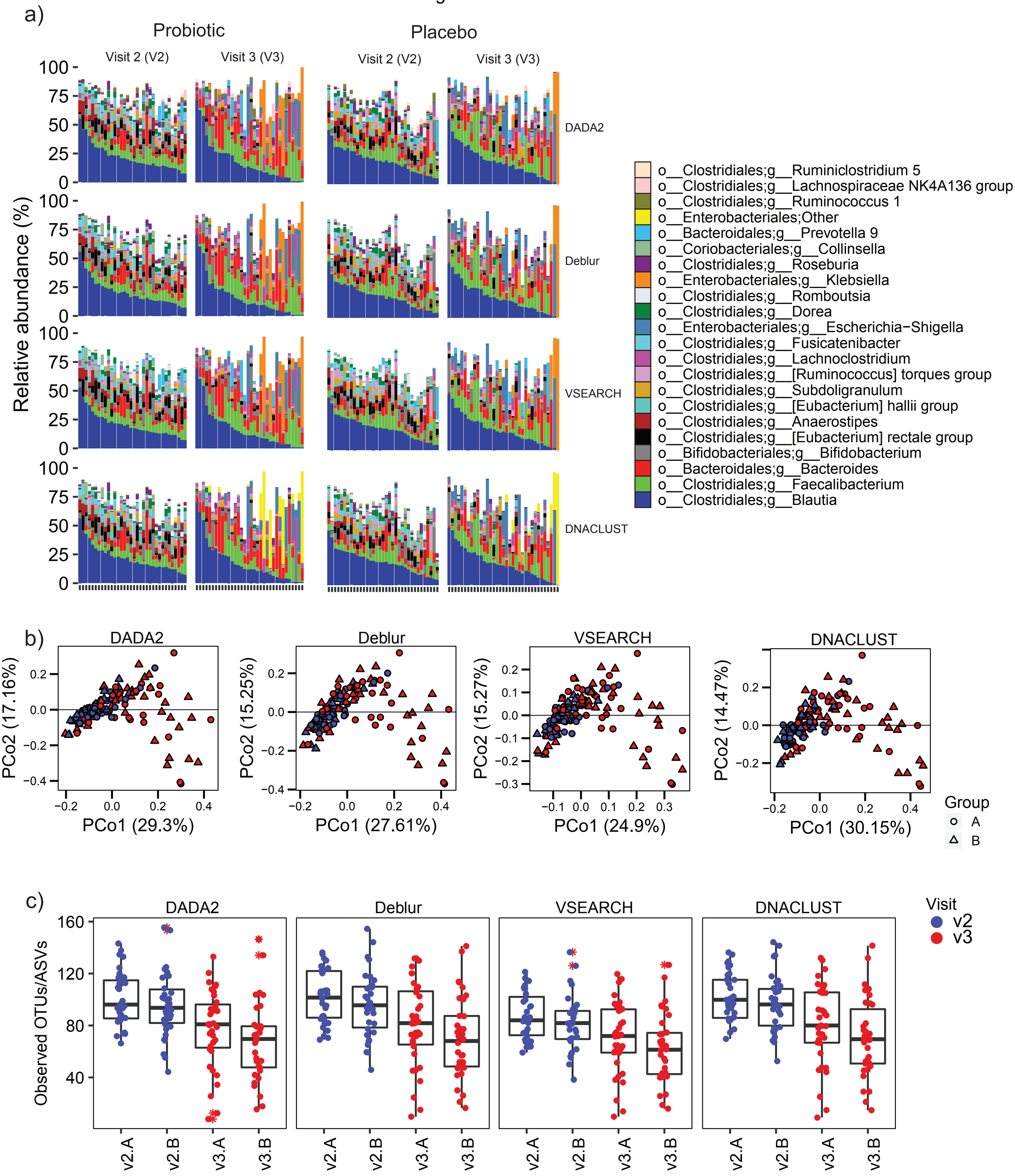
a)
Probiotic
Placebo
Visit 2 (V2)
Visit 3 (V3)
Visit 2 (V2)
Visit 3 (V3)
Relative abundance (%)
DADA2
Deblur
VSEARCH
DNACLUST
o__Clostridiales;g__Ruminiclostridium 5
o__Clostridiales;g__Lachnospiraceae NK4A136 group
o__Clostridiales;g__Ruminococcus 1
o__Enterobacteriales;Other
o__Bacteroidales;g__Prevotella 9
o__Coriobacteriales;g__Collinsella
o__Clostridiales;g__Roseburia
o__Enterobacteriales;g__Klebsiella
o__Clostridiales;g__Romboutsia
o__Clostridiales;g__Dorea
o__Enterobacteriales;g__Escherichia−Shigella
o__Clostridiales;g__Fusicatenibacter
o__Clostridiales;g__Lachnoclostridium
o__Clostridiales;g__[Ruminococcus] torques group
o__Clostridiales;g__Subdoligranulum
o__Clostridiales;g__[Eubacterium] hallii group
o__Clostridiales;g__Anaerostipes
o__Clostridiales;g__[Eubacterium] rectale group
o__Bifidobacteriales;g__Bifidobacterium
o__Bacteroidales;g__Bacteroides
o__Clostridiales;g__Faecalibacterium
o__Clostridiales;g__Blautia
b)
DADA2
Deblur
VSEARCH
DNACLUST
PCo2 (17.16%)
PCo1 (29.3%)
PCo2 (15.25%)
PCo1 (27.61%)
PCo2 (15.27%)
PCo1 (24.9%)
PCo2 (14.47%)
PCo1 (30.15%)
Group
A
B
c)
DADA2
Deblur
VSEARCH
DNACLUST
Observed OTUs/ASVs
Visit
v2
v3
v2.A
v2.B
v3.A
v3.B

Figure S4

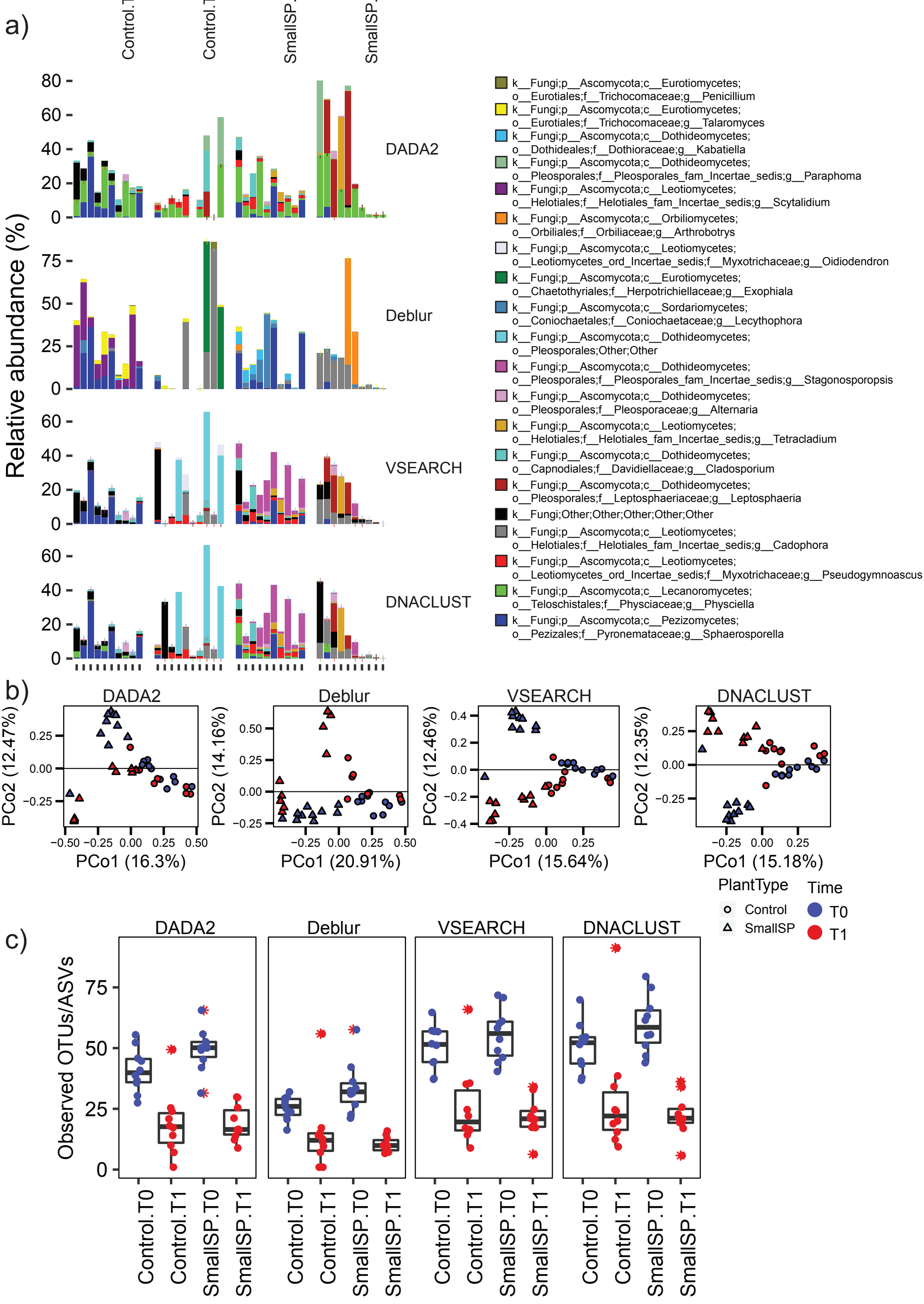

a)
Control.T0
Control.T1
SmallSP.T0
SmallSP.T1
Relative abundance (%)
DADA2
Deblur
VSEARCH
DNACLUST
k__Fungi;p__Ascomycota;c__Eurotiomycetes; o__Eurotiales;f__Trichocomaceae;g__Penicillium
k__Fungi;p__Ascomycota;c__Eurotiomycetes; o__Eurotiales;f__Trichocomaceae;g__Talaromyces
k__Fungi;p__Ascomycota;c__Dothideomycetes; o__Dothideales;f__Dothioraceae;g__Kabatiella
k__Fungi;p__Ascomycota;c__Dothideomycetes; o__Pleosporales;f__Pleosporales_fam_Incertae_sedis;g__Paraphoma
k__Fungi;p__Ascomycota;c__Leotiomycetes; o__Helotiales;f__Helotiales_fam_Incertae_sedis;g__Scytalidium
k__Fungi;p__Ascomycota;c__Orbiliomycetes; o__Orbiliales;f__Orbiliaceae;g__Arthrobotrys
k__Fungi;p__Ascomycota;c__Leotiomycetes; o__Leotiomycetes_ord_Incertae_sedis;f__Myxotrichaceae;g__Oidiodendron
k__Fungi;p__Ascomycota;c__Eurotiomycetes; o__Chaetothyriales;f__Herpotrichiellaceae;g__Exophiala
k__Fungi;p__Ascomycota;c__Sordariomycetes; o__Coniochaetales;f__Coniochaetaceae;g__Lecythophora
k__Fungi;p__Ascomycota;c__Dothideomycetes; o__Pleosporales;Other;Other
k__Fungi;p__Ascomycota;c__Dothideomycetes; o__Pleosporales;f__Pleosporales_fam_Incertae_sedis;g__Stagonosporopsis
k__Fungi;p__Ascomycota;c__Dothideomycetes; o__Pleosporales;f__Pleosporaceae;g__Alternaria
k__Fungi;p__Ascomycota;c__Leotiomycetes; o__Helotiales;f__Helotiales_fam_Incertae_sedis;g__Tetracladium
k__Fungi;p__Ascomycota;c__Dothideomycetes; o__Capnodiales;f__Davidiellaceae;g__Cladosporium
k__Fungi;p__Ascomycota;c__Dothideomycetes; o__Pleosporales;f__Leptosphaeriaceae;g__Leptosphaeria
k__Fungi;Other;Other;Other;Other;Other
k__Fungi;p__Ascomycota;c__Leotiomycetes; o__Helotiales;f__Helotiales_fam_Incertae_sedis;g__Cadophora
k__Fungi;p__Ascomycota;c__Leotiomycetes; o__Leotiomycetes_ord_Incertae_sedis;f__Myxotrichaceae;g__Pseudogymnoascus
k__Fungi;p__Ascomycota;c__Lecanoromycetes; o__Teloschistales;f__Physciaceae;g__Physciella
k__Fungi;p__Ascomycota;c__Pezizomycetes; o__Pezizales;f__Pyronemataceae;g__Sphaerosporella
b)
DADA2
PCo2 (12.47%)
PCo1 (16.3%)
Deblur
PCo2 (14.16%)
PCo1 (20.91%)
VSEARCH
PCo2 (12.46%)
PCo1 (15.64%)
DNACLUST
PCo2 (12.35%)
PCo1 (15.18%)
PlantType
Control
SmallSP
Time
T0
T1
c)
Observed OTUs/ASVs

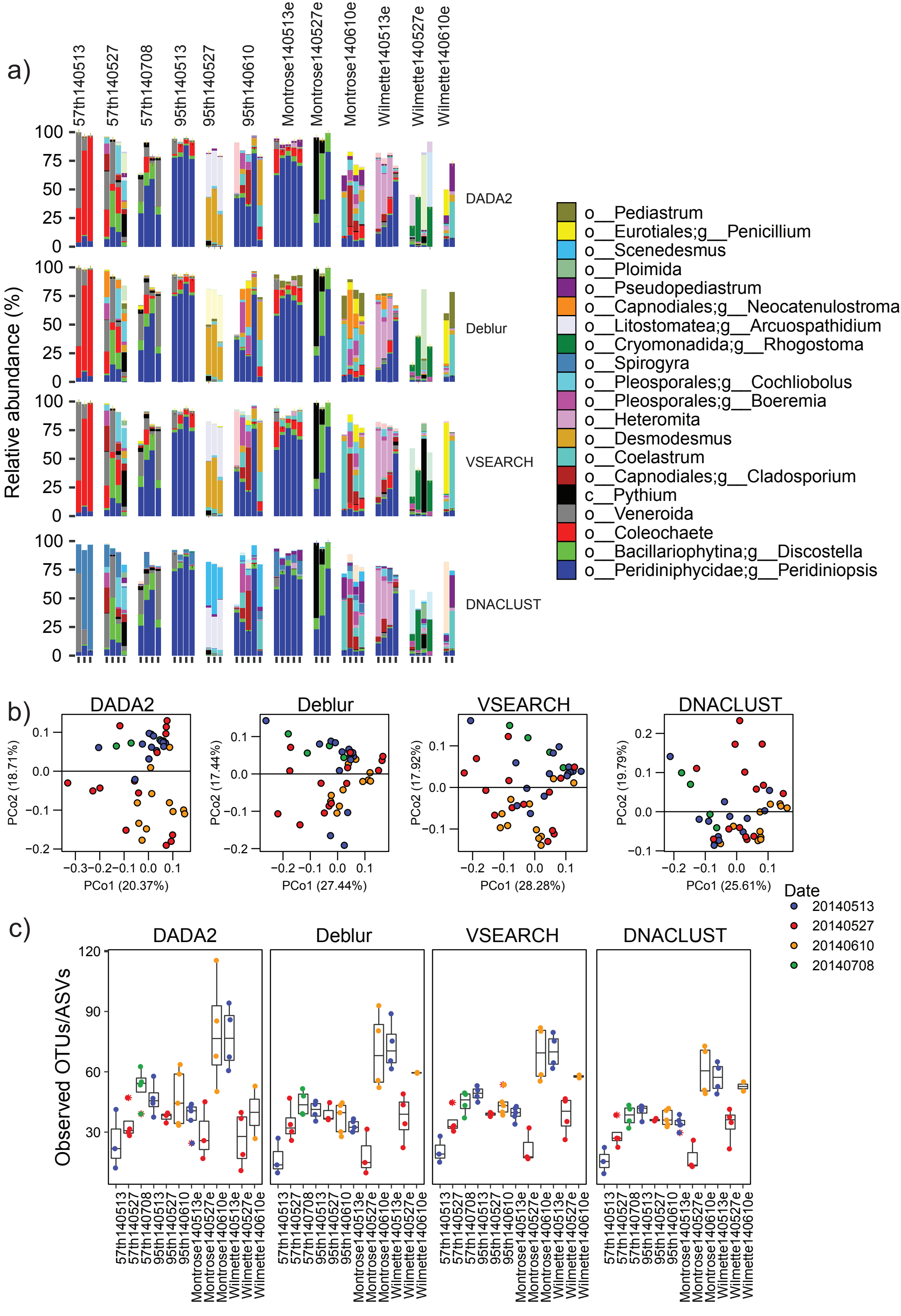

a)
57th140513
57th140527
57th140708
95th140513
95th140527
95th140610
Montrose140513e
Montrose140527e
Montrose140610e
Wilmette140513e
Wilmette140527e
Wilmette140610e
Relative abundance (%)
DADA2
Deblur
VSEARCH
DNACLUST
o__Pediastrum
o__Eurotiales;g__Penicillium
o__Scenedesmus
o__Ploimida
o__Pseudopediastrum
o__Capnodiales;g__Neocatenulostroma
o__Litostomatea;g__Arcuospathidium
o__Cryomonadida;g__Rhogostoma
o__Spirogyra
o__Pleosporales;g__Cochliobolus
o__Pleosporales;g__Boeremia
o__Heteromita
o__Desmodesmus
o__Coelastrum
o__Capnodiales;g__Cladosporium
c__Pythium
o__Veneroida
o__Coleochaete
o__Bacillariophytina;g__Discostella
o__Peridiniphycidae;g__Peridiniopsis
b)
DADA2
PCo1 (20.37%)
PCo2 (18.71%)
Deblur
PCo1 (27.44%)
PCo2 (17.44%)
VSEARCH
PCo1 (28.28%)
PCo2 (17.92%)
DNACLUST
PCo1 (25.61%)
PCo2 (19.79%)
Date
20140513
20140527
20140610
20140708
c)
Observed OTUs/ASVs

Figure S6

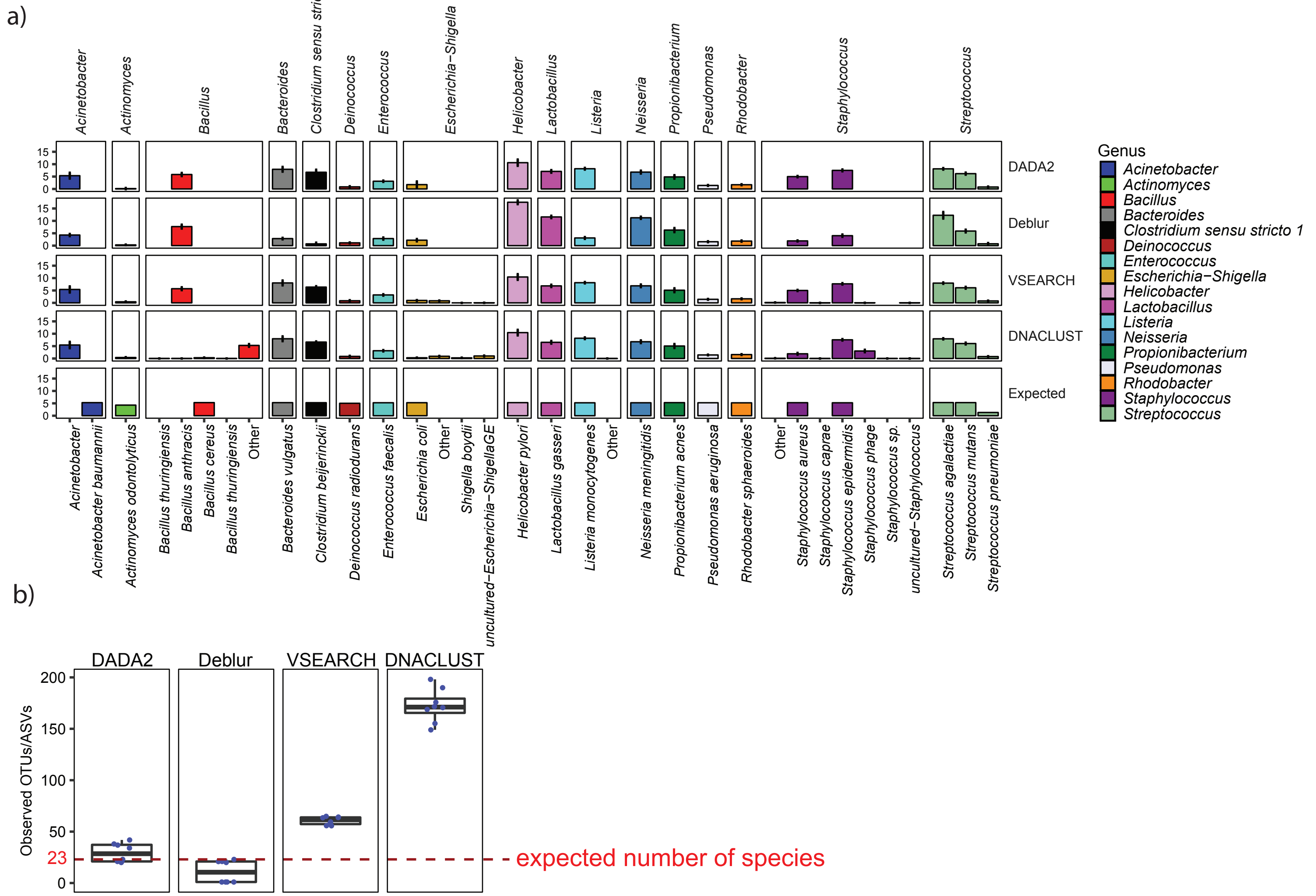

a)
Acinetobacter
Actinomyces
Bacillus
Bacteroides
Clostridium sensu stricto 1
Deinococcus
Enterococcus
Escherichia−Shigella
Helicobacter
Lactobacillus
Listeria
Neisseria
Propionibacterium
Pseudomonas
Rhodobacter
Staphylococcus
Streptococcus
DADA2
Deblur
VSEARCH
DNACLUST
Expected
0
5
10
15
Acinetobacter
Acinetobacter baumannii
Actinomyces odontolyticus
Bacillus thuringiensis
Bacillus anthracis
Bacillus cereus
Bacillus thuringiensis
Other
Bacteroides vulgatus
Clostridium beijerinckii
Deinococcus radiodurans
Enterococcus faecalis
Escherichia coli
Other
Shigella boydii
uncultured−Escherichia−ShigellaGE
Helicobacter pylori
Lactobacillus gasseri
Listeria monocytogenes
Other
Neisseria meningitidis
Propionibacterium acnes
Pseudomonas aeruginosa
Rhodobacter sphaeroides
Other
Staphylococcus aureus
Staphylococcus caprae
Staphylococcus epidermidis
Staphylococcus phage
Staphylococcus sp.
uncultured−Staphylococcus
Streptococcus agalactiae
Streptococcus mutans
Streptococcus pneumoniae
Genus
Acinetobacter
Actinomyces
Bacillus
Bacteroides
Clostridium sensu stricto 1
Deinococcus
Enterococcus
Escherichia−Shigella
Helicobacter
Lactobacillus
Listeria
Neisseria
Propionibacterium
Pseudomonas
Rhodobacter
Staphylococcus
Streptococcus
b)
DADA2
Deblur
VSEARCH
DNACLUST
Observed OTUs/ASVs
0
23
50
100
150
200
expected number of species

Figure S7

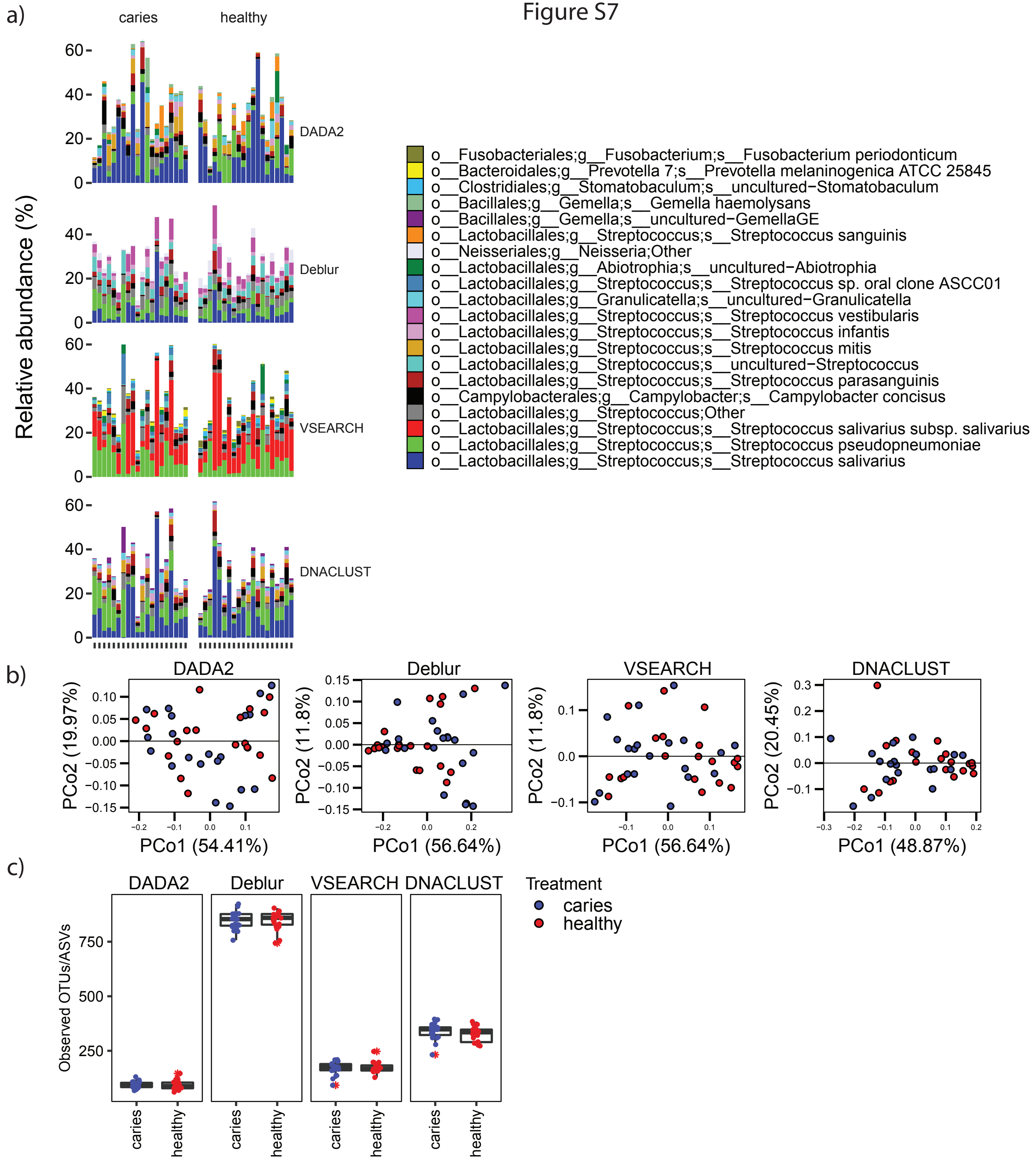

a)
caries
healthy
Relative abundance (%)
DADA2
Deblur
VSEARCH
DNACLUST
o__Fusobacteriales;g__Fusobacterium;s__Fusobacterium periodonticum
o__Bacteroidales;g__Prevotella 7;s__Prevotella melaninogenica ATCC 25845
o__Clostridiales;g__Stomatobaculum;s__uncultured−Stomatobaculum
o__Bacillales;g__Gemella;s__Gemella haemolysans
o__Bacillales;g__Gemella;s__uncultured−GemellaGE
o__Lactobacillales;g__Streptococcus;s__Streptococcus sanguinis
o__Neisseriales;g__Neisseria;Other
o__Lactobacillales;g__Abiotrophia;s__uncultured−Abiotrophia
o__Lactobacillales;g__Streptococcus;s__Streptococcus sp. oral clone ASCC01
o__Lactobacillales;g__Granulicatella;s__uncultured−Granulicatella
o__Lactobacillales;g__Streptococcus;s__Streptococcus vestibularis
o__Lactobacillales;g__Streptococcus;s__Streptococcus infantis
o__Lactobacillales;g__Streptococcus;s__Streptococcus mitis
o__Lactobacillales;g__Streptococcus;s__uncultured−Streptococcus
o__Lactobacillales;g__Streptococcus;s__Streptococcus parasanguinis
o__Campylobacterales;g__Campylobacter;s__Campylobacter concisus
o__Lactobacillales;g__Streptococcus;Other
o__Lactobacillales;g__Streptococcus;s__Streptococcus salivarius subsp. salivarius
o__Lactobacillales;g__Streptococcus;s__Streptococcus pseudopneumoniae
o__Lactobacillales;g__Streptococcus;s__Streptococcus salivarius
b)
DADA2
PCo2 (19.97%)
PCo1 (54.41%)
Deblur
PCo2 (11.8%)
PCo1 (56.64%)
VSEARCH
PCo2 (11.8%)
PCo1 (56.64%)
DNACLUST
PCo2 (20.45%)
PCo1 (48.87%)
c)
DADA2
Deblur
VSEARCH
DNACLUST
Observed OTUs/ASVs
Treatment
caries
healthy

Figure S8

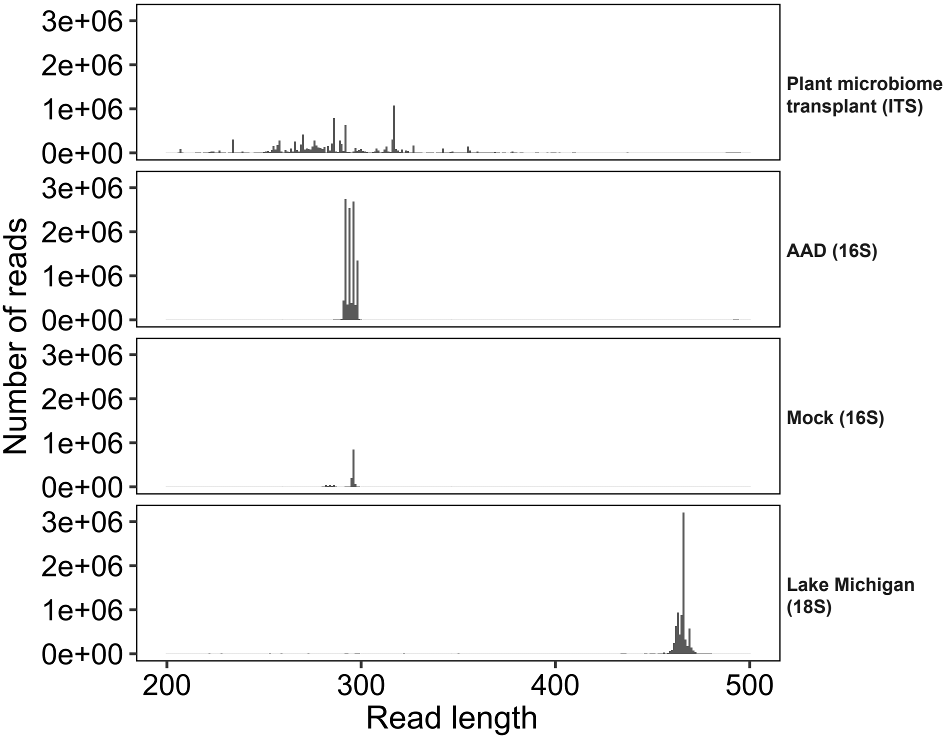
Number of reads
Read length
3e+06
2e+06
1e+06
0e+00
200
300
400
500
Plant microbiome transplant (ITS)
AAD (16S)
Mock (16S)
Lake Michigan (18S)
