# Supplementary material for: Systematic processing of ribosomal RNA gene amplicon sequencing data
Source: Gigascience. 2019 Dec 9;8(12):giz146. doi: 10.1093/gigascience/giz146 (PMC6901069; doi:10.1093/gigascience/giz146)
Supplement: giz146_Supplemental_Files [file giz146_supplemental_files.zip › additional_file_3.docx]

**QIIME2 commands used to process the mock community short reads sequencing data.**

QIIME2-Vsearch workflow

#!/bin/bash

#SBATCH --time=12:00:00

#SBATCH --nodes=1

#SBATCH --account=my_account

#SBATCH -n 4

#SBATCH --mem=24000

#SBATCH -o ./stdout.txt

#SBATCH -e ./stderr.txt

module unload nrc/R/3.4.0

module load nrc/qiime/2.2018.2

mkdir -p exported

# Import data

qiime tools import \

--type 'SampleData[PairedEndSequencesWithQuality]' \

--input-path raw_reads \

--source-format CasavaOneEightSingleLanePerSampleDirFmt \

--output-path demux-paired-end.qza

qiime cutadapt trim-paired \

--i-demultiplexed-sequences demux-paired-end.qza \

--p-front-f GTGCCAGCMGCCGCGGTAA \

--p-front-r GGACTACHVGGGTWTCTAAT \

--p-error-rate 0.1 \

--o-trimmed-sequences trimmed-seqs.qza \

--verbose

qiime vsearch join-pairs --i-demultiplexed-seqs trimmed-seqs.qza \

--o-joined-sequences trimmed-seqs-joined.qza

qiime quality-filter q-score-joined --i-demux trimmed-seqs-joined.qza \

--o-filter-stats filt_stats.qza \

--o-filtered-sequences trimmed-seqs-joined-filt.qza

qiime tools export --output-dir ./exported trimmed-seqs-joined-filt.qza

qiime vsearch dereplicate-sequences \

--i-sequences ./trimmed-seqs-joined-filt.qza \

--o-dereplicated-table dereplicated_table.qza \

--o-dereplicated-sequences dereplicated_seqs.qza

qiime vsearch cluster-features-de-novo \

--i-sequences dereplicated_seqs.qza \

--i-table dereplicated_table.qza \

--p-perc-identity 0.97 \

--p-threads 4 \

--o-clustered-table cluster_table.qza \

--o-clustered-sequences cluster_seqs.qza

qiime vsearch uchime-denovo \

--i-table cluster_table.qza \

--i-sequences cluster_seqs.qza \

--output-dir ./uchime_denovo

qiime tools export --output-dir uchime_denovo uchime_denovo/stats.qza

qiime tools export --output-dir uchime_denovo uchime_denovo/nonchimeras.qza

qiime tools export cluster_table.qza --output-dir cluster_table

biom convert -i cluster_table/feature-table.biom -o cluster_table/feature-table.tsv --to-tsv

cat uchime_denovo/dna-sequences.fasta | grep ">" | sed 's/>//' > uchime_denovo/non-chimeras.txt

head -n 2 cluster_table/feature-table.tsv > cluster_table/feature-table-nochimeras.tsv

cat cluster_table/feature-table.tsv | grep -f uchime_denovo/non-chimeras.txt >> cluster_table/feature-table-nochimeras.tsv

biom convert -i cluster_table/feature-table-nochimeras.tsv -o cluster_table/feature-table-nochimeras.biom --to-hdf5

qiime tools import --type FeatureTable[Frequency] --input-path cluster_table/feature-table-nochimeras.biom --output-path cluster_table/feature-table-nochimeras.qza

qiime vsearch uchime-ref \

--i-sequences uchime_denovo/nonchimeras.qza \

--i-table cluster_table/feature-table-nochimeras.qza \

--i-reference-sequences /project/6008026/databases/qiime2_dbs/broad_gold.qza

--output-dir uchime_denovo_ref

qiime tools export uchime_denovo_ref/nonchimeras.qza --output-dir uchime_denovo_ref/

cat uchime_denovo_ref/dna-sequences.fasta | grep ">" | sed 's/>//' > uchime_denovo_ref/non-chimeras.txt

head -n 2 cluster_table/feature-table-nochimeras.tsv > uchime_denovo_ref/feature-table.tsv

cat cluster_table/feature-table-nochimeras.tsv | grep -f uchime_denovo_ref/non-chimeras.txt >> uchime_denovo_ref/feature-table.tsv

biom convert -i uchime_denovo_ref/feature-table.tsv -o uchime_denovo_ref/feature-table.biom --to-hdf5

qiime tools import --type FeatureTable[Frequency] --input-path uchime_denovo_ref/feature-table.biom --output-path uchime_denovo_ref/feature-table.qza

qiime feature-table filter-features \

--i-table uchime_denovo_ref/feature-table.qza \

--p-min-frequency 25 \

--o-filtered-table uchime_denovo_ref/feature-table-filtered.qza

qiime tools export uchime_denovo_ref/feature-table-filtered.qza --output-dir uchime_denovo_ref_filtered

biom convert -i uchime_denovo_ref_filtered/feature-table.biom -o uchime_denovo_ref_filtered/feature-table.tsv --to-tsv

qiime feature-table filter-seqs \

--i-data uchime_denovo_ref/nonchimeras.qza \

--i-table uchime_denovo_ref/feature-table-filtered.qza \

--o-filtered-data uchime_denovo_ref/feature-sequence-filtered.qza

qiime tools export uchime_denovo_ref/feature-sequence-filtered.qza --output-dir uchime_denovo_ref_filtered

## alignment + Tree

qiime alignment mafft \

--i-sequences uchime_denovo_ref/feature-sequence-filtered.qza \

--o-alignment aligned-rep-seqs.qza

qiime alignment mask \

--i-alignment aligned-rep-seqs.qza \

--o-masked-alignment masked-aligned-rep-seqs.qza

qiime phylogeny fasttree \

--i-alignment masked-aligned-rep-seqs.qza \

--o-tree unrooted-tree.qza

qiime phylogeny midpoint-root \

--i-tree unrooted-tree.qza \

--o-rooted-tree rooted-tree.qza

# Beta div

# Rarefaction + betadiv + taxonomy

qiime feature-table rarefy --i-table uchime_denovo_ref/feature-table-filtered.qza --p-sampling-depth 1000 --o-rarefied-table ./table_1000.qza

rm -rf core-metrics-results

qiime diversity core-metrics-phylogenetic \

--i-phylogeny rooted-tree.qza \

--i-table table_1000.qza \

--p-sampling-depth 1000 \

--m-metadata-file mapping_file.tsv \

--output-dir core-metrics-results

qiime tools export --output-dir exported_wuf core-metrics-results/weighted_unifrac_pcoa_results.qza

qiime tools export --output-dir exported_wuf core-metrics-results/weighted_unifrac_distance_matrix.qza

qiime tools export --output-dir exported_bc core-metrics-results/bray_curtis_pcoa_results.qza

qiime tools export --output-dir exported_bc core-metrics-results/bray_curtis_distance_matrix.qza

# Classify taxonomy and create classic OTU table.

qiime feature-classifier classify-sklearn \

--i-classifier /project/xyz/databases/qiime2_dbs/silva-128-99-nb-classifier.qza \

--i-reads uchime_denovo_ref/feature-sequence-filtered.qza \

--o-classification taxonomy.qza

rm -rf taxtable

qiime taxa collapse \

--i-table table_1000.qza \

--i-taxonomy taxonomy.qza \

--p-level 6 \

--output-dir taxtable/

qiime tools export --output-dir taxtable/ taxtable/collapsed_table.qza

biom convert -i taxtable/feature-table.biom -o taxtable/feature-table.tsv --to-tsv

qiime tools export --output-dir exported table_1000.qza

qiime tools export --output-dir exported taxonomy.qza

### Here edit taxonomy.tsv to modify headers. Basically the first column header has to be changed to '#OTU ID' and the second column header to 'taxonomy'.

sed -i 's/Feature ID/#OTU ID/' exported/taxonomy.tsv

sed -i 's/Taxon/taxonomy/' exported/taxonomy.tsv

biom add-metadata -i exported/feature-table.biom -o exported/table-with-taxonomy.biom --observation-metadata-fp exported/taxonomy.tsv --sc-separated taxonomy

biom convert -i exported/table-with-taxonomy.biom -o exported/table-with-taxonomy.tsv --to-tsv --output-metadata-id='taxonomy' --header-key='taxonomy'

# alpha div

qiime diversity alpha \

--i-table uchime_denovo_ref/feature-table-filtered.qza \

--p-metric observed_otus \

--o-alpha-diversity observed_otus_vector.qza

qiime tools export --output-dir exported observed_otus_vector.qza

Qiime2-Deblur workflow

#!/bin/bash

#SBATCH --time=12:00:00

#SBATCH --nodes=1

#SBATCH --account=my_account

#SBATCH -n 1

#SBATCH --mem=12000

#SBATCH -o ./stdout.txt

#SBATCH -e ./stderr.txt

module unload nrc/R/3.4.0

module load nrc/qiime/2.2018.2

mkdir -p exported

# Import data

qiime tools import \

--type 'SampleData[PairedEndSequencesWithQuality]' \

--input-path raw_reads \

--source-format CasavaOneEightSingleLanePerSampleDirFmt \

--output-path demux-paired-end.qza

qiime cutadapt trim-paired \

--i-demultiplexed-sequences demux-paired-end.qza \

--p-front-f GTGCCAGCMGCCGCGGTAA \

--p-front-r GGACTACHVGGGTWTCTAAT \

--p-error-rate 0.1 \

--o-trimmed-sequences trimmed-seqs.qza \

--verbose

qiime vsearch join-pairs --i-demultiplexed-seqs trimmed-seqs.qza \

--o-joined-sequences trimmed-seqs-joined.qza

qiime quality-filter q-score-joined --i-demux trimmed-seqs-joined.qza \

--o-filter-stats filt_stats.qza \

--o-filtered-sequences trimmed-seqs-joined-filt.qza

qiime tools export --output-dir ./exported trimmed-seqs-joined-filt.qza

qiime deblur denoise-16S \

--p-trim-length 249 \

--p-sample-stats \

--p-indel-max 5 \

--p-min-reads 25 --p-min-size 0 --p-jobs-to-start 4 \

--i-demultiplexed-seqs trimmed-seqs-joined-filt.qza \

--o-representative-sequences rep-seqs.qza \

--o-table table.qza \

--output-dir deblur_out

qiime tools export --output-dir ./exported rep-seqs.qza

qiime tools import --type FeatureTable[Frequency] --input-path deblur_out/feature-table.biom --output-path deblur_out/feature-table.qza

rm -rf uchime_denovo

qiime vsearch uchime-denovo \

--i-table deblur_out/feature-table.qza \

--i-sequences rep-seqs.qza \

--output-dir ./uchime_denovo

qiime tools export --output-dir uchime_denovo uchime_denovo/stats.qza

qiime tools export --output-dir uchime_denovo uchime_denovo/nonchimeras.qza

qiime tools export deblur_out/feature-table.qza --output-dir deblur_out

biom convert -i deblur_out/feature-table.biom -o deblur_out/feature-table.tsv --to-tsv

cat uchime_denovo/dna-sequences.fasta | grep ">" | sed 's/>//' > uchime_denovo/non-chimeras.txt

head -n 2 deblur_out/feature-table.tsv > deblur_out/feature-table-nochimeras.tsv

cat deblur_out/feature-table.tsv | grep -f uchime_denovo/non-chimeras.txt >> deblur_out/feature-table-nochimeras.tsv

biom convert -i deblur_out/feature-table-nochimeras.tsv -o deblur_out/feature-table-nochimeras.biom --to-hdf5

qiime tools import --type FeatureTable[Frequency] --input-path deblur_out/feature-table-nochimeras.biom --output-path deblur_out/feature-table-nochimeras.qza

rm -rf uchime_denovo_ref

qiime vsearch uchime-ref \

--i-sequences uchime_denovo/nonchimeras.qza \

--i-table deblur_out/feature-table-nochimeras.qza \

--i-reference-sequences /project/6008026/databases/qiime2_dbs/broad_gold.qza \

--output-dir uchime_denovo_ref

qiime tools export uchime_denovo_ref/nonchimeras.qza --output-dir uchime_denovo_ref/

cat uchime_denovo_ref/dna-sequences.fasta | grep ">" | sed 's/>//' > uchime_denovo_ref/non-chimeras.txt

head -n 2 deblur_out/feature-table-nochimeras.tsv > uchime_denovo_ref/feature-table.tsv

cat deblur_out/feature-table-nochimeras.tsv | grep -f uchime_denovo_ref/non-chimeras.txt >> uchime_denovo_ref/feature-table.tsv

biom convert -i uchime_denovo_ref/feature-table.tsv -o uchime_denovo_ref/feature-table.biom --to-hdf5

qiime tools import --type FeatureTable[Frequency] --input-path uchime_denovo_ref/feature-table.biom --output-path uchime_denovo_ref/feature-table.qza

qiime feature-table filter-features \

--i-table uchime_denovo_ref/feature-table.qza \

--p-min-frequency 25 \

--o-filtered-table uchime_denovo_ref/feature-table-filtered.qza

qiime tools export uchime_denovo_ref/feature-table-filtered.qza --output-dir uchime_denovo_ref_filtered

biom convert -i uchime_denovo_ref_filtered/feature-table.biom -o uchime_denovo_ref_filtered/feature-table.tsv --to-tsv

qiime feature-table filter-seqs \

--i-data uchime_denovo_ref/nonchimeras.qza \

--i-table uchime_denovo_ref/feature-table-filtered.qza \

--o-filtered-data uchime_denovo_ref/feature-sequence-filtered.qza

qiime tools export uchime_denovo_ref/feature-sequence-filtered.qza --output-dir uchime_denovo_ref_filtered

## alignment + Tree

qiime alignment mafft \

--i-sequences uchime_denovo_ref/feature-sequence-filtered.qza \

--o-alignment aligned-rep-seqs.qza

qiime alignment mask \

--i-alignment aligned-rep-seqs.qza \

--o-masked-alignment masked-aligned-rep-seqs.qza

qiime phylogeny fasttree \

--i-alignment masked-aligned-rep-seqs.qza \

--o-tree unrooted-tree.qza

qiime phylogeny midpoint-root \

--i-tree unrooted-tree.qza \

--o-rooted-tree rooted-tree.qza

# Beta div

# Rarefaction + betadiv + taxonomy

qiime feature-table rarefy --i-table uchime_denovo_ref/feature-table-filtered.qza --p-sampling-depth 1000 --o-rarefied-table ./table_1000.qza

rm -rf core-metrics-results

qiime diversity core-metrics-phylogenetic \

--i-phylogeny rooted-tree.qza \

--i-table table_1000.qza \

--p-sampling-depth 1000 \

--m-metadata-file mapping_file.tsv \

--output-dir core-metrics-results

qiime tools export --output-dir exported_wuf core-metrics-results/weighted_unifrac_pcoa_results.qza

qiime tools export --output-dir exported_wuf core-metrics-results/weighted_unifrac_distance_matrix.qza

qiime tools export --output-dir exported_bc core-metrics-results/bray_curtis_pcoa_results.qza

qiime tools export --output-dir exported_bc core-metrics-results/bray_curtis_distance_matrix.qza

# Classify taxonomy and create classic OTU table.

qiime feature-classifier classify-sklearn \

--i-classifier /project/xyz/databases/qiime2_dbs/silva-128-99-nb-classifier.qza \

--i-reads uchime_denovo_ref/feature-sequence-filtered.qza \

--o-classification taxonomy.qza

rm -rf taxtable

qiime taxa collapse \

--i-table table_1000.qza \

--i-taxonomy taxonomy.qza \

--p-level 6 \

--output-dir taxtable/

qiime tools export --output-dir taxtable/ taxtable/collapsed_table.qza

biom convert -i taxtable/feature-table.biom -o taxtable/feature-table.tsv --to-tsv

qiime tools export --output-dir exported table_1000.qza

qiime tools export --output-dir exported taxonomy.qza

### Here edit taxonomy.tsv to modify headers. Basically the first column header has to be changed to '#OTU ID' and the second column header to 'taxonomy'.

sed -i 's/Feature ID/#OTU ID/' exported/taxonomy.tsv

sed -i 's/Taxon/taxonomy/' exported/taxonomy.tsv

biom add-metadata -i exported/feature-table.biom -o exported/table-with-taxonomy.biom --observation-metadata-fp exported/taxonomy.tsv --sc-separated taxonomy

biom convert -i exported/table-with-taxonomy.biom -o exported/table-with-taxonomy.tsv --to-tsv --output-metadata-id='taxonomy' --header-key='taxonomy'

# alpha div

qiime diversity alpha \

--i-table uchime_denovo_ref/feature-table-filtered.qza \

--p-metric observed_otus \

--o-alpha-diversity observed_otus_vector.qza

qiime tools export --output-dir exported observed_otus_vector.qza

Qiime2-DADA2 workflow

#!/bin/bash

#SBATCH --time=6:00:00

#SBATCH --nodes=1

#SBATCH --account=my_account

#SBATCH -n 1

#SBATCH --mem=32000

#SBATCH -o ./stdout.txt

#SBATCH -e ./stderr.txt

# Let Qiime2 manage its own version of R

module unload nrc/R/3.4.0

module load nrc/qiime/2.2018.2

mkdir -p exported

# Import data

qiime tools import \

--type 'SampleData[PairedEndSequencesWithQuality]' \

--input-path raw_reads.orig \

--source-format CasavaOneEightSingleLanePerSampleDirFmt \

--output-path demux-paired-end.qza

# DADA2

rm -rf dada2_output

qiime dada2 denoise-paired --i-demultiplexed-seqs demux-paired-end.qza \

--p-trunc-len-f 180 \

--p-trunc-len-r 180 \

--p-trim-left-f 20 \

--p-trim-left-r 20 \

--p-max-ee 2 \

--p-trunc-q 0 \

--p-n-threads 4 \

--verbose \

--output-dir dada2_output

qiime tools export --output-dir ./exported dada2_output/representative_sequences.qza

qiime tools export --output-dir ./dada2_output dada2_output/table.qza

biom convert -i dada2_output/feature-table.biom -o dada2_output/feature-table.tsv --to-tsv

qiime vsearch uchime-ref \

--i-sequences dada2_output/representative_sequences.qza \

--i-table dada2_output/table.qza \

--i-reference-sequences /project/6008026/databases/qiime2_dbs/broad_gold.qza \

--output-dir uchime_ref

# generate new feature table with non-chimera sequences only.

qiime tools export uchime_ref/nonchimeras.qza --output-dir uchime_ref/

cat uchime_ref/dna-sequences.fasta | grep ">" | sed 's/>//' > uchime_ref/non-chimeras.txt

head -n 2 cluster_table/feature-table-nochimeras.tsv > uchime_ref/feature-table.tsv

cat cluster_table/feature-table-nochimeras.tsv | grep -f uchime_ref/non-chimeras.txt >> uchime_ref/feature-table.tsv

biom convert -i uchime_ref/feature-table.tsv -o uchime_ref/feature-table.biom --to-hdf5

qiime tools import --type FeatureTable[Frequency] --input-path uchime_ref/feature-table.biom --output-path uchime_ref/feature-table.qza

# Filter the new feature table so that end table is in line as much as it can with the AmpliconTagger results.

qiime feature-table filter-features \

--i-table uchime_ref/feature-table.qza \

--p-min-frequency 25 \

--o-filtered-table uchime_ref/feature-table-filtered.qza

# Then generate new feature sequence file so that it contains sequence found in the feature-table-filtered.qza only.

qiime tools export uchime_ref/feature-table-filtered.qza --output-dir uchime_ref_filtered

biom convert -i uchime_ref_filtered/feature-table.biom -o uchime_ref_filtered/feature-table.tsv --to-tsv

# Generate sequence file

qiime feature-table filter-seqs \

--i-data uchime_ref/nonchimeras.qza \

--i-table uchime_ref/feature-table-filtered.qza \

--o-filtered-data uchime_ref_filtered/feature-sequence-filtered.qza

### alignment + Tree

qiime alignment mafft \

--i-sequences uchime_ref_filtered/feature-sequence-filtered.qza \

--o-alignment aligned-rep-seqs.qza

qiime alignment mask \

--i-alignment aligned-rep-seqs.qza \

--o-masked-alignment masked-aligned-rep-seqs.qza

qiime phylogeny fasttree \

--i-alignment masked-aligned-rep-seqs.qza \

--o-tree unrooted-tree.qza

qiime phylogeny midpoint-root \

--i-tree unrooted-tree.qza \

--o-rooted-tree rooted-tree.qza

## Betadiv + tax summary

qiime feature-table rarefy --i-table uchime_ref/feature-table-filtered.qza --p-sampling-depth 1000 --o-rarefied-table ./table_1000.qza

rm -rf core-metrics-results

qiime diversity core-metrics-phylogenetic \

--i-phylogeny rooted-tree.qza \

--i-table table_1000.qza \

--p-sampling-depth 1000 \

--m-metadata-file mapping_file.tsv \

--output-dir core-metrics-results

qiime tools export --output-dir ./exported core-metrics-results/weighted_unifrac_pcoa_results.qza

qiime tools export --output-dir exported core-metrics-results/weighted_unifrac_distance_matrix.qza

qiime feature-classifier classify-sklearn \

--i-classifier /project/xyz/databases/qiime2_dbs/silva-128-99-nb-classifier.qza \

--i-reads uchime_ref_filtered/feature-sequence-filtered.qza \

--o-classification taxonomy.qza

rm -rf taxtable

qiime taxa collapse \

--i-table table_1000.qza \

--i-taxonomy taxonomy.qza \

--p-level 6 \

--output-dir taxtable/

qiime tools export --output-dir taxtable/ taxtable/collapsed_table.qza

biom convert -i taxtable/feature-table.biom -o taxtable/feature-table.tsv --to-tsv

qiime tools export --output-dir exported table_1000.qza

qiime tools export --output-dir exported taxonomy.qza

### Here edit taxonomy.tsv to modify headers. Basically the first column header has to be changed to '#OTU ID' and the second column header to 'taxonomy'.

sed -i 's/Feature ID/#OTU ID/' exported/taxonomy.tsv

sed -i 's/Taxon/taxonomy/' exported/taxonomy.tsv

biom add-metadata -i exported/feature-table.biom -o exported/table-with-taxonomy.biom --observation-metadata-fp exported/taxonomy.tsv --sc-separated taxonomy

biom convert -i exported/table-with-taxonomy.biom -o exported/table-with-taxonomy.tsv --to-tsv --output-metadata-id='taxonomy' --header-key='taxonomy'

# alpha div

qiime diversity alpha \

--i-table uchime_ref/feature-table-filtered.qza \

--p-metric observed_otus \

--o-alpha-diversity observed_otus_vector.qza

qiime tools export --output-dir exported observed_otus_vector.qza
