# Supplementary material for: Systematic processing of ribosomal RNA gene amplicon sequencing data
Source: Gigascience. 2019 Dec 9;8(12):giz146. doi: 10.1093/gigascience/giz146 (PMC6901069; doi:10.1093/gigascience/giz146)
Supplement: giz146_Response_to_Reviewer_Comments_Original_Submission [file giz146_response_to_reviewer_comments_original_submission.pdf]

Author's Response To Reviewer Comments

Close

Reviewer #1: I thank the authors for addressing my comments and making changes to their manuscript. I also thank the authors for maintaining the list of commands, their SLURM scripts and the documentation of the tool. I believe the manuscript has benefited from these changes. However, please review the below responses.

Analysis of Mock communities:

As stated in lines 139 - 143 the analysis steps for each data analysis is provided in Additional file 2 and 3 with explicitly stating that QIIME2 commands are contained within the Additional file 3. While I refer to this file, I notice that the file (Additional file 3) is labeled as, "Qiime2 commands used to process the indoor microbiome data" which is not the same as the mock community dataset.

Answer: We thank the reviewer for pointing this inconsistency. We indeed uploaded the wrong file during the submission process. The file the reviewer examined is actually the "old" additional file 3 from the previous manuscript version. We made sure to provide the correct additional file 3 in the revised manuscript and we apologize for this.

Assuming Additional file 3 is the file with the parameters the authors used for processing the Mock community, I do not see a constant use of the same trimming parameters between different methods. For example: QIIME-deblur method allows reads to be 249 bp whereas QIIME-DADA2 method trims the reads to 150 bp. If these trimming parameters were followed, the discrepancy described in the lines, 154-160: of overestimated abundance of Staphylococcus is quite possible. Why are not all the trimming options been kept consistent?

Answer: The reason for this is because DADA2 does not allow to use merged paired-end amplicons. So basically, reads have to be submitted separately as R1 and R2 to DADA2, hence the different read length of 150 bp compared to the one used in Deblur which accepts merged amplicon reads.

I do notice the authors mention, "dada2 kept rejecting the staggered synthetic community library, I had to lower the parameters to make it accepts all the libraries".
If the authors could not get the QIIME2-DADA implementation work, can they test this against the stand-alone DADA2 version where there is a larger flexibility of choosing options than considered here.

Answer: We investigated why exactly our QIIME2-DADA2 workflow behaved that way and we found the correct parameters to make it work accordingly (as included in additional
file 3). We did our best to run DADA2 with parameters similar as much as we could to what was done in our pipeline.

There seems to be an inconsistency with respect to assigning taxonomy. They assign taxonomy using 2 different sets of Silva R128 training sets, one for AmpliconTagger and one for QIIME2 results. Why was this choice made?

Answer: Like stated above the reason to use QIIME2 here was to process the mock community data completely with three QIIME2 implementations which includes using the QIIME2 provided database and see if it compares with end results obtained by AmpliconTagger.

The authors in the paper state that, "We wish to emphasize that we do not present our methodology as a gold standard but rather a blueprint of an end-to-end open source working modular pipeline". This statement seems to be contrary to the decisions followed for analysis.

Answer: In this manuscript we showcase the capability of our pipeline by exposing end results of several datasets covering many data types processed with 4 different workflows (DADA2, Deblur, VSEARCH and DNACLUST), we believe that this objectively shows that our pipeline indeed provides a blueprint of an

end-to-end open source flexible/modular pipeline. At the reviewer's excellent suggestion in the previous round of revisions, we added an extensive documentation of our pipeline so others could implement their methodology as they see fit. Of course we think our methodology provides accurate results for the interpretation of amplicon sequence data, but that does not mean that we present our methodology as a gold standard and nowhere in the manuscript do we suggest that.

Figure 1b needs to be labeled to describe which portion of data was run in Amplicon tagger vs QIIME2.

Answer: OK, this has been addressed in the revised manuscript at lines 756-757.
Figure 1c: This figure is very difficult to read. Please re-order this figure especially the center panel where the dnaclust is placed above qiime2-dada2 results. They are not the same comparisons.

Answer: OK, this has been addressed in the revised manuscript. We believe figure 1 is now clearer.

Dnaclust comparison from a 3rd party is missing. DADA2 is missing from the tool list in Amplicontagger.

Answer: To the best of our knowledge, DNACLUST implementation from a third party pipeline is not available which explains its absence in the 3rd party validation section. We added this important fact in the manuscript at lines 154-155. DADA2 has been added from the tool list in the revised manuscript (line 692).

Also dada2 analysis has been included only for the mock communities and is missing from their analysis of other datasets. Why were these choices made?

Answer: Since DADA2 was not implemented in AmpliconTagger, in the manuscripts's previous iteration we did not use it for datasets other than the mock community. The use of DADA2 in the mock community has strictly been done to compare results of AmpliconTagger against results of the same data entirely processed with an established method – in this case QIIME2 with either DADA2, VSEARCH or Deblur as ASV/OTU generation method.
However, in line with the below comments from the reviewer, we implemented DADA2 in AmpliconTagger and now provide analyses done with DADA2 for all datasets investigated in the manuscript.

From Figure 1d) there is a large difference in the results for the staggered community results observed for AmpliconTagger vsearch v/s QIIME2-vsearch. Their vsearch implementation provides a result of 9 OTU's vs 25 - 30 OTU's in the QIIME2-vsearch results. Were same parameters used? Did they use the same version of vsearch in the comparisons?
Additionally, from figure 1D: Did the authors obtain a greater number of ASV's than in the mock community? This usually points to not having similar trimming parameters across different methods used.

Answer: The parameters in QIIME2-VSEARCH were modified so that they could as close as possible to what is used in AmpliconTagger-VSEARCH. The actual parameter that was critical and that we failed to properly include in the QIIME2 analyses of the previous iteration was the minimum reads cutoff per ASV or OTU which was of 25 for all AmpliconTagger's short reads analyses, but was unfortunately not applied for the QIIME2 workflows. This has been corrected in the revised version of the manuscript.
We re-ran all analyses and the number of ASVs for the mock community analyses are now much more consistent.

Table 3 provides the correlations between the different tested methods, however it would have been interesting to compare the AmpliconTagger vs QIIME2 results for deblur, vsearch. I think addition of this statistic will provide more useful information to the user of their tool than the current implementation.
Answer: OK we added this information in Table S3 in the revised manuscript and added discussion elements at lines 163-167.

Additionally, table 3 does not list the Mantel statistics for all the datasets. Why?

Answer: Table 3 missed the comparison from the QIIME2 processed results. As stated in the answer above, this information is now available in Table S3. Table 3 of the current iteration of the manuscript includes all other non-QIIME2 comparisons.

As a user and a reviewer, while I look at a tool and its performance: I look for the following parameters

to obtain confidence on the method,

i. Same trimming parameters across all comparisons,
ii. Same version of a method across comparisons,
iii. Same taxonomic assignment databases.
iv. Consistent results across different methods.

From following the results of the mock community analysis, I do not see their decisions in the mock community analysis satisfying the above criteria.

The discrepancies in the above steps makes it difficult to obtain confidence in the comparison carried out here.

Answer: This comment is in line with the reviewer's previous comments regarding this matter and we wish to clarify our reasoning (briefly stated in the manuscript at lines 130-134).
1- Following one of the reviewers' comment on the previous iteration of our manuscript, our objective with QIIME2 was to compare the end results of amplicon data from our method against an established pipeline, in this case QIIME2. In that context, the choice to use different parameters was unavoidable as the packages or scripts used to process the data (other than VSEARCH, DADA2 and Deblur) are inherently different in AmpliconTagger vs QIIME2. Besides, we wanted to see how AmpliconTagger's results compared against QIIME2 ran with standard parameters (i.e. identical or very close to what is described in the QIIME2 help web page) with three OTU/ASV generation methods supported in QIIME2. These parameters are probably what many users will use in "production" settings and confidently assessing that AmpliconTagger gives similar results to what is obtain with QIIME2 under these "standard" circumstances supports our method to be valid.
2- As stated in point 1) above, comparing our method with QIIME2 executed with identical parameters is impossible as our pipeline's methods are in the end different enough from the ones that QIIME2 implements that it is not possible to accomplish. For instance trimming and reads filtering parameters is performed differently in AmpliconTagger (tagsQC.pl) compared with what is done in QIIME2. As another example, there are no reads contaminants filtering steps in QIIME2 in contrast to our pipeline.
3- Regarding taxonomic database, we deliberately choose a database provided on QIIME2's website so that again we could compare analyses with the ones done entirely by QIIME2 – which includes using QIIME2's stock database. In that case, however, we also deliberately choose QIIME2's Silva 128 release (the same release that we used), but again, the methods (classifier software) and the way the training sets are built from the Silva 128 release are different.
4- We strongly believe that we demonstrated the results obtained with QIIME2 vs AmpliconTagger to be comparable and consistent. Moreover this consistency is robust to the aforementioned differences between the two methods (AmpliconTagger vs Qiime2).

Pacbio long reads analysis:

While the taxonomic profiles obtained show good results across all the comparisons, i.e., dnaclust, vsearch and deblur the number of ASV's for deblur does not inspire confidence in being available as a method for Pacbio analysis. I would suggest the author's disable deblur as an option for Pacbio due to the large discrepancy of the number of ASV's.

Answer: After examining more closely the output of the PacBio mock community data, we realized that CCS reads were provided in both 5◊3' and 3'◊5' orientation which affected downstream results. We entirely reprocessed the PacBio data mock community data from scratch by re-orienting the reads in the correct orientation. Moreover, we refined our methods and kept reads that had length between 1200 and 1600 bases which rejected many shorted and longer sequences that were probably artefacts. In consequence, the end results are much cleaner for all the 4 methods (Dnaclust, Vsearch, DADA2 and Deblur). Our results show that the results Deblur provides are now in line with what is generated by the other 3 packages and we wish to keep Deblur support in our pipeline. We also reprocessed the PacBio oral microbiome data, but in that case reads were already oriented in the 5'->3' direction.

Of all the above methods, none of them have been specifically designed for long reads as all of them were designed for short read data. I think this tool will hugely benefit from including DADA2 due to: a) It will provide users 2 OTU methods and 2 ASV methods as options; b) DADA2 is the only tool which has a defined Pacbio analysis.

The pre-print for DADA2 Pacbio: https://www.biorxiv.org/content/10.1101/392332v2.abstract.

If the authors are keenly interested to provide Pacbio processing, I would strongly suggest them to implement DADA2 in their tool which will give them a ASV method designed for Pacbio data analysis.

Answer: We thank the reviewer for this excellent suggestion. We added support for DADA2 PacBio data processing in our pipeline.

Figure S5:c has SNV's instead of ASV. Please fix this.
Answer: Done.

Minor comments:

Qiime2 is written as QIIME2.
Answer:Corrected.

dada2 is written as DADA2.
Answer: Corrected.

Additional Feedback on missing reviewer 2:

Getting external advice on reviewer 2's comments they don't feel you have sufficiently addressed the definition of "advanced customization" in mothur in lines 80-82 as this is only a quick comment on how Qiime offers limited options compared to R. As the reviewer says, mothur offers this functionality with many more options. How is AmpliconTagger advantageous over this?

Answer: We believe that the sentence at line 80-82 is a good example highlighting that many bioinformatics software packages have a wide range of options and that only a selection of these options are often included in pipeline wrappers. Moreover, nowhere do we suggest that the QIIME offers limited options over R, because we think this is not a relevant comparison: R is a programming language and QIIME is a pipeline supporting the execution of a range of software packages written in different languages. Regarding the reviewer's comment, we attentively screened Mothur's documentation and to the best of knowledge, it does not support DADA2 or ASV generation methods. In contrast to QIIME, Mothur is more of a stand-alone program that re-implements relevant microbial ecology algorithms into a stand-alone software. In that regard, it is true that our comment of limited advanced customization was not entirely appropriate in the case of Mothur – we therefore rephrased our statement at lines 80-82 of the revised manuscript.
Our pipeline is targeting an audience that is comfortable in code scripting, Linux and HPC environments (stated at lines 33-38 and 472-474) and as such it is advantageous over other pipelines because it allows, with some python scripting, to add literally any parameter to any software package (thoroughly explained in the User guide /Additional file 4).

If the primary objective of this report was to showcase AmpliconTagger, to do this more work is probably required to better demonstrate and discuss what advantage AmpliconTagger has over existing pipeline tools like Snakemake, Cromwell, or Nextflow for HPC pipelines, or even Nephele which offers easy interfaces and tools for microbiome analyses, without the need to install a whole system on a web interface.

Answer: The tools that the reviewer is referring to : Snakemake, Nextflow, Cromwell and Nextflow are declarative workflow description languages such as CWL (Common Workflow Language) or WDL (Workflow Description Language) are dedicated to providing a customizable framework to build bioinformatics pipelines. Such solutions are flexible and can help in pipeline implementation but do not offer the same level of features (for instance smart job restart/regeneration mechanism) and completely functional and modular 16S amplicon pipeline that was built and ready for production (our manuscript). We appreciate the reviewer's interest in comparing our method to the ones he mentions, but that would be an entirely separate manuscript in itself (i.e. comparing GenPipes with Snakemake, Nextflow, etc.). As such re-implementing our pipeline using these tools is outside the scoop of the work present here and would not add any benefits to the readers. Again, in this work, we present an exhaustive description and demonstration (including all the code) of a modular and flexible pipeline able to process any types of amplicon data and we provide all the results to back these claims.
Nephele is web (graphical) interface supporting a limited implementation of 16S and ITS data types processing. The authors realize that while this tool may be convenient in some situation, it does not in

any way compare with the flexibility that AmpliconTagger provides with its modular nature and command-line –based execution.
We are not sure what the reviewer is referring to when he states that we need to install a whole system on a web interface: AmpliconTagger is run directly from the command line on a Linux-based system.

Close
