# Supplementary material for: Systematic processing of ribosomal RNA gene amplicon sequencing data
Source: Gigascience. 2019 Dec 9;8(12):giz146. doi: 10.1093/gigascience/giz146 (PMC6901069; doi:10.1093/gigascience/giz146)
Supplement: giz146_Reviewer_1_Report_Original_Submission — Naga Betrapally -- 7/22/2019 Reviewed [file giz146_reviewer_1_report_original_submission.pdf]

Reviewer Report

Title: Systematic processing of rRNA gene amplicon sequencing data

Version: Original Submission Date: 7/22/2019

Reviewer name: Naga Betrapally

Reviewer Comments to Author:

I thank the authors for addressing my comments and making changes to their manuscript. I also thank the authors for maintaining the list of commands, their SLURM scripts and the documentation of the tool. I believe the manuscript has benefited from these changes. However, please review the below responses.

Analysis of Mock communities:

As stated in lines 139 - 143 the analysis steps for each data analysis is provided in Additional file 2 and 3 with explicitly stating that QIIME2 commands are contained within the Additional file 3. While I refer to this file, I notice that the file (Additional file 3) is labeled as, "Qiime2 commands used to process the indoor microbiome data" which is not the same as the mock community dataset.

Assuming Additional file 3 is the file with the parameters the authors used for processing the Mock community, I do not see a constant use of the same trimming parameters between different methods. For example: QIIME-deblur method allows reads to be 249 bp whereas QIIME-DADA2 method trims the reads to 150 bp. If these trimming parameters were followed, the discrepancy described in the lines, 154-160: of overestimated abundance of Staphylococcus is quite possible. Why are not all the trimming options been kept consistent?

I do notice the authors mention, "dada2 kept rejecting the staggered synthetic community library, I had to lower the parameters to make it accepts all the libraries".

If the authors could not get the QIIME2-DADA implementation work, can they test this against the stand-alone DADA2 version where there is a larger flexibility of choosing options than considered here.

There seems to be an inconsistency with respect to assigning taxonomy. They assign taxonomy using 2 different sets of Silva R128 training sets, one for AmpliconTagger and one for QIIME2 results. Why was this choice made?

The authors in the paper state that, "We wish to emphasize that we do not present our methodology as a gold standard but rather a blueprint of an end-to-end open source working modular pipeline". This statement seems to be contrary to the decisions followed for analysis.

Figure 1b needs to be labeled to describe which portion of data was run in Amplicon tagger vs QIIME2.

Figure 1c: This figure is very difficult to read. Please re-order this figure especially the center panel where the dnaclust is placed above qiime2-dada2 results. They are not the same comparisons.

Dnaclust comparison from a 3rd party is missing. DADA2 is missing from the tool list in Amplicontagger. Also dada2 analysis has been included only for the mock communities and is missing from their analysis of other datasets. Why were these choices made?

From Figure 1d) there is a large difference in the results for the staggered community results observed for AmpliconTagger vsearch v/s QIIME2-vsearch. Their vsearch implementation provides a result of 9

OTU's vs 25 - 30 OTU's in the QIIME2-vsearch results. Were same parameters used? Did they use the same version of vsearch in the comparisons?
Additionally, from figure 1D: Did the authors obtain a greater number of ASV's than in the mock community? This usually points to not having similar trimming parameters across different methods used.
Table 3 provides the correlations between the different tested methods, however it would have been interesting to compare the AmpliconTagger vs QIIME2 results for deblur, vsearch. I think addition of this statistic will provide more useful information to the user of their tool than the current implementation.
Additionally, table 3 does not list the Mantel statistics for all the datasets. Why?
As a user and a reviewer, while I look at a tool and its performance: I look for the following parameters to obtain confidence on the method,

i. Same trimming parameters across all comparisons,
ii. Same version of a method across comparisons,
iii. Same taxonomic assignment databases.
iv. Consistent results across different methods.

From following the results of the mock community analysis, I do not see their decisions in the mock community analysis satisfying the above criteria.
The discrepancies in the above steps makes it difficult to obtain confidence in the comparison carried out here.
Pacbio long reads analysis:
While the taxonomic profiles obtained show good results across all the comparisons, i.e., dnaclust, vsearch and deblur the number of ASV's for deblur does not inspire confidence in being available as a method for Pacbio analysis. I would suggest the author's disable deblur as an option for Pacbio due to the large discrepancy of the number of ASV's.
Of all the above methods, none of them have been specifically designed for long reads as all of them were designed for short read data. I think this tool will hugely benefit from including DADA2 due to: a) It will provide users 2 OTU methods and 2 ASV methods as options; b) DADA2 is the only tool which has a defined Pacbio analysis.
The pre-print for DADA2 Pacbio: https://www.biorxiv.org/content/10.1101/392332v2.abstract.
If the authors are keenly interested to provide Pacbio processing, I would strongly suggest them to implement DADA2 in their tool which will give them a ASV method designed for Pacbio data analysis.
Figure S5:c has SNV's instead of ASV. Please fix this.
Minor comments:
Qiime2 is written as QIIME2.
dada2 is written as DADA2.

Methods

Are the methods appropriate to the aims of the study, are they well described, and are necessary controls included? Choose an item.

Conclusions

Are the conclusions adequately supported by the data shown? Choose an item.

Reporting Standards

Does the manuscript adhere to the journal's guidelines on minimum standards of reporting? Choose an item.

Choose an item.

Statistics

Are you able to assess all statistics in the manuscript, including the appropriateness of statistical tests used? Choose an item.

Quality of Written English

Please indicate the quality of language in the manuscript: Choose an item.

Declaration of Competing Interests

Please complete a declaration of competing interests, considering the following questions:

- Have you in the past five years received reimbursements, fees, funding, or salary from an organisation that may in any way gain or lose financially from the publication of this manuscript, either now or in the future?
- Do you hold any stocks or shares in an organisation that may in any way gain or lose financially from the publication of this manuscript, either now or in the future?
- Do you hold or are you currently applying for any patents relating to the content of the manuscript?
- Have you received reimbursements, fees, funding, or salary from an organization that holds or has applied for patents relating to the content of the manuscript?
- Do you have any other financial competing interests?
- Do you have any non-financial competing interests in relation to this paper?

If you can answer no to all of the above, write 'I declare that I have no competing interests' below. If your reply is yes to any, please give details below.

I declare that I have no competing interests'

I agree to the open peer review policy of the journal. I understand that my name will be included on my report to the authors and, if the manuscript is accepted for publication, my named report including any attachments I upload will be posted on the website along with the authors' responses. I agree for my report to be made available under an Open Access Creative Commons CC-BY license (http://creativecommons.org/licenses/by/4.0/). I understand that any comments which I do not wish to

be included in my named report can be included as confidential comments to the editors, which will not be published.

Choose an item.

To further support our reviewers, we have joined with Publons, where you can gain additional credit to further highlight your hard work (see: https://publons.com/journal/530/gigascience). On publication of this paper, your review will be automatically added to Publons, you can then choose whether or not to claim your Publons credit. I understand this statement.

Yes Choose an item.
